# SRSF1 regulates primordial follicle formation and number determination during meiotic prophase I

**DOI:** 10.1186/s12915-023-01549-7

**Published:** 2023-03-08

**Authors:** Longjie Sun, Zheng Lv, Xuexue Chen, Chaofan Wang, Pengbo Lv, Lu Yan, Shuang Tian, Xiaomei Xie, Xiaohong Yao, Jingjing Liu, Zhao Wang, Haoshu Luo, Sheng Cui, Jiali Liu

**Affiliations:** 1 State Key Laboratory of Farm Animal Biotech Breeding, College of Biological Sciences, China Agricultural University, Beijing, 100193 China; 2 College of Veterinary Medicine, Yangzhou University, Yangzhou, Jiangsu 225009 China

**Keywords:** SRSF1, Alternative splicing, Oocyte, Meiosis, Primary ovarian insufficiency

## Abstract

**Background:**

Ovarian folliculogenesis is a tightly regulated process leading to the formation of functional oocytes and involving successive quality control mechanisms that monitor chromosomal DNA integrity and meiotic recombination. A number of factors and mechanisms have been suggested to be involved in folliculogenesis and associated with premature ovarian insufficiency, including abnormal alternative splicing (AS) of pre-mRNAs. Serine/arginine-rich splicing factor 1 (SRSF1; previously SF2/ASF) is a pivotal posttranscriptional regulator of gene expression in various biological processes. However, the physiological roles and mechanism of SRSF1 action in mouse early-stage oocytes remain elusive. Here, we show that SRSF1 is essential for primordial follicle formation and number determination during meiotic prophase I.

**Results:**

The conditional knockout (cKO) of *Srsf1* in mouse oocytes impairs primordial follicle formation and leads to primary ovarian insufficiency (POI). Oocyte-specific genes that regulate primordial follicle formation (e.g., *Lhx8*, *Nobox*, *Sohlh1*, *Sohlh2*, *Figla*, *Kit*, *Jag1*, and *Rac1*) are suppressed in newborn *Stra8-GFPCre Srsf1*^Fl/Fl^ mouse ovaries. However, meiotic defects are the leading cause of abnormal primordial follicle formation. Immunofluorescence analyses suggest that failed synapsis and an inability to undergo recombination result in fewer homologous DNA crossovers (COs) in the *Srsf1* cKO mouse ovaries. Moreover, SRSF1 directly binds and regulates the expression of the POI-related genes *Six6os1* and *Msh5* via AS to implement the meiotic prophase I program.

**Conclusions:**

Altogether, our data reveal the critical role of an SRSF1-mediated posttranscriptional regulatory mechanism in the mouse oocyte meiotic prophase I program, providing a framework to elucidate the molecular mechanisms of the posttranscriptional network underlying primordial follicle formation.

**Supplementary Information:**

The online version contains supplementary material available at 10.1186/s12915-023-01549-7.

## Background

High-quality gametes are critical for successful reproduction [1]. Functional oocytes are derived from successful folliculogenesis in the ovaries, which includes primordial follicle formation; recruitment into the growing pool to form primary, secondary, and tertiary follicles; ovulation; and subsequent corpus luteum formation [2]. The size and quality of the surviving pool of primordial follicles are essential determinants of female fecundity and reproductive lifespan [3]. To maintain quality, diverse organisms have evolved three quality control mechanisms for eliminating meiocytes with defects in meiotic recombination or SPO11-linked DNA double-strand break (DSB) accumulation, namely, meiotic silencing, the synapsis checkpoint, and the DNA damage checkpoint [4]. During meiotic prophase I, defective meiosis results in POI due to early exhaustion of the follicle pool.

With the widespread application of next-generation sequencing (NGS), the genetic spectrum of POI has been expanded, especially by the recent identification of novel meiosis-related genes [5–7]. The cohesin complex regulates sister chromatid cohesion and synaptonemal complex (SC) formation and is composed of the meiosis-specific subunits STAG3, RAD21L, and SMC1β and the nonspecific subunits SMC3 and REC8 [8]. STAG3 aberrations have been identified to cause a rare monogenic type of POI [9–18]. The chromosome axis forms a platform for SC assembly, which plays a central role in homologous pairing, recombination, and chromosome segregation. The central elements of SCs include SYCE1-3, SIX6OS1, and TEX12. Mutations in SYCE1 and SIX6OS1 have been identified in POI patients [19–23]. Notably, the abnormal AS of MEIOB, STAG3, MSH4, and MCM9 pre-mRNAs has been observed in human POI [15, 24–26]. However, the mechanisms that regulate pre-mRNA splicing during the progression of meiotic prophase I in mouse oocytes are poorly understood.

SRSF1 is a pivotal posttranscriptional regulator of gene expression in various biological processes [27, 28]. Unfortunately, homozygous *Srsf1* knockout mice exhibit early embryonic lethality [29]. Therefore, conditional *Srsf1* deletion mouse models have been applied in studies of its role in the heart and thymus [29–33]. Interestingly, single-cell RNA sequencing has demonstrated that the expression of SRSF1 first decreases and then increases during the oocyte meiotic prophase I program [34]. However, the physiological roles of SRSF1 during oocyte meiotic prophase I are still largely unclear. This study shows that conditional knockout of *Srsf1* in mouse oocytes impairs primordial follicle formation and leads to POI. We further verified that SRSF1 directly binds and regulates *Six6os1* and *Msh5* expression via AS, which is critical for implementing the meiotic prophase I program.

## Results

### SRSF1 deficiency leads to premature ovarian insufficiency

**Fig. 1:**
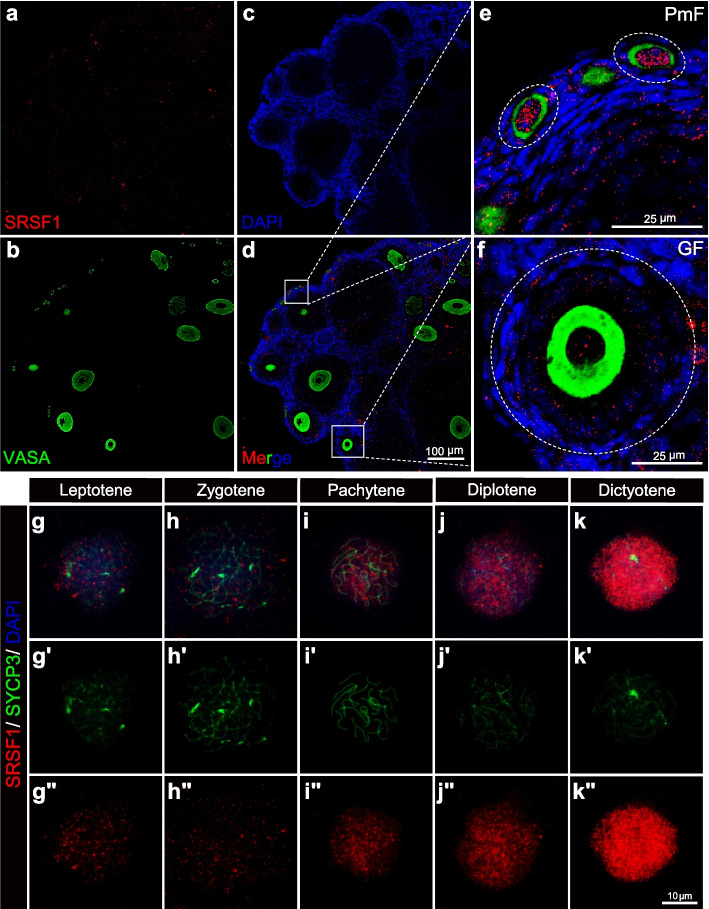
Dynamic localization of SRSF1 in mouse oocytes. **a–f** SRSF1 is highly expressed in oocytes of the primordial follicle. Immunostaining was performed using VASA and SRSF1 antibodies from adult mouse ovaries. DNA was stained with DAPI. **a–d** Scale bar, 100 μm. The **e** primordial follicle (PmF) and **f** grown follicle (GF) are shown in the magnified views. Scale bar, 25 μm. **g–k** The dynamic localization of SRSF1 to the oocyte meiotic prophase I program. Co-immunostaining was performed using SYCP3 and SRSF1 antibodies from 15.5 dpc, 17.5 dpc, 18.5 dpc, and 1 dpp oocyte surface spreading. DNA was stained with DAPI. **gʹ–kʹ** The meiotic stages of the oocytes were determined by SYCP3 staining (green). **gʺ–kʺ** The dynamic localization of SRSF1(red) during the oocyte meiotic prophase I program. Scale bar, 10 μm

The results of SRSF1 and VASA co-staining revealed that SRSF1 was expressed in the nuclei of oocytes and granulosa cells of the adult mouse ovary. Intriguingly, SRSF1 was highly expressed in the nucleus of the primordial follicle oocyte (Fig. 1a − f). To investigate the role of SRSF1 in POI, the expression pattern of SRSF1 during ovarian development and ageing was evaluated. Intriguingly, SRSF1 expression was reduced in the primordial follicle oocytes of ageing ovaries (Additional file 1: Fig. S1). These data suggest that SRSF1 may contribute to physiological ovarian ageing. To further study the detailed expression pattern of SRSF1 in primordial follicle formation, we observed oocytes at different meiotic stages in mouse ovaries by co-immunostaining with antibodies against SRSF1 and synaptonemal complex protein 3 (SYCP3). Interestingly, SRSF1 expression was high in leptotene spermatocytes and further increased in pachytene spermatocytes (Fig. 1g − k), suggesting that SRSF1 plays an essential role in forming primordial follicles.

**Fig. 2:**
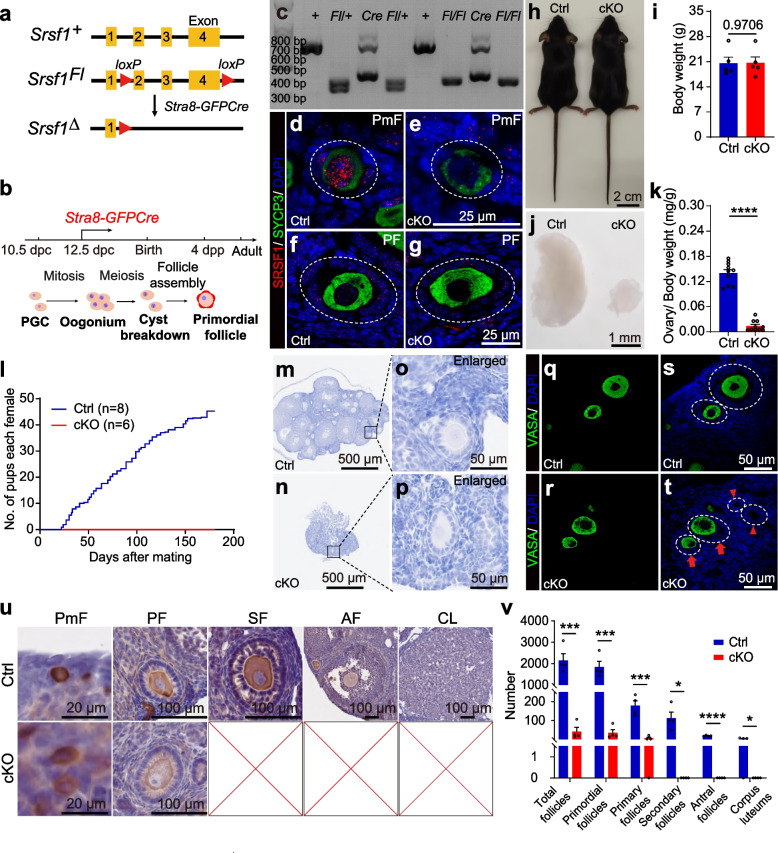
SRSF1 plays critical roles in follicle development and female fertility. **a** *Stra8-GFPCre* mice were crossed with *Srsf1*^*Fl/Fl*^ mice to generate *Srsf1* cKO mice. Cre-mediated deletion removed exons 2, 3, and 4 of *Srsf1* and generated a null protein allele. **b** Schematic diagram of Cre enzyme expression showing the spatiotemporal patterns of major cellular events in mouse early phase ovaries. **c** Genotyping PCRs were performed using *Stra8-GFPCre* and *Srsf1* primers. **d–g** Co-immunostaining of SRSF1 and VASA in adult Ctrl and cKO ovaries. PmF, primordial follicle; PF, primary follicle. Scale bar, 25 μm. **h, i** Normal body weight in cKO mice. The body sizes and weights of adult Ctrl and cKO mice are shown as the mean ± SEM. *n* = 5. **j, k** Ovarian atrophy in adult cKO mice. Ovary sizes and weights of adult Ctrl and cKO mice are shown as the mean ± SEM. *n* = 5. **i** Fertility test results showing an infertility phenotype in the cKO females (*n* = 8) compared to that in the Ctrl mice (*n* = 6) during 180 days of mating. **m–p** Haematoxylin stained ovary sections from adult Ctrl and cKO mice are shown. **m, n** Scale bar, 500 μm. **o, p** Magnified views are shown. Scale bar, 50 μm. **q–t** Immunostaining of VASA in adult Ctrl and cKO ovaries. DNA was stained with DAPI. Red arrows indicate abnormal follicles of oocytes deviating to one side. Red arrowheads indicate oocyte-free follicles. Scale bar, 50 μm. **u, v** Folliculogenesis was arrested in the cKO ovaries. **u** Immunostaining of VASA in adult Ctrl and cKO ovaries. DNA was stained with haematoxylin. PmF, primordial follicle; Scale bar, 20 μm. PF, primary follicle; SF, secondary follicle; AF, antral follicle; CL, corpus luteum; Scale bar, 100 μm. **v** Counting of the significant mouse stages of folliculogenesis. *n* = 4. Significance was determined by unpaired Student’s *t* test; detailed *P* value *P* ≥ 0.05, *P < 0.05, ***P < 0.001, ****P < 0.0001. The error bar represents the mean ± SEM

To define the specific involvement of SRSF1 in primordial follicle formation, we studied the physiological roles of SRSF1 in vivo using a mouse model. Considering that global SRSF1 knockout is lethal in mice [29], we used *Srsf1* cKO mice in which *Srsf1* is deleted at the time of meiosis initiation, starting at 12.5 days post-coitus (dpc), by the *Stra8-GFPCre* transgene [35, 36] (Fig. 2a, b). We used a conditional allele of *Srsf1* (*Srsf1*^Fl^) in which exons 2, 3, and 4 of *Srsf1* are flanked by two *loxP* sites (Fig. 2a). By crossing *Srsf1*^Fl^ and *Stra8-GFPCre* mice, we obtained *Stra8-GFPCre Srsf1*^Fl/Fl^ mice with *Srsf1* deletion in oocytes (Fig. 2a, c). We verified the absence of the SRSF1 protein in all postnatal oocytes by co-immunofluorescence analysis with SRSF1 and VASA antibodies (Fig. 2d − g).

The adult cKO mice were normal in size (Fig. 2h, i), but the size of their ovaries was significantly reduced (Fig. 2j, k). To further confirm the effect of the loss of *Srsf1* on female fertility, a breeding experiment was performed, and the data indicated that the absence of *Srsf1* led to complete infertility in cKO females (Fig. 2l). Histological examination of cKO ovary sections revealed that folliculogenesis was arrested (Fig. 2m − p). Immunostaining of VASA indicated the absence or asymmetric deviation of oocytes in cKO ovaries (Fig. 2q − t). Follicle count data based on VASA immunohistochemistry analysis showed that the follicles in cKO ovaries were primarily arrested at the primary follicle stage (Fig. 2u, v). Together, these results indicate that SRSF1 is critical for follicle development.

### SRSF1 is required for mouse primordial follicle formation

**Fig. 3:**
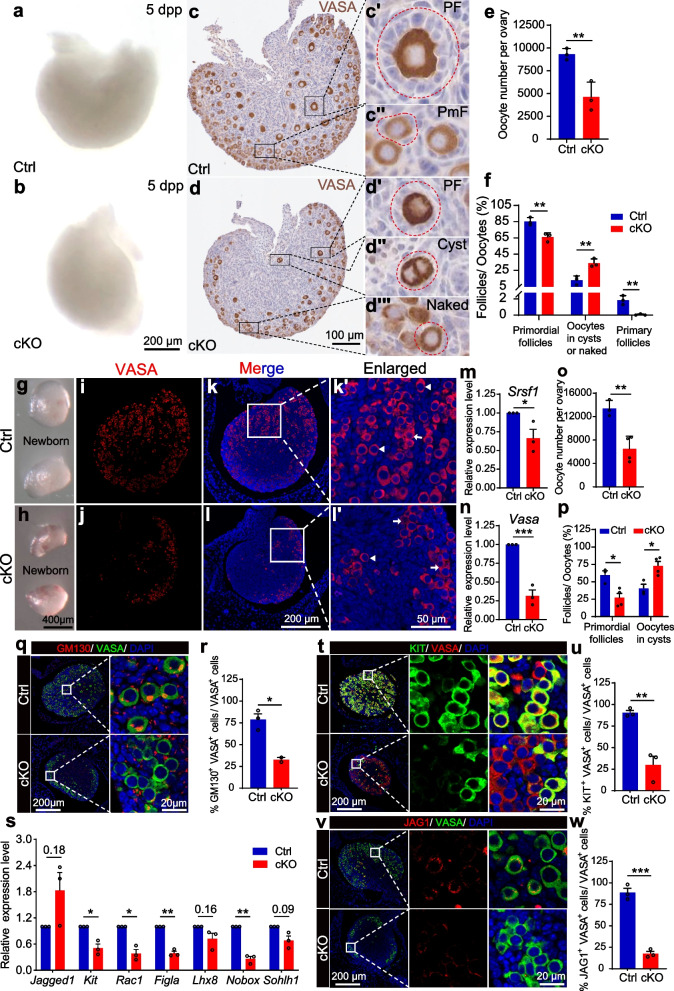
SRSF1 is required for mouse primordial follicle formation. **a, b** Ovarian atrophy in 5 dpp cKO mice. **c, d** Immunostaining of VASA in 5 dpp ovaries. PF, primary follicle (**cʹ, dʹ**), PmF, primordial follicle (**cʹʹ**)**,** cyst oocytes (**dʹʹ**)**,** naked oocyte (**dʹʹʹ**). **e** Quantification of total oocytes per 5 dpp ovary. **f** Quantification of primordial follicles, primary follicles, and oocytes in cysts or naked per 5 dpp ovary. *n* = 3. **g, h** Ovarian atrophy in newborn cKO mice. **i–l** Immunostaining of VASA in newborn ovaries. **kʹ, lʹ** The primordial follicle and oocytes in cysts are shown in magnified views. Arrowheads, PmF; arrows, cyst oocytes. **m** The mRNA level of *Srsf1* decreased in newborn cKO ovaries. **n** The mRNA level of *Vasa* decreased in newborn cKO ovaries. **o** Quantification of total oocytes per newborn ovary. Ctrl, *n* = 3; cKO, *n* = 4. **p** Percentages of primordial follicles and oocytes in cysts per newborn ovary. Ctrl, *n* = 3; cKO, *n* = 4. **q** Co-immunostaining of GM130 and VASA in newborn ovaries. **r** Percentage of double-positive cells in newborn ovaries. *n* = 2. **s** *Srsf1* deficiency downregulated expression of various genes (e.g., *Kit*, *Rac1*, *Figla*, *Nobox*). **t** Co-immunostaining of KIT and VASA in newborn ovaries. **u** Percentage of double-positive cells in newborn cKO ovaries. *n* = 3. **v** Co-immunostaining of JAG1 and VASA in newborn ovaries. **w** Percentage of double-positive cells is shown in newborn cKO ovaries. *n* = 3. Real-time qPCR data were normalized to *Gapdh* (**n, m**) or *Vasa *(**s**). *n* = 3. Significance was determined by unpaired Student’s *t* test; detailed *P* value *P* ≥ 0.05, *P < 0.05, ***P < 0.001, ****P < 0.0001. The error bar represents the mean ± SEM. Scale bar, 20 μm (**q **right,** t **magnified,** v **magnified left), 50 μm (**kʹ, lʹ**), 100 μm (**c, d**), 200 μm (**a, d, i–l, q **left,** t **left,** v **left), 400 μm (**g, h**)

To confirm the effect of the loss of *Srsf1* on POI, we examined mouse ovaries at 5 days post-partum (dpp), a timepoint at which the primordial follicle pool is successfully established [34]. Tissue analyses revealed that the cKO mice exhibited a reduced ovary size (Fig. 3a, b). Oocyte count data based on VASA immunohistochemistry analysis showed that the primordial follicle pool was consistently reduced in cKO ovaries (Fig. 3e). We verified that the numbers of naked oocytes or oocytes in the cysts were increased, but the numbers of primordial and primary follicles were reduced, in cKO ovaries (Fig. 3c, d, f). Together, these results suggest dysfunctional primordial follicle formation and number determination in cKO ovaries.

The specific involvement of SRSF1 in primordial follicle formation and number determination was further verified by the observation of mild ovarian atrophy in newborn cKO mice, in which the primordial follicle pool is being established (Fig. 3g, h). RT–qPCR showed that the expression of *Srsf1* was significantly decreased in cKO ovaries (Fig. 3m), and co-immunofluorescence analysis with SRSF1 and VASA antibodies showed an absence of the SRSF1 protein in all oocytes (Fig. 2d − g). Interestingly, VASA immunostaining revealed more oocytes in cysts in cKO ovaries (Fig. 3i − l). RT–qPCR results showed that the expression of *Vasa* was significantly decreased in the cKO ovaries, suggesting that the total number of oocytes was reduced (Fig. 3n). Additionally, count data showed that the numbers of both total oocytes and primary follicles were reduced in cKO ovaries (Fig. 3o, p). However, the number of oocytes in cysts was significantly increased (Fig. 3p). Abnormal primordial follicular pool formation was further confirmed by the observation of GM130 immunofluorescence in the ovaries of newborn cKO mice [37]. Our results revealed that the number of double-positive cells was reduced in newborn cKO ovaries, suggesting that SRSF1 is essential for cyst breakdown and primordial follicle formation (Fig. 3q, r). To understand the molecular mechanisms underlying these phenotypes, we examined vital genes that regulate primordial follicle formation by RT–qPCR and found that their expression was suppressed (Fig. 3s). Co-immunostaining of VASA and KIT or JAG1 showed that the number of double-positive cells was reduced in the ovaries of newborn cKO mice (Fig. 3t − w). These data suggest that vital genes that regulate primordial follicle formation are suppressed in the ovaries of newborn cKO mice.

### Loss of SRSF1 impairs meiotic progression in oocytes

**Fig. 4:**
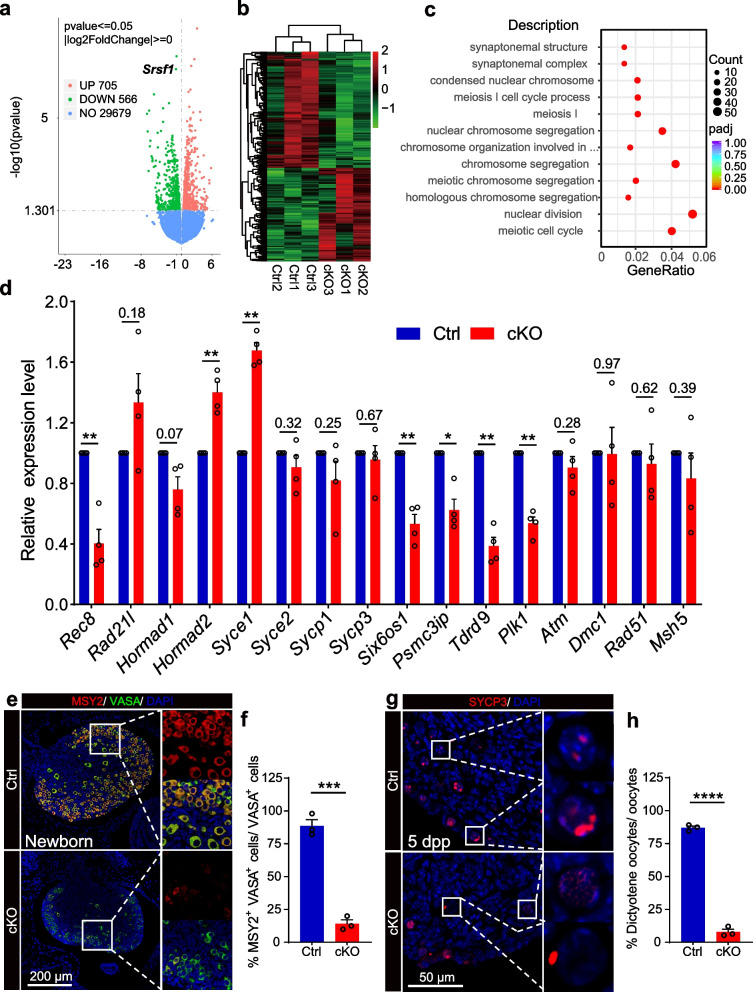
SRSF1 is required for meiotic progression. **a** Volcano map displaying the distribution of differentially expressed genes from RNA-seq data. The abscissa in the figure represents the gene fold change in cKO and Ctrl mouse ovaries. |log2FoldChange|≥ 0. The ordinate indicates the significance of gene expression differences between cKO and Ctrl mouse ovaries. *P* ≤ 0.05. Upregulated genes are shown in red dots, and downregulated genes are shown in green dots. **b** Cluster heatmap of differentially expressed genes. The abscissa is the genotype, and the ordinate is the normalized FPKM (fragments per kilobase million) value of the differentially expressed gene. Red indicates a higher expression level, while green indicates a lower expression level. **c** Scatter plot of GO enrichment analysis of the 1271 differentially expressed genes. The 12 most effective terms were selected to draw a scatter diagram for display. **d** The misregulation of meiosis-related genes in 16.5 dpc cKO ovaries. Real-time qPCR data were normalized to *Gapdh*. The value in newborn Ctrl ovaries was set as 1.0, and the relative value in the ovaries of newborn cKO mice is indicated. *n* = 4. **e** Co-immunostaining of MSY2 and VASA in newborn Ctrl and cKO ovaries. DNA was stained with DAPI. Scale bar, 200 μm. **f** Percentage of double-positive cells is shown as the mean ± SEM. *n* = 3. **g** Immunostaining of SYCP3 in 5 dpp Ctrl and cKO ovaries. DNA was stained with DAPI. The dictyotene oocytes of Ctrl ovaries are shown in magnified views. Abnormal oocytes of cKO ovaries are shown in the magnified views. Scale bar, 50 μm. **h** Percentage of dictyotene oocytes is shown as the mean ± SEM in 5 dpp ovaries. *n* = 3. Significance was determined by unpaired Student’s *t* test; detailed *P* value *P* ≥ 0.05, *P < 0.05, ***P < 0.001, ****P < 0.0001. The error bar represents the mean ± SEM

To investigate the molecular consequences of SRSF1 depletion in primordial follicle formation, we isolated mRNA from control (Ctrl) and cKO ovaries at 16.5 dpc and performed RNA sequencing. RNA-seq analyses identified 566 downregulated and 705 upregulated genes in cKO ovaries at 16.5 dpc (Fig. 4a, b; Additional file 2: Table 1). Surprisingly, Gene Ontology (GO) term enrichment indicated abnormal meiosis in cKO mouse oocytes (Fig. 4c). Next, we validated the abnormal expression of meiosis-related genes (downregulated: *Rec8*, *Six6os1*, *Psmc3ip*, *Tdrd9*, and *Plk1*; upregulated: *Hormad2* and *Syce1*) by RT–qPCR (Fig. 4d). To assess meiotic progression in cKO ovaries, MSY2 immunostaining was employed as a marker of the diplotene stage in newborn mouse ovaries (Fig. 4e) [38]. Double-positive cell counting results showed that the number of diplotene oocytes was reduced in cKO ovaries (Fig. 4f). To further evaluate this phenotype, we performed SYCP3 immunofluorescence analysis in 5 dpp mouse ovaries (Fig. 4g). Normal meiotic arrest in the dictyate stage is crucial for primordial follicle formation. It can be identified by the presence of two to four visible nucleolus signals after staining with an SYCP3 antibody [39]. Dictyate oocyte count data revealed that SRSF1 deficiency impaired meiotic progression to the diplotene stage in oocytes (Fig. 4h). These results suggest that the few oocytes in cKO ovaries that reached the diplotene stage could not develop further to the dictyate stage.

### SRSF1 deficiency leads to defects in synapsis and crossover recombination in cKO ovaries

**Fig. 5:**
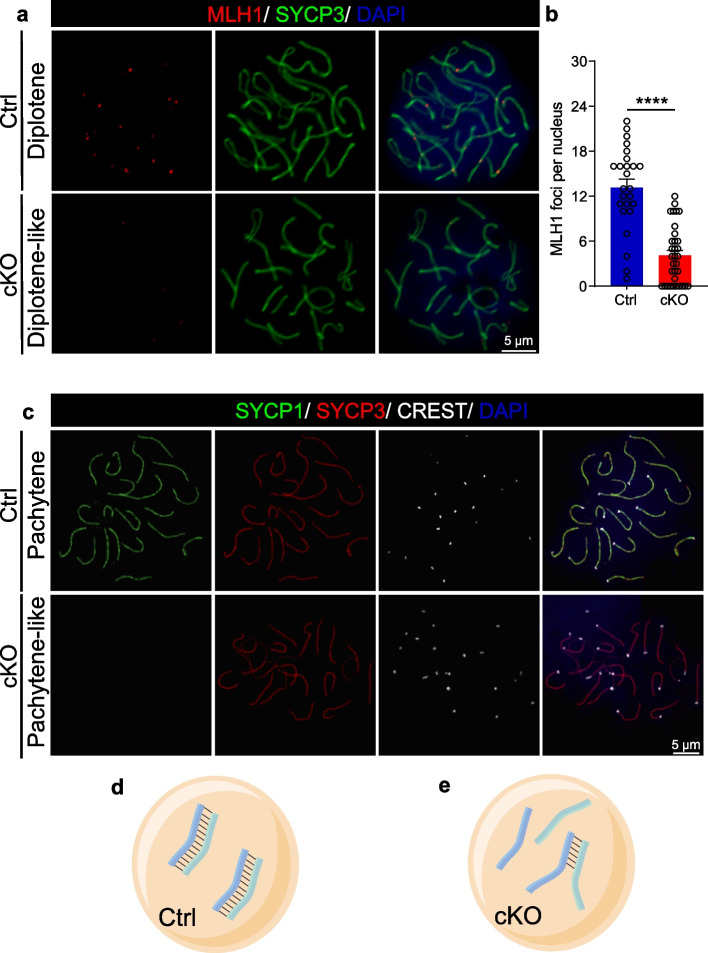
SRSF1 deficiency impairs chromosome synapsis and the formation of COs at meiotic prophase I. **a** Co-immunostaining of MLH1 and SYCP3 in newborn Ctrl and cKO oocytes. DNA was stained with DAPI. Scale bar, 5 μm. **b** The number of COs, marked by MLH1 foci (red), was significantly reduced in cKO diplotene-like oocytes compared with Ctrl oocytes. Twenty-five Ctrl oocytes and thirty-three cKO oocytes were obtained from 3 animals. Significance was determined by unpaired Student’s *t* test; ****P < 0.0001. The error bar represents the mean ± SEM. **c** Localization of SYCP1, SYCP3, and CREST in newborn Ctrl and cKO oocytes. DNA was stained with DAPI. Scale bar, 5 μm. Diagrams illustrate aberrant synapsis in Ctrl (**d**) and cKO (**e**) oocytes

The GO term enrichment results showed abnormal homologous chromosome segregation in cKO mouse oocytes (Fig. 4c). The COs cause the exchange of homologous DNA and establish the physical connections between homologues required for proper chromosome segregation [40]. MutL homologue 1 (MLH1) has been recognized as a classic marker of COs [41–43]. Therefore, the distribution of MLH1 foci was evaluated by MLH1 and SYCP3 immunostaining in newborn mouse oocyte surface spreads (Fig. 5a). The counts of MLH1 foci showed that the formation of COs was reduced in diplotene oocytes (Fig. 5b). The final and significant purpose of meiotic recombination is the formation of COs [44, 45]. To probe the nature of meiotic recombination in cKO ovaries, we examined chromosomal synapsis through oocyte surface spread analysis in newborn mouse ovaries. Aberrant synapsis was frequently observed in cKO oocytes based on the localization of synaptonemal complex protein 1 (SYCP1), SYCP3, and CREST (Fig. 5c). Surprisingly, SYCP1 could not be loaded onto chromosomes in cKO mouse oocytes (Fig. 5c). The provided schematic diagram shows the failure of synapsis in cKO oocytes (Fig. 5d, e). These data suggest that the failure of synapsis and the inability to undergo homologous recombination (HR) resulted in fewer COs in cKO oocytes.

### SRSF1 is essential for the resection of SPO11-linked DNA double-strand breaks (DSBs)

**Fig. 6:**
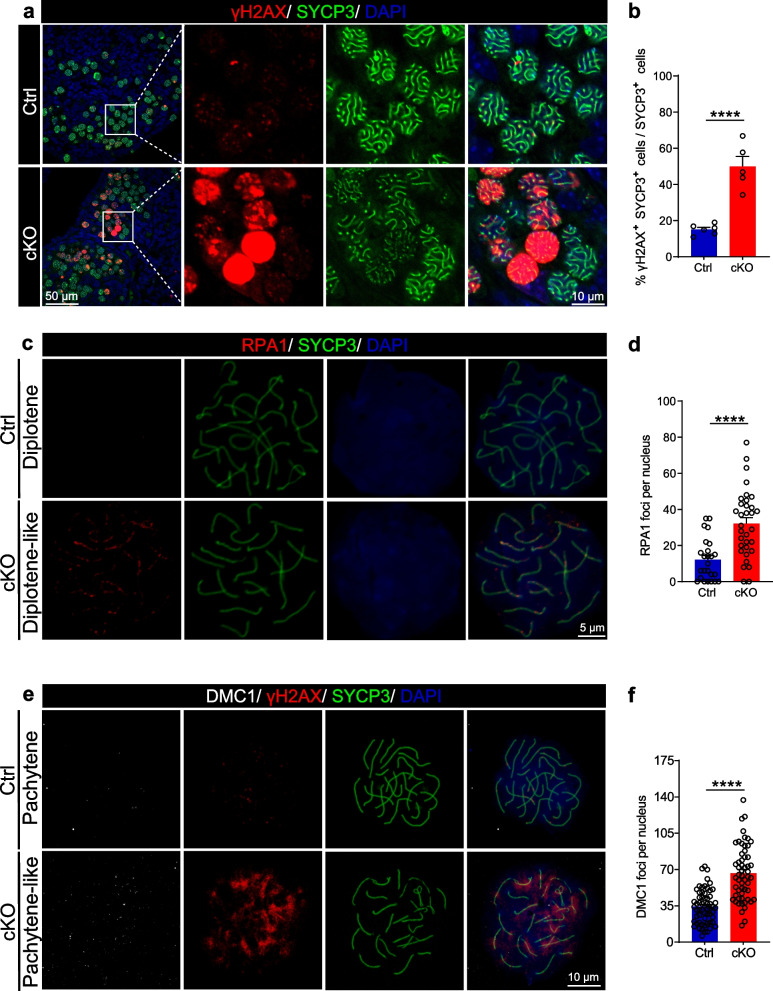
SRSF1 is necessary for the resection of DSBs. **a** Co-immunostaining of γH2AX and SYCP3 in 18.5 dpc Ctrl and cKO ovaries. DNA was stained with DAPI. Scale bar of the top panel, 50 μm. Scale bar of the rest panel, 10 μm. **b** Percentage of double-positive cells is shown as the mean ± SEM in 18.5 dpc ovaries. Ctrl, *n* = 6; cKO, *n* = 5. **c** Co-immunostaining of RPA1 and SYCP3 in newborn Ctrl and cKO oocytes. DNA was stained with DAPI. Scale bar, 5 μm. **d** In cKO diplotene-like oocytes, the number of RPA1 foci (red) was maintained at a relatively high level. Twenty-five Ctrl oocytes and thirty-three cKO oocytes were obtained from 3 animals. **e** Localization of DMC1, γH2AX, and SYCP3 in 18.5 dpc Ctrl and cKO oocytes. DNA was stained with DAPI. Scale bar, 10 μm. **f** In cKO pachytene oocytes, the number of DMC1 foci (white) was maintained at a relatively high level. Sixty Ctrl oocytes and fifty-six cKO oocytes were obtained from 4 animals. Significance was determined by unpaired Student’s *t* test; detailed *P* value *P* ≥ 0.05, *P < 0.05, ***P < 0.001, ****P < 0.0001. The error bar represents the mean ± SEM

DSBs formed during meiosis recruit phosphorylated histone H2AX (γH2AX) [46]. Therefore, we monitored the progression of meiotic recombination in 17.5 dpc ovaries by the co-immunostaining with γH2AX and SYCP3. The immunostaining results revealed distinct γH2AX signals in cKO oocytes, whereas few oocytes in Ctrl ovaries showed positive γH2AX staining (Fig. 6a). The double-positive cell counting data confirmed that some DSBs were not repaired correctly in cKO oocytes (Fig. 6b). Replication protein A (RPA) is a single-strand DNA-binding heterotrimeric complex composed of RPA1, RPA2, and RPA3 that is essential for meiotic recombination [47]. We examined the localization of RPA1 as a representative of the RPA complex by nuclear spread analysis in newborn mouse ovaries. The co-immunostaining of RPA1 and SYCP3 showed that many RPA complexes remained uncleared in the diplotene stage in cKO oocytes (Fig. 6c, d). RecA-like proteins DMC1 and RAD51 form a nucleoprotein filament on ssDNA within DSBs to aid in the search and invasion of the homologous partner for successful recombination and synapsis. We further explored the distribution of proteins involved in recombination and DSB repair. Interestingly, the localization of DMC1, γH2AX, and SYCP3 showed that significant DMC1 foci were present on chromosomes in the pachytene stage in cKO oocytes (Fig. 6e). The DMC1 focus count data revealed an increase in recombinase on chromosomes in the pachytene stage in cKO oocytes (Fig. 6f). However, the recombinase had lost its ability to effect on DNA repair. These observations suggest that SRSF1 is essential for the resection of SPO11-linked DSBs in meiotic prophase.

### SRSF1 directly regulates the splicing of the POI-related genes *Msh5* and *Six6os1*

**Fig. 7:**
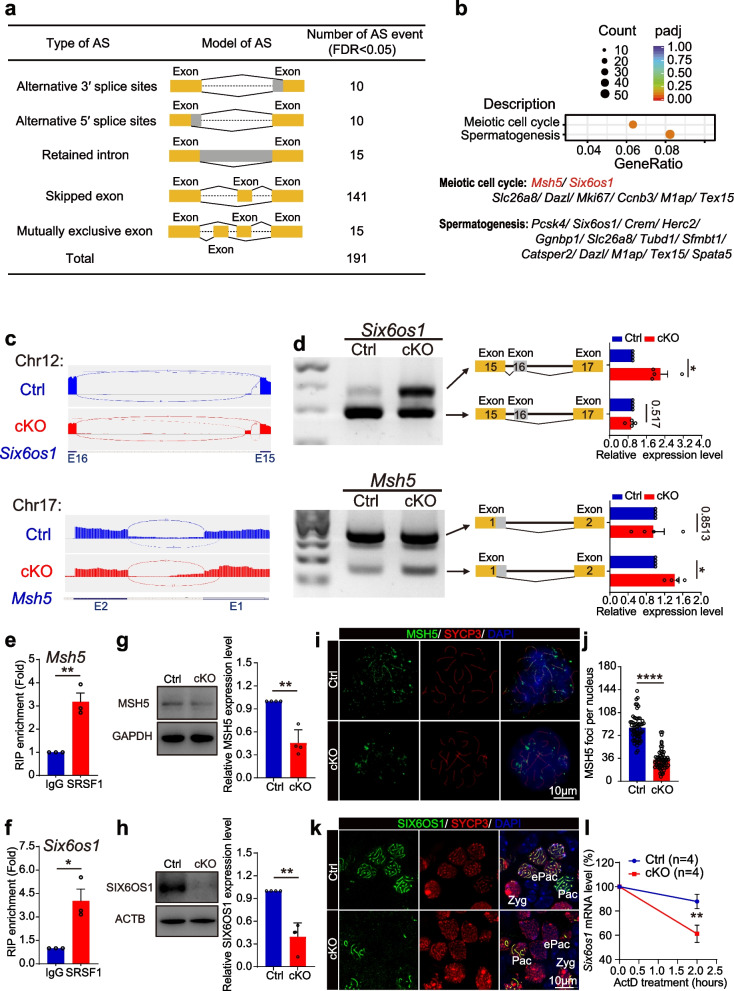
SRSF1 directly regulates the splicing of *Msh5* and *Six6os1*. **a** Five AS events were significantly affected by SRSF1-deficient oocytes. rMATS (3.2.5) software was used to analyse AS events (FDR < 0.05). FDR, false discovery rate calculated from the *P* value. **b** Scatter plot of the GO enrichment analysis of the 191 affected AS events. **c** A schematic of the regulation of the splicing of *Msh5* and *Six6os1.* Integrative Genomics Viewer (IGV, version 2.10.2) was used to visualize and confirm AS events in RNA-seq data. E, exon. **d** The ectopic splicing of *Msh5* and *Six6os1* in cKO ovaries was analysed by RT–PCR (*n* = 4 per group). The scheme and cumulative data on the percentage of the indicated fragment are shown accordingly. **e, f** SRSF1 directly regulated the expression of *Msh5* and *Six6os1* by RIP–qPCR in 16.5 dpc mouse ovaries. *n* = 3. **g, h** Western blotting of MSH5 and SIX6OS1 expression in 17.5 dpc Ctrl and cKO ovaries. GAPDH (**g**) or ACTB (**h**) served as a loading control. *n* = 4. **i** Localization of MSH5 and SYCP3 in 17.5 dpc Ctrl and cKO oocytes. DNA was stained with DAPI. Scale bar, 10 μm.** j** The number of MSH5 foci (green) was significantly reduced in cKO pachytene oocytes compared with Ctrl oocytes. Sixty Ctrl oocytes and sixty cKO oocytes were obtained from 4 animals. **k** Co-immunostaining of SIX6OS1 and SYCP3 in 17.5 dpc Ctrl and cKO ovaries. DNA was stained with DAPI. Zyg, zygotene; ePac, early pachytene; Pac, pachytene. Scale bar, 10 μm. **l** The expression of *Six6os1* in 16.5 dpc cKO ovaries after ActD treatment at different times. *n* = 4. Significance was determined by unpaired Student’s *t* test; detailed *P* value *P* ≥ 0.05, *P < 0.05, ***P < 0.001, ****P < 0.0001. The error bar represents the mean ± SEM

RNA-seq analyses showed 191 AS events that were identified as significantly affected (FDR < 0.05) in 16.5 dpc cKO ovaries (Additional file 3: Table 2). Among the 191 affected AS events, most (141) were classified as skipped exons (SEs). In addition, ten AS events were categorized as alternative 5′ splice sites (A5SSs), 10 as alternative 3′ splice sites (A3SSs), 15 as mutually exclusive exons (MXEs), and 15 as retained introns (RIs) (Fig. 7a). The GO enrichment analysis of the alternatively spliced genes revealed that eleven meiosis-related genes showed alterations in AS forms (Fig. 7b). At least two (*Six6os1* and *Msh5*) of these genes have been associated with POI [19, 20, 48]. We then visualized the different types of AS based on RNA-seq data by using Integrative Genomics Viewer (IGV, 2.10.2) software (Fig. 7c). RT–PCR results showed that the pre-mRNAs of *Six6os1* and *Msh5* in cKO mouse oocytes exhibited abnormal AS (Fig. 7d). The results of RIP–qPCR showed that SRSF1 could bind to the pre-mRNAs of *Msh5* and *Six6os1* (Fig. 7e, f). Interestingly, Western blotting and immunostaining revealed that the protein levels of MSH5 and SIX6OS1 were significantly suppressed (Fig. 7g − k; Additional file 4: Fig. S2a, b). Additionally, abnormal AS changed the mRNA decay rates of *Six6os1* (Fig. 7l) but not *Msh5* (Additional file 4: Fig. S2c) in cKO mouse ovaries.

## Discussion

### Abnormal follicle stock formation induces POI

The primordial follicle pool that forms perinatally is a female’s sole lifelong source of oocytes [49]. POI is determined by the exhaustion of the primordial follicle pool in the ovaries [5]. In cKO ovaries, we found more oocytes in cysts but a reduced number of total oocytes and primordial follicles. Our data suggest that cyst breakdown and primordial follicle formation are disrupted in cKO oocytes. Similar phenotypes have been observed following the loss of oocyte-specific genes (e.g., *Lhx8*, *Nobox*, *Sohlh1*, *Sohlh2*, *Figla*, *Kit*, *Jag1*, and *Rac1*) [50–56]. This study showed that most oocyte-enriched genes that regulate primordial follicle formation are suppressed in the ovaries of newborn cKO mice (Fig. 3s; Additional file 5: Fig. S3).

Interestingly, although the level of JAG1 protein was significantly reduced (Fig. 3v, w), there was no significant change in the *Jag1* mRNA level in the ovaries of newborn cKO mice (Fig. 3s). We speculate that SRSF1 acts as an essential splicing factor for *Jag1* and other regulatory factors involved in primordial follicle formation. However, meiotic defects are the leading cause of abnormal primordial follicle formation.

### Meiotic defects impair primordial follicle formation

Routine meiotic arrest at the dictyate stage is crucial for correcting DNA damage so that sufficient follicle numbers are retained in the ovary to ensure optimal fertility for an adequate period and prevent the transmission of genetic defects to the next generation [57]. Previous studies have shown that meiosis-related genes (e.g., *Stag3*, *Syce1*, *Spidr*, *Psmc3ip*, *Hfm1*, *Msh4*, *Msh5*, *Mcm8*, *Mcm9*, *Csb-Pgbd3*, and *Nup107*) play essential roles in the assembly of primordial follicles [5]. Co-immunostaining with MSY2 and SYCP3 antibodies showed that the few oocytes that reached the diplotene stage in cKO ovaries could not develop further to the dictyate stage (Fig. 4e-h). Additionally, the abnormal expression of meiosis-related genes (e.g., *Rec8*, *Six6os1*, *Psmc3ip*, *Tdrd9*, *Plk1*, *Hormad2*, and *Syce1*) impaired oocyte meiotic progression (Fig. 4d). These results suggest that meiotic defects led to abnormal dictyate arrest during primordial follicle formation.

### Abnormal AS of *Msh5* and *Six6os1* causes meiotic defects

SRSF1 directly binds and regulates interferon regulatory factor 7 (*Irf7*) and interleukin-27 receptor subunit alpha (*Il27ra*) expression via AS in the late stage of thymocyte development [30]. In addition, SRSF1 directly binds and regulates the AS of *Myb* pre-mRNA in invariant natural killer T cells. Furthermore, SRSF1 promotes cell proliferation, survival, and invasion in glioma tissues and cell lines by specifically switching the AS of myosin IB (MYO1B) [58]. Nevertheless, the physiological roles of SRSF1 during meiosis are still largely unclear. One key finding of this study is that SRSF1 directly regulates the AS of *Msh5* and *Six6os1* associated with POI during meiotic prophase I. In addition, in SRSF1-deficient oocytes, the expression of MSH5 protein levels is significantly reduced leading to POI.

MSH5 heterodimers play an essential role in the HR-based repair of DSBs [59]. Accordingly, the loss of either gene in mice results in meiotic disruption prior to the pachytene stage, including incomplete synapsis, persistence of RAD51/DMC1, and a failure to complete DSB repair [60, 61]. Unexpectedly, this study demonstrated that cKO mouse oocytes were blocked in the early diplotene stage, mainly due to the incomplete deficiency of the MSH5 protein in *Srsf1* cKO oocytes. However, we found that the number of RPA1 and DMC1 foci on chromosomes was significantly increased in *Srsf1* cKO oocytes relative to control oocytes (Fig. 6c, e) and that a large number of DSBs were not repaired correctly in *Srsf1* cKO oocytes (Fig. 6b). These phenotypes are similar to those of MSH5-deficient oocytes [61].

C14ORF39/SIX6OS1 is the central element of the SC, defects in which cause POI [20, 62]. The loss of SRSF1 leads to the abnormal splicing of *Six6os1* pre-mRNA, and SIX6OS1 does not localize to the central element of the SC in most early pachytene oocytes in this genetic background (Fig. 7k). The observed SC defects partially explain why failure of synapsis occurs in SRSF1-deficient oocytes. Synapsis provides the basis for crossover recombination [63]. Therefore, the formation of COs in diplotene oocytes is decreased (Fig. 5b). These defects prevent the diplotene oocytes from undergoing typical meiotic arrest at the dictyate stage, which seriously impairs primordial follicle formation.

### Quality control mechanisms eliminate defective oocytes

Oocyte quality and number are important determinants of reproductive success [64]. The selective elimination of oocytes during the early stages of oogenesis influences these attributes and is normally observed in mammalian foetal ovaries [65]. Historically, oocyte elimination in mice has been attributed to three quality control mechanisms: meiotic silencing, the synapsis checkpoint, and the DNA damage checkpoint [4]. We observed increased DNA damage and accumulation of unrepaired DSBs, as indicated by the abundances of γH2AX and RPA1, respectively, suggesting less effective DNA damage repair despite an increased number of DMC1 foci (Fig. 6a–f). Additionally, we found that low expression of the MSH5 protein led to defects in HR and synapsis (Fig. 7g, i, j). Defective oocytes with both persistent DNA damage and chromosome asynapsis may be eliminated by the combined effects of the synapsis and DNA damage checkpoints. This study showed that defective oocytes were eliminated by this mechanism in the ovaries of newborn, 5 dpp, and adult mice (Figs. 2v and 3e, o). Interestingly, oocytes in meiotic arrest were detected in 5 dpp cKO mouse ovaries (Fig. 4g). The primordial follicle pool is depleted in 6 dpp *Msh5*^–/–^ and *Six6os1*^−/−^ mouse ovaries [62, 66]. Therefore, this is a significant reason that SRSF1 deficiency impairs quality control mechanisms. However, we could not illuminate the specific mechanism due to the premature deletion of SRSF1. Next, we will further develop this exciting work in other mouse models. Nevertheless, it is clear that SRSF1 is indispensable for oocyte survival.

In summary, our study provides an important theoretical basis for understanding the mechanisms of posttranscriptional regulation of oocyte meiosis by pre-mRNA splicing. Additionally, the discovery of the AS of POI-related genes in a mouse model provides the molecular basis for the clinical diagnosis and treatment of POI and the generation of eggs from induced stem cells.

## Conclusions

**Fig. 8:**
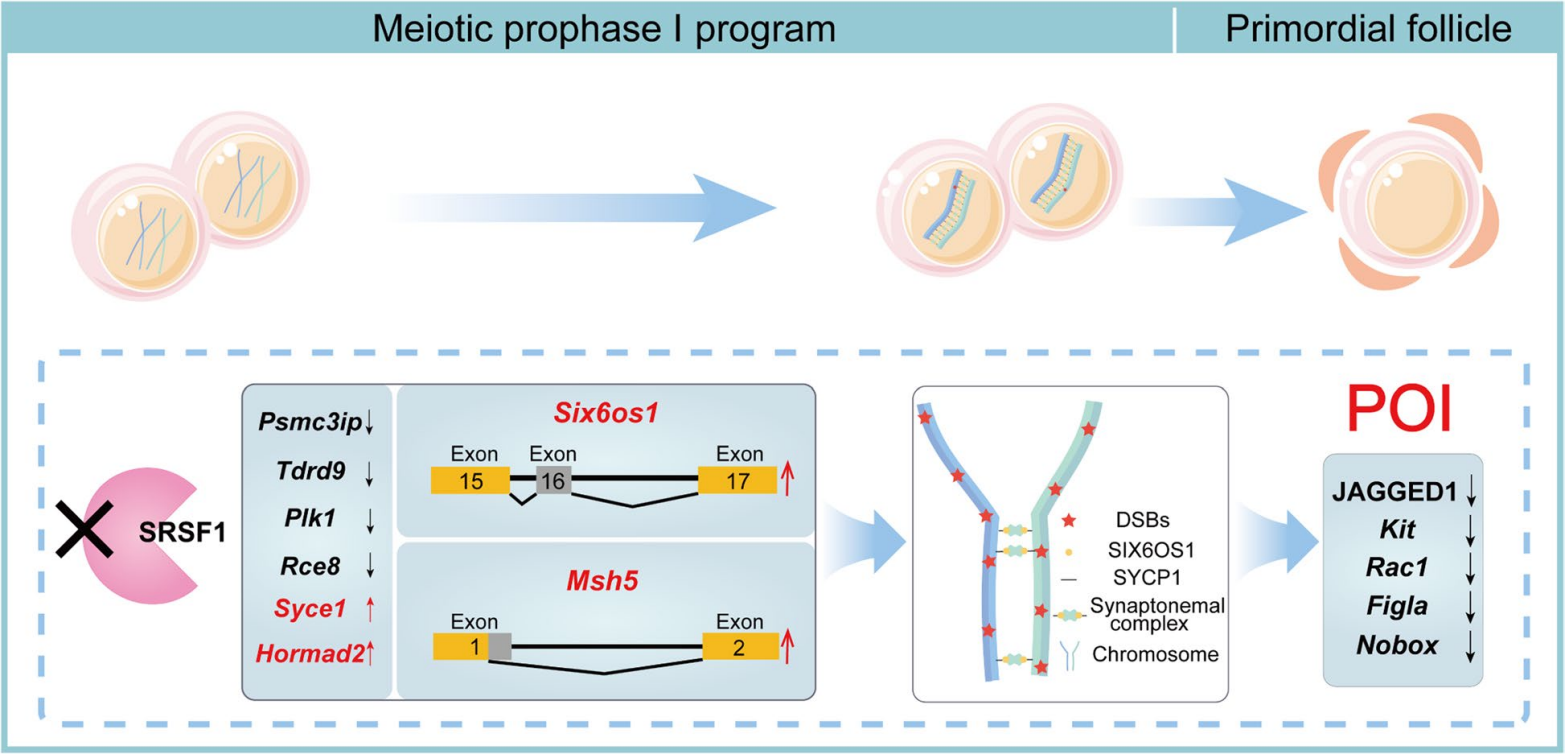
A schematic illustration of the molecular mechanisms by which SRSF1 regulates primordial follicle formation and number determination during meiotic prophase I

This study demonstrates that SRSF1 plays a critical role in posttranscriptional regulation by specifically regulating the splicing of the POI-related genes *Msh5* and *Six6os1* during meiotic prophase I in mouse oocytes. This SRSF1-mediated posttranscriptional regulation is essential for primordial follicle formation and number determination (Fig. 8).

## Methods

### Mouse strains

C57BL/6N and ICR mice were purchased from Beijing Vital River Laboratory Animal Technology Co., Ltd. *Srsf1*^*Fl/Fl*^ mice were generated in the laboratory of Prof. Xiangdong Fu (University of California, San Diego, USA) and were kindly provided by Prof. Yuanchao Xue (Institute of Biophysics, Chinese Academy of Sciences, Beijing, China) [29]. *Stra8-GFPCre* mice were kindly provided by Prof. Minghan Tong (Shanghai Institute of Biochemistry and Cell Biology, Chinese Academy of Sciences, Shanghai, China) [35, 36]. To generate *Srsf1* cKO mice, *Stra8-GFPCre* mice were crossed with *Srsf1*^*Fl/Fl*^ mice. The primers used for PCR to genotype *Srsf1*^*Fl/Fl*^ and *Stra8-GFPCre* mice are shown in Additional file 6: Table 3. All mice were bred and housed under specific pathogen-free conditions with controlled temperature (22 ± 1 °C) and exposed to a constant 12-h light–dark cycle in the animal facilities of China Agricultural University. All experiments were conducted according to the guidelines and with the approval of the Institutional Animal Care and Use Committee of China Agricultural University (No. AW80401202-3–6).

### Fertility test

For 3 months, two 6-week-old female mice were caged with one 8-week-old C57BL/6N male wild-type mouse. The number of pups from each female was recorded each day, and the date of parturition was recorded. These results were used to analyse the reproductive curve.

### Immunostaining and histologic analysis

Mouse ovaries were fixed with 4% paraformaldehyde (P6148-500G, Sigma–Aldrich, Missouri, USA) in PBS (pH 7.4) at 4 °C overnight, dehydrated in graded ethanol solutions, vitrified with xylene, and embedded in paraffin. Tissue sections were cut at a 5-μm thickness for immunostaining and histologic analysis or an 8-μm thickness for counting the follicles in adult ovaries. For histological analysis, sections were dewaxed in xylene and rehydrated in graded ethanol solution and then stained with haematoxylin. After sealing the slides with neutral resin, a Ventana DP200 system was used for imaging. For immunofluorescence analysis, antigen retrieval was performed by microwaving the sections with sodium citrate buffer (pH 6.0). After blocking with 10% goat serum at room temperature for 1 h, the sections were incubated with primary antibodies in 5% goat serum (Additional file 7: Table 4) overnight at 4 °C. After washing with PBS, the sections were incubated with secondary antibodies (Additional file 7: Table 4) at room temperature in the dark for 1 h. The slides were mounted in antifade mounting medium with DAPI (P0131, Beyotime, Shanghai, China). Photos were taken with a Nikon A1 laser scanning confocal microscope and a Zeiss OPTOME fluorescence microscope. For immunohistochemistry analysis, the same protocol that was applied for immunofluorescence analysis was followed up to the heat-mediated antigen retrieval step, and subsequent operations followed the IHC kit instructions (SP-9000, ZSGB-BIO, Beijing, China). Colour visualization in the sections was achieved with a DAB horseradish peroxidase colour development kit (ZLI-9017, ZSGB-BIO, Beijing, China). Imaging was performed according to the histological analysis protocol.

### Quantification of follicles and oocytes

Serial 8-μm sections were cut from paraffin-embedded adult ovaries. All follicles or oocytes in VASA immunostained ovarian sections were counted. Follicle types were classified according to the following structural characteristics. As described previously [67], the following follicle types were distinguished: primordial follicles (type 1 and type 2), primary follicles (type 3), secondary follicles (type 4 and type 5), and antral follicles (types 6 − 8).

Serial 5-μm sections were obtained from paraffin-embedded ovaries from 5 dpp and newborn mice. The sections were stained with haematoxylin, and the following categories of oocytes and follicles were counted in every fifth section: primordial follicles (oocytes approximately 20 μm in diameter surrounded by 3 − 5 flat pregranulosa cells each); oocytes in cysts (two or more oocytes with shared cytoplasm); and naked oocytes (oocytes without any granulosa cells). As described previously [68], the sum of these counts was multiplied by five to estimate the total number of oocytes and follicles in each ovary.

### Oocyte surface spreading

Oocyte purification was performed as described previously [69]. In brief, mouse ovaries were isolated and digested with 90 μl of TrypLE (12,604,021, Thermo, New York, US) at 37 °C for 10 min under constant shock. Digestion was terminated by adding 10 μl foetal bovine serum (C0235, Beyotime, Shanghai, China). After centrifugation at 1000 rpm for 1 min, the supernatant was discarded, and the cells were treated with 80 μl hypotonic buffer (30 mM Tris–HCl, 17 mM trisodium citrate dihydrate, 50 mM sucrose, 5 mM EDTA, 0.5 mM DTT, pH 8.2) containing proteinase inhibitor (1:100, P1005, Beyotime, Shanghai, China) for 30 min. Slides were pretreated with 20 μl of fixation buffer (1% paraformaldehyde, pH 9.2 with 50 mM boric acid) containing 0.15% Triton X-100 (T9284, Sigma, Missouri, USA) applied evenly on slides in advance. Then, 20 μl aliquots of the cell suspension were dripped onto the slides, which were incubated at 37 °C for 4 h in a humidified box. The samples were left to dry at room temperature. Then, immunofluorescence staining was performed according to the protocol described above.

### RT–PCR and RT–qPCR

Total RNA was extracted by using RNAiso Plus (9109, Takara, Kusatsu, Japan) and a Direct-zol RNA MicroPrep kit (R2060, Zymo Research, California, USA), and the concentration was measured with a Nano-300 ultramicro spectrophotometer (Allsheng, Hangzhou, China). cDNA was obtained according to the instructions of the TIANScript II RT kit (KR107, TIANGEN, Beijing, China). The expression of transcripts of the target gene was measured by using a Light Cycle® 96 instrument (Roche) with Hieff UNICON SYBR green master mix (11198ES08, Yeasen, Shanghai, China). AS analyses were performed on a RePure-A PCR instrument (BIO-GENER, Hangzhou,China). Primers were synthesized by Sangon Biotech (Additional file 6: Table 3). The expression level of *Gapdh* or *Vasa* was used as the control, and this value was set as 1. The relative transcript expression levels of other samples were obtained by comparing them with the control results.

### RNA stability assay

One half of an ovary was treated with 50 μg/mL actinomycin D (ActD, SBR00013, Sigma–Aldrich, Missouri, USA) for transcription inhibition. Samples were collected at 0 and 120 min after transcription inhibition. The RNA stabilities of *Msh5* and *Six6os1* were determined by analysing total RNA without gDNA.

### RNA-seq

Total RNA was extracted from mouse ovaries according to the above protocol at 16.5 dpc. Briefly, mRNA was purified from total RNA using poly-T oligo-attached magnetic beads. After fragmentation, we established a transcriptome sequencing library and assessed library quality on an Agilent Bioanalyzer 2100 system. The clustering of the index-coded samples was performed on a cBot Cluster Generation System using a TruSeq PE Cluster kit v3-cBot-HS (Illumina) according to the manufacturer’s instructions. After cluster generation, the library preparations were sequenced on the Illumina NovaSeq platform, and 150 bp paired-end reads were generated. After quality control, all the downstream analyses were performed on clean, high-quality data. The index of the reference genome was built, and paired-end clean reads were aligned to the reference genome using HISAT2 software (version 2.0.5). FeatureCounts (version 1.5.0) was used to count the number of reads mapped to each gene. Then, the fragments per kilobase million (FPKM) value of each gene was calculated based on the length of the gene and the read count mapped to this gene. Differential expression analysis of cKO/Ctrl ovaries (three biological replicates per condition) was performed using the DESeq2 R package (version 1.20.0). Genes with a *P* value < 0.05 identified by DESeq2 were considered differentially expressed.

### AS analysis

rMATS software (version 3.2.5) was used to analyse the AS events in cKO mouse ovaries based on RNA-seq data. Five types of AS events (SE, RI, MXE, A5SS, and A3SS) were revealed by rMATS software. Our threshold for screening differentially significant AS events was a false discovery rate (FDR) less than 0.05. Splicing events with an FDR less than 0.05 and an inclusion-level difference with a significance of at least 5% (0.05) were considered statistically significant. The IGV (Version 2.10.2) was used to visualize and confirm AS events based on RNA-seq data.

### GO term enrichment analysis

The GO enrichment analysis of differentially expressed genes and AS genes was implemented with the clusterProfiler R package (version 3.4.4), in which gene length bias was corrected. Mouse genome data (GRCm38/mm10) were used as the reference, and Benjamini–Hochberg multiple tests were applied to adjust multiple testing. GO terms with corrected *P* values of less than 0.05 were considered significantly enriched by differentially expressed genes and AS genes.

### Western blotting

Total protein was extracted with cell lysis buffer (P0013, Beyotime, Shanghai, China) containing PMSF (1:100, ST506, Beyotime, Shanghai, China) and a protease inhibitor cocktail (1:100, P1005, Beyotime, Shanghai, China). A BCA protein assay kit (CW0014S, CWBiotech, Beijing, China) was used to measure the protein concentration. The protein lysates were electrophoretically separated on sodium dodecyl sulfate–polyacrylamide gels and electrically transferred to polyvinylidene fluoride membranes (IPVH00010, Millipore, Ireland). The membranes were blocked in 5% skimmed milk for 1 h and incubated with the primary antibodies (Additional file 7: Table 4) for one night at 4 °C. Then, the membranes were incubated with secondary antibodies (Additional file 7: Table 4) at room temperature for 1 h. Proteins were visualized using the Tanon 5200 chemiluminescence imaging system following incubation with BeyoECL Plus (P0018S, Beyotime, Shanghai, China).

### RNA immunoprecipitation (RIP) and RIP–qPCR

As described previously [70], RIP was performed using 16.5 dpc mouse ovaries. The ovaries were lysed in cell lysis buffer (P0013, Beyotime, Shanghai, China) containing PMSF (1:100, ST506, Beyotime, Shanghai, China), a proteinase inhibitor cocktail (1:100, P1005, Beyotime, Shanghai, China), DTT (1:50, ST041-2 ml, Beyotime, Shanghai, China), and an RNase inhibitor (1:20, R0102-10 kU, Beyotime, Shanghai, China). After incubation on ice for 20 min, the lysate was added to 20 μl of protein A agarose (P2051-2 ml, Beyotime, Shanghai, China) for preclearing at 4 °C for 1 h. Two micrograms of an SRSF1 antibody (sc-33652, Santa Cruz Biotechnology, California, USA) and a normal mouse IgG (sc-3879, Santa Cruz Biotechnology, California, USA) were added to the lysate, followed by overnight incubation at 4 °C. The next day, 60 μl of protein A agarose was added to the lysate followed by incubation at 4 °C for 4 h. The agarose complexes containing antibodies, target proteins, and RNA were washed for 5 min at 4 °C, which was repeated 3 times. Protein-bound RNA was then extracted using RNAiso Plus and the Direct-zol RNA MicroPrep kit. RIP–qPCR was performed according to the above RT–qPCR protocol.

### Statistical analysis

GraphPad Prism software (version 9.0.0) was used for the statistical analyses, and the values and error bars shown represent the mean ± SEM. Significant differences between the two groups were analysed using Student’s *t* test. Statistical significance was considered as follows: detailed *P* value *P* ≥ 0.05; *P < 0.05; **P < 0.01; ***P < 0.001; ****P < 0.0001).

## Supplementary Information

**Additional file 1: Fig. S1.** The expression pattern of SRSF1 in ovarian development and ageing. a The expression of* Srsf1 *during ovarian development and ageing. Real-time qPCR data were normalized to *Gapdh*. b Western blotting of SRSF1 expression in 17.5 dpc Ctrl and cKO ovaries. c The relative protein expression level of SRSF1 is shown in ovarian development and ageing. ACTB served as a loading control. The value in 12 M ovaries was set as 1.0. d Immunostaining was performed using VASA and SRSF1 antibodies from NB, 6 W, and 10 M ovaries. DNA was stained with DAPI. Scale bar, 10 μm. NB, newborn; W, week; M, month.

**Additional file 2: Table 1. **Differential genes were analysed in this study.

**Additional file 3: Table 2.** AS events were analysed in cKO and Ctrl ovaries.

**Additional file 4: Fig. S2.** SRSF1 regulates the stability of *Msh5* and *Six6os1*. a, b Western blotting of MSH5 and SIX6OS1 expression in 17.5 dpc Ctrl and cKO ovaries. GAPDH (a) or ACTB (b) served as a loading control. c The expression of* Msh5 *in 16.5 dpc cKO ovaries after ActD treatment at different times. *n*=4.

**Additional file 5: Fig. S3. **Various genes were visually analysed using IGV.

**Additional file 6: Table 3.** Primer sequences were used in this study.

**Additional file 7: Table 4.** Antibodies were used in this study.

**Additional file 8.** Uncropped gels/blots.

**Additional file 9.** Individual data values.

## Data Availability

All data generated or analysed during this study are included in this published article, its supplementary information files and publicly available repositories. The uncropped gels/blots are provided in Additional file 8. The individual data values for Figs. 2, 3, 4, 5, 6, and 7, as well as Additional file 1: Fig. S1 and Additional file 4: Fig. S2, are provided in Additional file 9. The RNA-seq data were deposited in GEO (https://www.ncbi.nlm.nih.gov/geo/) under accession number GSE198205.

## Abbreviations

AS: Alternative splicing
SRSF1/SF2/ASF: Serine/arginine-rich splicing factor 1
cKO: Conditional knockout
POI: Primary ovarian insufficiency
LHX8: LIM homeobox protein 8
NOBOX: Newborn ovary homeobox protein
SOHLH: Spermatogenesis- and oogenesis-specific basic helix-loop-helix-containing protein
FIGLA: Factor in the germline alpha
JAG1: Protein jagged-1
RAC1: Ras-related C3 botulinum toxin substrate 1
STRA8: Stimulated by retinoic acid gene 8 protein
COs: DNA crossovers
DSB: SPO11-linked DNA double-strand break
NGS: Next-generation sequencing
SC: Synaptonemal complex
STAG3: Stromal antigen 3
SMC: Structural maintenance of chromosomes
SYCE: Synaptonemal complex central element
C14ORF39/SIX6OS1: Six6 opposite strand transcript 1 homologue
TEX12: Testis-expressed protein 12
MEIOB: Meiosis-specific with OB domain-containing protein
MCM: Mini-chromosome maintenance
MSH: MutS protein homologue
SYCP: Synaptonemal complex protein
dpc: Days post-coitus
dpp: Days post-partum
RT: Reverse transcription
qPCR: Quantitative real-time PCR
GM130: 130 KDa cis-Golgi matrix protein
Ctrl: Control
GO: Gene Ontology
TDRD9: Tudor domain-containing protein 9
PLK1: Polo-like kinase 1
HORMAD2: HORMA domain-containing protein 2
MSY2: Germ cell-specific Y-box-binding protein
MLH1: MutL homologue 1
HR: Homologous recombination
γH2AX: Phosphorylated histone H2AX
RPA: Replication protein A
FDR: False discovery rate calculated from the *P* value
SEs: Skipped exons
A5SSs: Alternative 5′ splice sites
A3SSs: Alternative 3′ splice sites
MXEs: Mutually exclusive exons
RIs: Retained introns
IGV: Integrative Genomics Viewer
RIP: RNA immunoprecipitation
SPIDR: Scaffolding protein involved in DNA repair
IRF7: Interferon regulatory factor 7
IL27RA: Interleukin-27 receptor subunit alpha
MYO1B: Myosin IB
ICR: Institute of Cancer Research
GAPDH: Glyceraldehyde-3-phosphate dehydrogenase
ActD: Actinomycin D
