# Supplementary figures and images for: SRSF1 regulates primordial follicle formation and number determination during meiotic prophase I
Source: BMC Biol. 2023 Mar 8;21:49. doi: 10.1186/s12915-023-01549-7 (PMC9993595; doi:10.1186/s12915-023-01549-7)

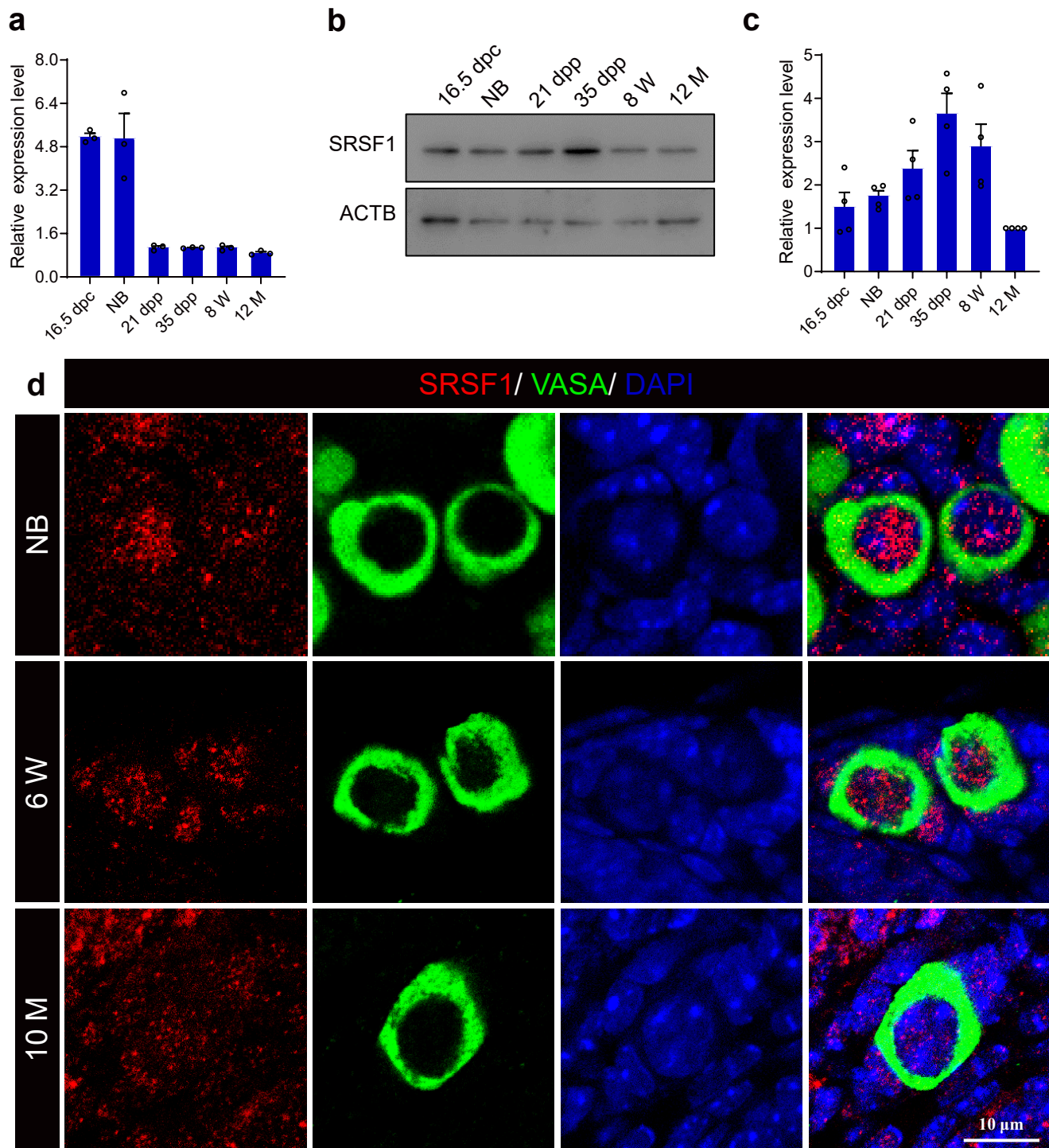

Supplement: Supplementary file 1 — Additional file 1: Fig. S1. The expression pattern of SRSF1 in ovarian development and ageing. a The expression of Srsf1 during ovarian development and ageing. Real-time qPCR data were normalized to Gapdh. b Western blotting of SRSF1 expression in 17.5 dpc Ctrl and cKO ovaries. c The relative protein expression level of SRSF1 is shown in ovarian development and ageing. ACTB served as a loading control. The value in 12 M ovaries was set as 1.0. d Immunostaining was performed using VASA and SRSF1 antibodies from NB, 6 W, and 10 M ovaries. DNA was stained with DAPI. Scale bar, 10 μm. NB, newborn; W, week; M, month. [file 12915_2023_1549_MOESM1_ESM.pdf]

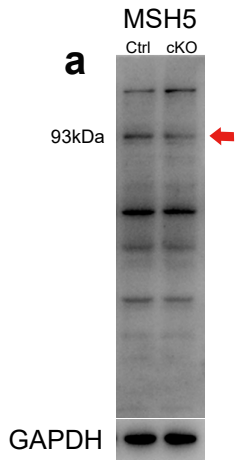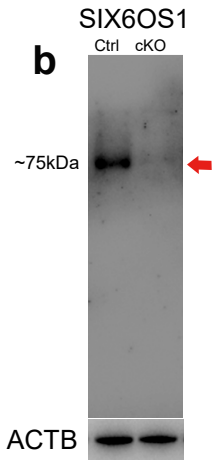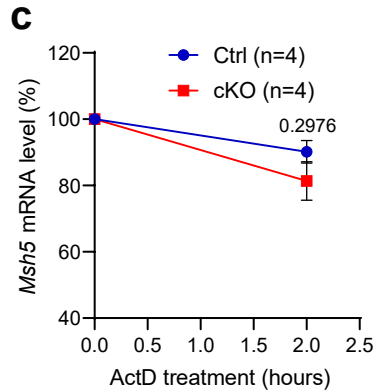

Supplement: Supplementary file 4 — Additional file 4: Fig. S2. SRSF1 regulates the stability of Msh5 and Six6os1. a, b Western blotting of MSH5 and SIX6OS1 expression in 17.5 dpc Ctrl and cKO ovaries. GAPDH (a) or ACTB (b) served as a loading control. c The expression of Msh5 in 16.5 dpc cKO ovaries after ActD treatment at different times. n=4. [file 12915_2023_1549_MOESM4_ESM.pdf]

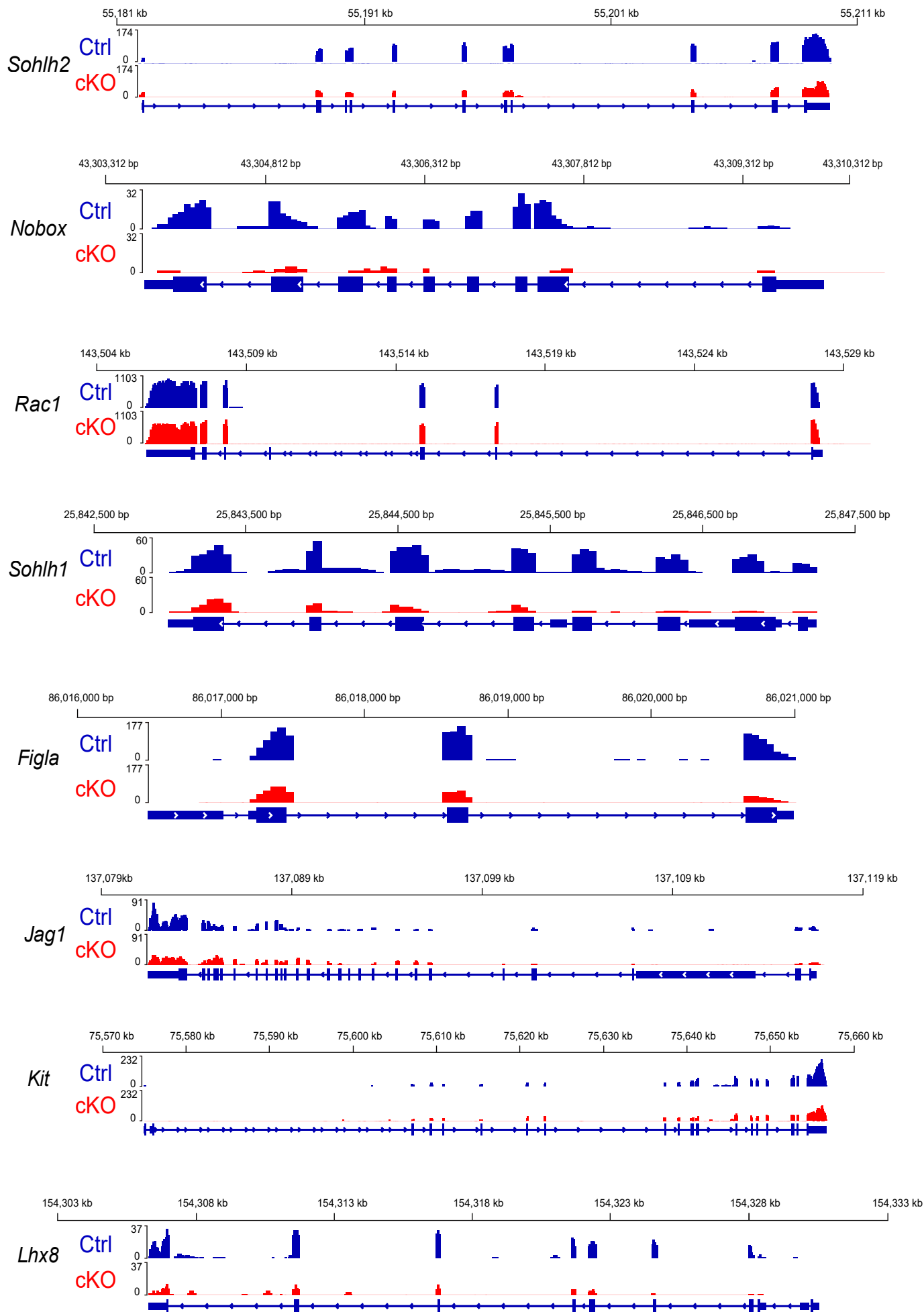

Supplement: Supplementary file 5 — Additional file 5: Fig. S3. Various genes were visually analysed using IGV. [file 12915_2023_1549_MOESM5_ESM.pdf]

Fig. 2c

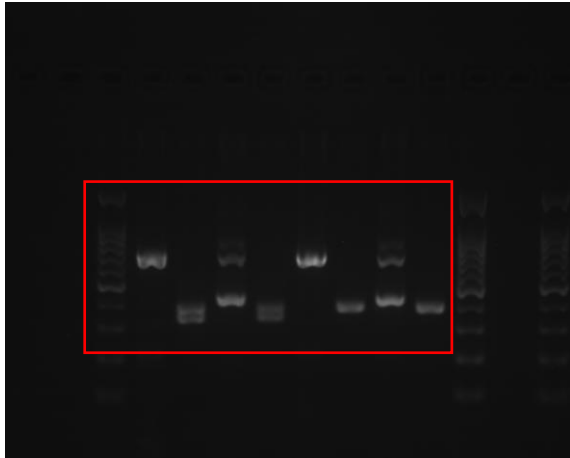

Fig. 7d

*Six6os1*

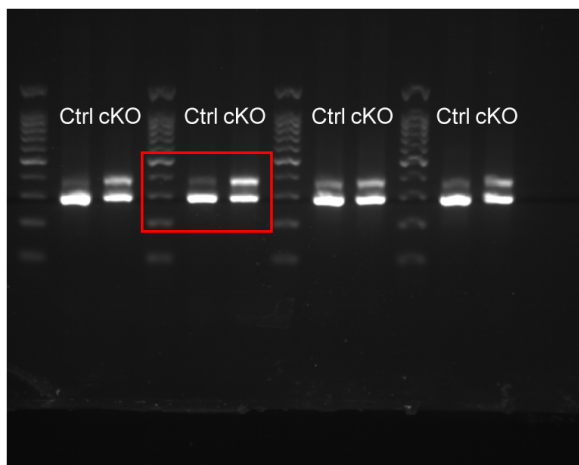

*Msh5*

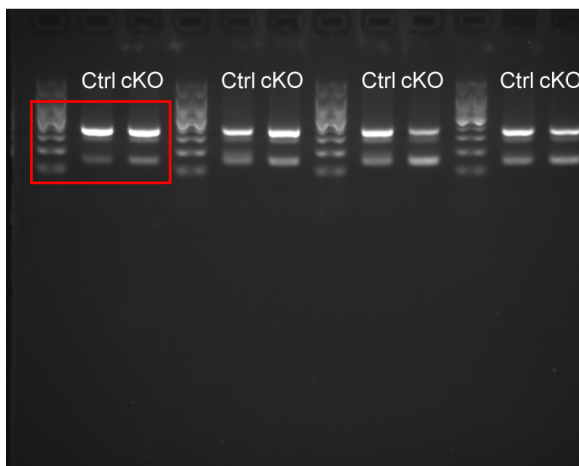

Fig. 7g

GAPDH

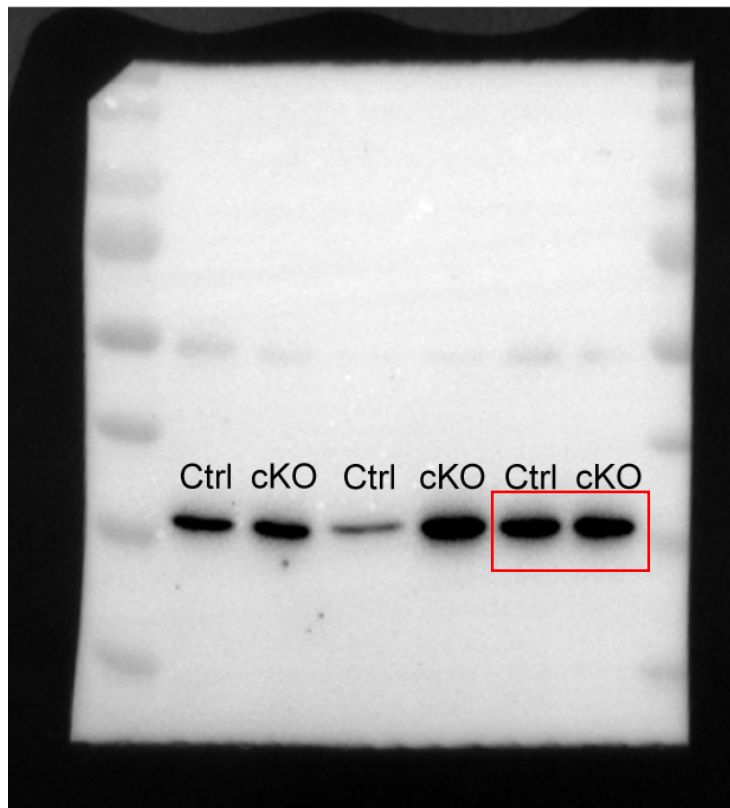

MSH5

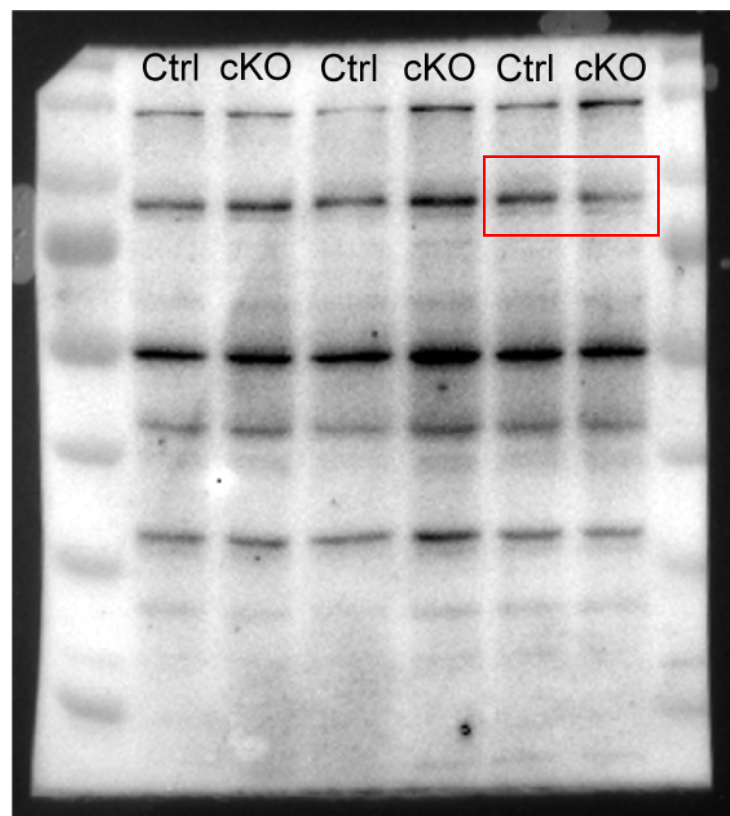

Fig. 7h

ACTB

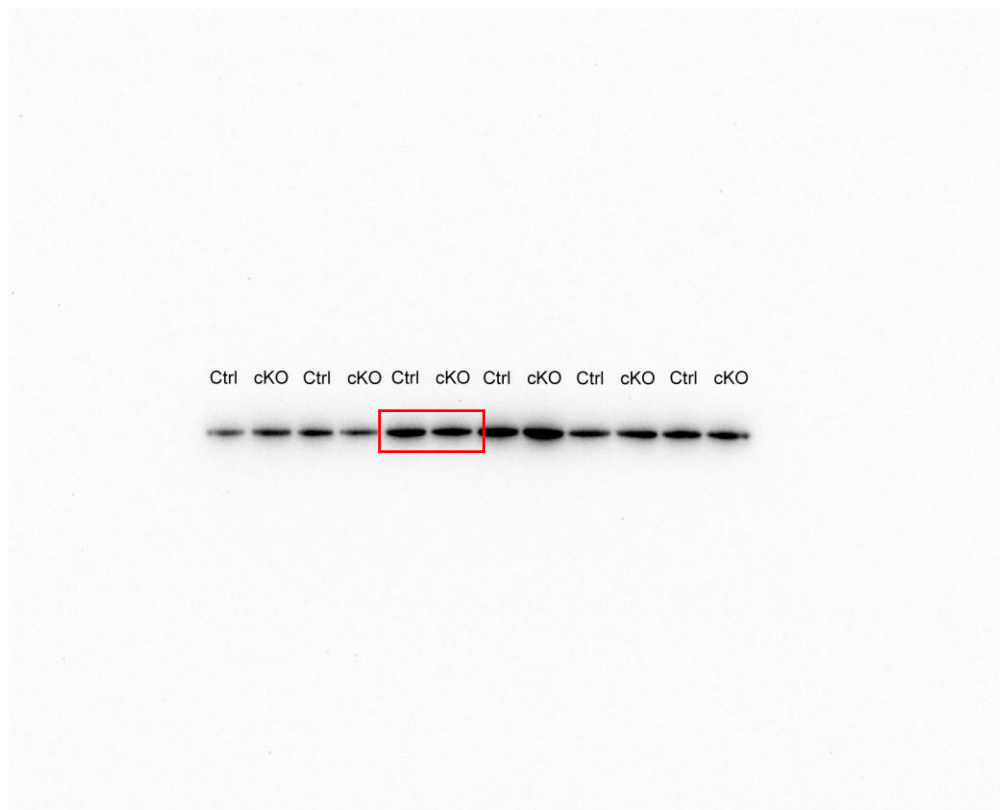

SIX6OS1

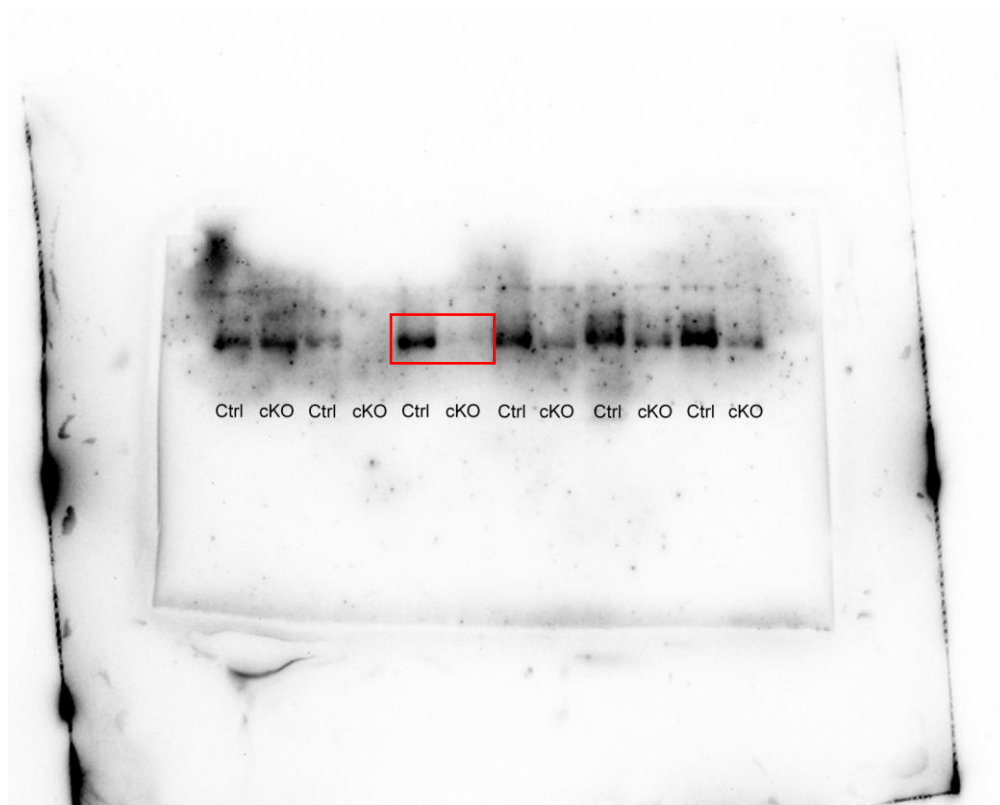

Fig. S1b

SRSF1

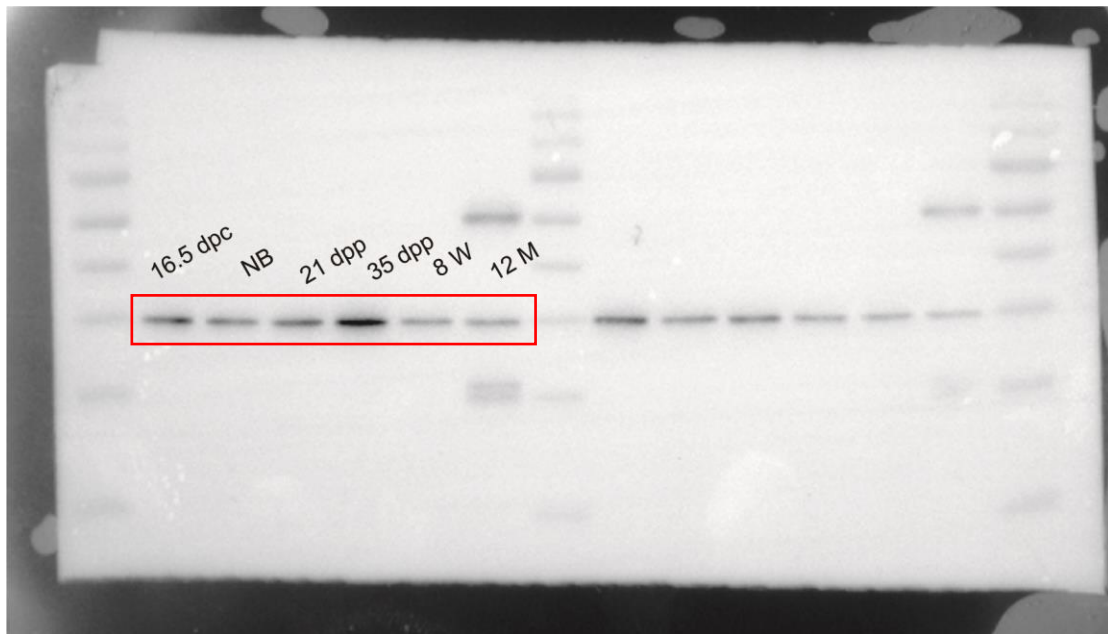

ACTB

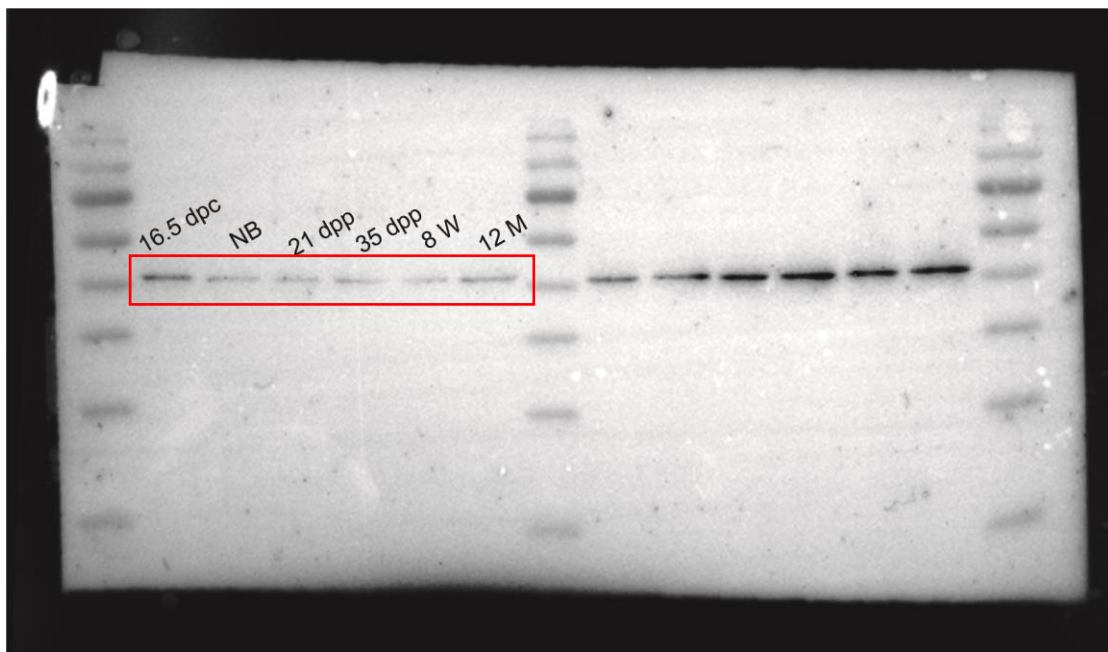

Supplement: Supplementary file 8 — Additional file 8. Uncropped gels/blots. [file 12915_2023_1549_MOESM8_ESM.pdf]
