# Supplementary material for: SRSF1 regulates primordial follicle formation and number determination during meiotic prophase I
Source: BMC Biol. 2023 Mar 8;21:49. doi: 10.1186/s12915-023-01549-7 (PMC9993595; doi:10.1186/s12915-023-01549-7)
Supplement: Supplementary file 2 — Additional file 2: Table 1. Differential genes were analysed in this study. [file 12915_2023_1549_MOESM2_ESM.pdf]

Additional file 2: Table 1. Differential genes are analyzed in this study.

gene_id	cKO1	cKO2	cKO3	Control4	Control5	Control6	cKO	Ctrl1	log2Fold Change	pvalue	padj	gene_name	gene_chr
ENSMUSG00000021032	141.618	46.1415	95.0332	14.6803	9.70494	9.87139	94.2642	11.4189	3.04988	2.72E-09	5.64E-05	*Ngb*	12
ENSMUSG00000018379	2914.88	3339.14	3295.98	7204.88	6292.68	5650.03	3183.33	6382.53	-1.00353	3.48E-08	0.00032	*Srsf1*	11
ENSMUSG00000052187	993.363	239.183	264.975	95.422	61.1411	104.098	499.174	86.8871	2.52212	4.59E-08	0.00032	*Hbb-y*	7
ENSMUSG00000085601	6188.4	2500.12	4700.23	664.808	1241.26	1697.88	4462.92	1201.32	1.89303	8.43E-08	0.00044	*Gm4969*	7
ENSMUSG00000021182	253.69	297.566	242.614	524.297	614.323	571.643	264.623	570.088	-1.10642	1.15E-07	0.00048	*Ccdc88c*	12
ENSMUSG00000044303	199.691	171.383	297.398	59.7698	44.6427	95.1243	222.824	66.5123	1.73986	7.36E-07	0.00254	*Cdkn2a*	4
ENSMUSG00000095478	511.455	604.548	408.084	169.872	267.856	155.25	508.029	197.659	1.3627	9.00E-07	0.00267	*Gm9824*	10
ENSMUSG00000108315	10.1883	0.94166	5.59019	45.0895	307.647	373.318	5.5734	242.018	-5.44639	1.14E-06	0.00295	*Gm44601*	7
ENSMUSG00000041828	17.3202	7.53331	14.5345	38.7979	71.8165	109.483	13.1293	73.3657	-2.49206	2.55E-06	0.00588	*Abca8a*	11
ENSMUSG00000056753	269.991	150.666	286.218	61.867	87.3444	96.9191	235.625	82.0435	1.51833	3.57E-06	0.0074	*C330011M18Rik*	8
ENSMUSG00000050994	3.0565	2.82499	2.23608	15.7289	39.7902	46.6648	2.70586	34.0613	-3.65184	4.08E-06	0.00769	*Adgb*	10
ENSMUSG00000083773	14.2637	16.9499	7.82627	16.7775	1583.85	1703.26	13.0133	1101.3	-6.40151	5.05E-06	0.00872	*Gm13394*	2
ENSMUSG00000112206	31.5839	16.9499	70.4364	3.14578	0.97049	5.3844	39.6567	3.16689	3.62843	5.67E-06	0.0089	*Gm30228*	10
ENSMUSG00000029503	118.185	39.5499	110.686	10.4859	21.3509	22.435	89.4735	18.0906	2.29764	6.01E-06	0.0089	*P2rx2*	5
ENSMUSG00000107880	0	0	0	4.19437	16.4984	43.0752	0	21.256	-6.82332	6.75E-06	0.00933	*Gm44113*	6
ENSMUSG00000073627	424.854	122.416	288.454	66.0614	77.6395	101.406	278.575	81.7023	1.76728	1.00E-05	0.01297	*C130036L24Rik*	1

ENSMUSG00000067702	731.523	635.623	1640.16	248.517	184.394	480.109	1002.44	304.34	1.71855	1.14E-05	0.01391	*Tuba3a*	6
ENSMUSG00000090256	67.243	7.53331	44.7215	3.14578	3.88198	1.7948	39.8326	2.94085	3.76561	1.21E-05	0.01395	*Alms1-ps1*	6
ENSMUSG00000025241	3136.99	1683.69	2246.14	1005.6	1148.09	1205.21	2355.61	1119.63	1.07286	1.33E-05	0.01453	*Fyco1*	9
ENSMUSG00000094004	1.01883	0.94166	8.9443	17.8261	35.9083	78.9712	3.63493	44.2352	-3.65092	1.42E-05	0.01469	*Gm5128*	X
ENSMUSG00000039629	897.593	341.824	972.693	139.463	237.771	331.14	737.37	236.125	1.64122	1.92E-05	0.01898	*Strip2*	6
ENSMUSG00000056300	314.82	82.8664	320.877	29.3606	72.787	73.5868	239.521	58.5781	2.02786	2.06E-05	0.01917	*Zfp981*	4
ENSMUSG00000002108	138.561	91.3414	207.955	49.2839	49.4952	51.1518	145.953	49.9769	1.54326	2.13E-05	0.01917	*Nr1h3*	2
ENSMUSG00000022838	311.763	164.791	447.215	56.624	98.9904	138.2	307.923	97.938	1.64881	2.99E-05	0.02447	*Eaf2*	16
ENSMUSG00000097667	47.8852	20.7166	59.256	10.4859	3.88198	5.3844	42.6193	6.5841	2.7026	3.13E-05	0.02447	*Gm26764*	12
ENSMUSG00000037747	627.602	205.283	673.059	101.714	107.725	234.221	501.981	147.887	1.76088	3.15E-05	0.02447	*Phyhipl*	10
ENSMUSG00000029564	5.09417	0	1.11804	8.38875	41.7312	52.9466	2.07074	34.3555	-4.06479	3.27E-05	0.02447	*4930519G04Rik*	5
ENSMUSG00000020469	62.1489	40.4915	64.8462	14.6803	10.6754	17.0506	55.8289	14.1354	1.97711	3.37E-05	0.02447	*Myl7*	11
ENSMUSG00000093675	0	0	0	1.04859	15.5279	42.1778	0	19.5848	-6.70485	3.42E-05	0.02447	*Gm20618*	6
ENSMUSG00000116124	0	1.88333	1.11804	7.34015	26.2033	46.6648	1.00046	26.7361	-4.72424	3.86E-05	0.02671	*Gm49420*	15
ENSMUSG00000032484	233.313	106.408	115.158	1011.89	293.089	302.424	151.626	535.802	-1.82053	5.08E-05	0.03397	*Ngp*	9
ENSMUSG00000040570	853.783	597.956	1175.06	348.133	346.466	501.646	875.599	398.749	1.13362	5.59E-05	0.03597	*Rundc3b*	5
ENSMUSG00000064225	63.1677	46.1415	43.6035	224.399	255.24	62.818	50.9709	180.819	-1.82514	5.91E-05	0.03597	*Paqr9*	9
ENSMUSG00000098703	81.5067	16.9499	42.4854	8.38875	3.88198	8.97399	46.9807	7.08157	2.72737	5.96E-05	0.03597	*Gm27527*	5

ENSMUSG00000026173	128.373	41.4332	115.158	7.34015	23.2919	31.409	94.9881	20.6803	2.18909	6.07E-05	0.03597	*Plcd4*	1
ENSMUSG00000052861	97.8081	20.7166	58.138	11.5345	7.76395	13.461	58.8875	10.9198	2.42736	6.51E-05	0.0375	*Dnah6*	6
ENSMUSG00000051159	636.771	409.624	922.381	133.171	248.446	346.396	656.259	242.671	1.43332	6.74E-05	0.0378	*Cited1*	X
ENSMUSG00000032591	17.3202	17.8916	30.187	116.394	95.1084	39.4856	21.7996	83.6626	-1.94371	7.10E-05	0.03833	*Mst1*	9
ENSMUSG00000020461	177.277	50.8498	174.414	22.0205	30.0853	52.0492	134.18	34.7183	1.94379	7.28E-05	0.03833	*Clhc1*	11
ENSMUSG00000032496	151.806	112.058	52.5478	635.447	312.499	159.737	105.471	369.228	-1.80519	7.65E-05	0.03833	*Ltf*	9
ENSMUSG00000026643	5233.75	2972.83	6466.73	2188.41	2054.54	2685.02	4891.1	2309.32	1.08252	7.76E-05	0.03833	*Nmt2*	2
ENSMUSG00000109032	2896.54	1208.15	2414.96	486.547	763.779	1224.05	2173.22	824.793	1.39718	7.76E-05	0.03833	*Gm7972*	9
ENSMUSG00000105034	0	0	0	4.19437	14.5574	21.5376	0	13.4298	-6.1607	8.02E-05	0.03869	*Gm43348*	3
ENSMUSG00000105873	140.599	46.1415	190.066	16.7775	40.7607	36.7934	125.602	31.4439	1.99204	8.40E-05	0.0396	*Gm43708*	5
ENSMUSG00000071424	4.07534	4.70832	5.59019	23.069	18.4394	61.9206	4.79128	34.4763	-2.85481	9.15E-05	0.04218	*Grid2*	6
ENSMUSG00000023826	176.258	133.716	155.407	63.9642	74.728	76.279	155.127	71.6571	1.11225	9.60E-05	0.0433	*Prkn*	17
ENSMUSG00000073421	406.515	266.491	458.396	67.11	104.813	223.452	377.134	131.792	1.51337	0.0001	0.04365	*H2-Ab1*	17
ENSMUSG00000023045	21.3955	37.6665	49.1937	242.225	120.341	55.6388	36.0852	139.402	-1.94995	0.0001	0.04365	*Soat2*	15
ENSMUSG00000041992	1270.49	514.148	1009.59	358.619	345.496	498.954	931.408	401.023	1.21498	0.00011	0.04606	*Rapgef5*	12
ENSMUSG00000039956	64.1865	77.2164	68.2003	301.995	255.24	82.5607	69.8678	213.265	-1.60794	0.00011	0.04633	*Mrap*	16
ENSMUSG00000031383	380.025	371.015	566.845	156.24	175.659	273.707	439.295	201.869	1.11934	0.00011	0.04633	*Dusp9*	X
ENSMUSG00000037139	19.3578	15.0666	10.0623	10.4859	104.813	166.019	14.8289	93.7727	-2.65997	0.00012	0.04941	*Myom3*	4

ENSMUSG00000068048	78.4502	95.108	152.053	11.5345	29.1148	49.357	108.537	30.0021	1.84518	0.00013	0.05019	*Rhox9*	X
ENSMUSG00000102596	0	0	0	1.04859	19.4099	23.3324	0	14.597	-6.28042	0.00013	0.05062	*Gm20203*	1
ENSMUSG00000022026	41.7722	21.6583	8.9443	156.24	83.4625	58.331	24.1249	99.3446	-2.0352	0.00013	0.05062	*Olfm4*	14
ENSMUSG00000115548	0	0.94166	2.23608	2.09719	38.8198	38.5882	1.05925	26.5017	-4.68193	0.00015	0.05487	*5430440P10Rik*	14
ENSMUSG00000035439	1233.81	830.547	920.145	533.734	604.618	542.029	994.834	560.127	0.82859	0.00015	0.05503	*Haus8*	8
ENSMUSG00000021640	98.8269	19.7749	83.8529	4.19437	6.79346	22.435	67.4849	11.1409	2.58271	0.00016	0.05555	*Naip1*	13
ENSMUSG00000054994	6.113	1.88333	7.82627	6.29156	72.787	55.6388	5.2742	44.9058	-3.1078	0.00016	0.05776	*AV320801*	X
ENSMUSG00000102133	48.904	7.53331	40.2494	3.14578	3.88198	4.487	32.2289	3.83825	3.0608	0.00017	0.05799	*Gm37106*	1
ENSMUSG00000116950	70.2995	16.9499	43.6035	2.09719	6.79346	11.6662	43.6177	6.85228	2.65118	0.00018	0.06106	*AC154378.3*	17
ENSMUSG00000072944	57.0547	30.1332	72.6725	54.5268	232.919	317.679	53.2868	201.708	-1.92488	0.00019	0.0623	*Nup62cl*	X
ENSMUSG00000032056	71.3184	29.1916	72.6725	10.4859	9.70494	21.5376	57.7275	13.9095	2.04189	0.00019	0.0623	*Btg4*	9
ENSMUSG00000068417	12.226	17.8916	6.70823	56.624	72.787	28.7168	12.2753	52.7093	-2.08845	0.00019	0.0623	*Pnp2*	14
ENSMUSG00000024053	82.5255	135.6	128.574	446.701	358.112	139.994	115.566	314.936	-1.44519	0.0002	0.0623	*Emilin2*	17
ENSMUSG00000079184	3955.11	1895.57	4206.06	1253.07	1321.81	2011.07	3352.25	1528.65	1.13257	0.0002	0.0623	*Mphosph8*	14
ENSMUSG00000030760	1485.46	729.789	1758.67	555.754	469.719	750.226	1324.64	591.9	1.16155	0.00021	0.06281	*Acer3*	7
ENSMUSG00000035910	10.1883	5.64998	7.82627	17.8261	37.8493	70.8946	7.8882	42.19	-2.42705	0.00021	0.06281	*Dcdc2a*	13
ENSMUSG00000091144	266.934	138.425	279.51	71.3043	106.754	110.38	228.29	96.1463	1.2443	0.00021	0.06281	*Phf11c*	14
ENSMUSG00000067203	32.6027	15.0666	38.0133	3.14578	5.82296	4.487	28.5609	4.48525	2.66147	0.00022	0.06481	*H2-K2*	17

ENSMUSG00000046634	5.09417	16.0083	14.5345	26.2148	23.2919	188.454	11.879	79.3202	-2.74133	0.00023	0.06567	*Pkd1l1*	11
ENSMUSG00000106457	210.899	64.0331	247.086	22.0205	32.9968	81.6633	174.006	45.5602	1.92692	0.00023	0.06567	*Gm42585*	5
ENSMUSG00000109186	76.4125	14.125	48.0756	8.38875	10.6754	6.2818	46.2044	8.44866	2.45193	0.00023	0.06567	*Gm34821*	7
ENSMUSG00000030054	18.339	16.9499	26.8329	104.859	88.3149	32.3064	20.7073	75.1602	-1.86198	0.00023	0.06582	*Gp9*	6
ENSMUSG00000103685	115.128	54.6165	83.8529	30.4092	32.0263	27.8194	84.5325	30.085	1.48986	0.00024	0.0677	*Gm37074*	1
ENSMUSG00000016200	1864.47	733.556	2223.78	745.55	520.185	760.097	1607.27	675.277	1.25076	0.00026	0.07079	*Syt14*	1
ENSMUSG00000030443	665.299	309.807	569.081	240.128	178.571	287.168	514.729	235.289	1.12839	0.00027	0.07347	*Zfp583*	7
ENSMUSG00000004347	84.5632	74.3914	125.22	18.8747	23.2919	50.2544	94.725	30.807	1.61082	0.00028	0.07487	*Pde1c*	6
ENSMUSG00000056071	194.597	204.341	139.755	1046.5	316.381	250.374	179.564	537.75	-1.58106	0.00029	0.07487	*S100a9*	3
ENSMUSG00000075359	3.0565	5.64998	5.59019	4.19437	56.2886	59.2284	4.76556	39.9038	-3.06911	0.00029	0.07624	*Gm20775*	Y
ENSMUSG00000022034	4166.01	1329.63	5073.66	1196.44	1217	1738.26	3523.1	1383.9	1.34788	0.0003	0.07673	*Esco2*	14
ENSMUSG00000105703	188.484	249.541	252.677	291.509	494.952	832.787	230.234	539.749	-1.23008	0.0003	0.07673	*Gm43305*	14
ENSMUSG00000057215	23.4332	6.59165	31.3051	3.14578	0	0	20.4433	1.04859	4.35461	0.00031	0.07673	*Platr28*	7
ENSMUSG00000087512	7.13184	0.94166	6.70823	13.6317	25.2328	72.6894	4.92724	37.1846	-2.93713	0.00031	0.07673	*Gm11985*	11
ENSMUSG00000046727	186.447	97.933	169.942	40.8951	57.2591	81.6633	151.44	59.9392	1.33188	0.00032	0.07786	*Cystm1*	18
ENSMUSG00000026822	70.2995	59.3248	41.3674	355.473	109.666	93.3295	56.9973	186.156	-1.70425	0.00032	0.07786	*Lcn2*	2
ENSMUSG00000031518	0	0	0	2.09719	11.6459	20.6402	0	11.4611	-5.93266	0.00034	0.07996	*Spata4*	8
ENSMUSG00000062007	27.5085	10.3583	44.7215	1.04859	6.79346	0.8974	27.5294	2.91315	3.23689	0.00035	0.08319	*Hsh2d*	8

ENSMUSG00000034312	2693.8	1745.84	2843.17	1258.31	1184.97	1593.78	2427.6	1345.69	0.85084	0.00036	0.08322	*Iqsec1*	6
ENSMUSG00000070427	394.289	338.057	335.411	235.933	146.545	191.146	355.919	191.208	0.89744	0.00036	0.08322	*Il18bp*	7
ENSMUSG00000063652	988.269	489.665	1107.98	354.424	295.03	521.389	861.97	390.281	1.14213	0.00037	0.08322	*Slc22a21*	11
ENSMUSG00000020097	5767.62	4323.18	5802.62	3113.27	3371.5	3425.37	5297.8	3303.38	0.68133	0.00037	0.08322	*Sgpl1*	10
ENSMUSG00000015085	89.6574	64.0331	84.9709	31.4578	32.0263	33.2038	79.5538	32.2293	1.30147	0.00038	0.08497	*Entpd2*	2
ENSMUSG00000089854	0	0	0	4.19437	8.73444	17.948	0	10.2923	-5.77728	0.0004	0.08832	*Gm16133*	13
ENSMUSG00000042770	302.594	581.006	367.835	1694.53	1267.46	411.009	417.145	1124.33	-1.42973	0.00041	0.08845	*Hebp1*	6
ENSMUSG00000032854	2136.49	731.673	2336.7	364.91	664.788	942.269	1734.96	657.323	1.3996	0.00041	0.08845	*Ugt8a*	3
ENSMUSG00000028037	181.352	73.4498	121.866	24.1176	39.7902	63.7154	125.556	42.5411	1.55519	0.00041	0.08845	*Ifi44*	3
ENSMUSG00000021930	1155.36	588.54	1237.67	381.688	468.749	595.873	993.855	482.103	1.04274	0.00045	0.0948	*Spryd7*	14
ENSMUSG00000096768	222.106	177.974	292.926	1007.7	354.23	376.01	231.002	579.313	-1.32664	0.00046	0.09535	*Gm47283*	Y
ENSMUSG00000035504	113.091	97.933	150.935	367.008	248.446	176.788	120.653	264.081	-1.13077	0.00046	0.09568	*Reep6*	10
ENSMUSG00000030471	1204.26	542.398	1120.27	443.555	428.958	535.747	955.645	469.42	1.02494	0.00047	0.09568	*Zdhhc13*	7
ENSMUSG00000023908	228.219	302.274	336.529	592.455	473.601	469.34	289.007	511.799	-0.82454	0.00048	0.09623	*Pkmyt1*	17
ENSMUSG00000027048	0	0	0	3.14578	12.6164	14.3584	0	10.0402	-5.74071	0.00049	0.09623	*Abcb11*	2
ENSMUSG00000026601	63.1677	42.3749	51.4298	60.8184	152.368	257.554	52.3241	156.913	-1.58723	0.00049	0.09623	*Axdnd1*	1
ENSMUSG00000095248	2.03767	0	0	1.04859	21.3509	46.6648	0.67922	23.0214	-5.08306	0.0005	0.09623	*Olfr681*	7
ENSMUSG00000061322	29.5462	11.3	16.7706	32.5064	65.0231	121.149	19.2056	72.8928	-1.92932	0.0005	0.09623	*Dnaic1*	4

ENSMUSG00000050334	8.15067	1.88333	3.35411	15.7289	35.9083	31.409	4.4627	27.682	-2.64011	0.00051	0.09623	*C130071C03Rik*	13	
ENSMUSG00000024878	952.61	526.39	885.486	369.105	342.584	507.031	788.162	406.24	0.95518	0.00051	0.09623	*Cbwd1*	19	
ENSMUSG00000051378	441.155	372.899	433.799	984.629	672.552	591.386	415.951	749.522	-0.84928	0.00051	0.09623	*Kif18b*	11	
ENSMUSG00000024317	1965.33	1122.46	3107.03	800.077	816.185	1207.9	2064.94	941.387	1.13276	0.00051	0.09623	*Rnf138*	18	
ENSMUSG00000022902	13.2448	42.3749	6.70823	181.407	65.9936	43.9726	20.776	97.1243	-2.21213	0.00052	0.09623	*Stfa2*	16	
ENSMUSG00000055609	282.217	121.475	32.4231	42.9923	20.3804	43.9726	145.372	35.7818	2.02328	0.00052	0.09623	*Hba-x*	11	
ENSMUSG00000030699	8.15067	12.2416	13.4165	65.0128	33.9673	30.5116	11.2696	43.1639	-1.93572	0.00053	0.09623	*Tbx6*	7	
ENSMUSG00000020275	990.307	384.199	1039.78	288.363	381.404	406.522	804.76	358.763	1.1646	0.00053	0.09623	*Rel*	11	
ENSMUSG00000108379	0	0	0	3.14578	7.76395	19.7428	0	10.2175	-5.76739	0.00053	0.09623	*A730082K24Rik*	7	
ENSMUSG00000042708	76.4125	28.2499	95.0332	73.4015	257.181	341.909	66.5652	224.164	-1.75609	0.00054	0.09689	*Shcbp1l*	1	
ENSMUSG00000005686	200.71	210.933	277.273	806.368	593.942	244.093	229.639	548.134	-1.25502	0.00055	0.09741	*Ampd3*	7	
ENSMUSG00000095320	1.01883	0	2.23608	4.19437	11.6459	46.6648	1.08497	20.835	-4.32169	0.00056	0.09745	*Ccl21a*	JH584294.1	
ENSMUSG00000029561	603.15	341.824	458.396	193.99	178.571	313.192	467.79	228.584	1.03158	0.00056	0.09745	*Oasl2*	5	
ENSMUSG00000028456	3741.16	1745.84	3168.52	1093.68	1460.59	1773.26	2885.17	1442.51	0.99977	0.00056	0.09745	*Unc13b*	4	
ENSMUSG00000031710	114.109	179.858	197.893	79.6931	17.4689	68.2024	163.953	55.1214	1.57329	0.00058	0.09889	*Ucp1*	8	
ENSMUSG00000100084	74.3749	48.0248	55.9019	19.9233	12.6164	26.922	59.4339	19.8206	1.57996	0.00059	0.09983	*2310068J16Rik*	15	
ENSMUSG00000035671	2729.46	1436.04	1991.23	847.263	956.907	1382.89	2052.24	1062.35	0.94954	0.00059	0.09983	*Zswim4*	8	
ENSMUSG00000046179	231.275	313.574	386.841	1352.69	691.962	338.32	310.563	794.322	-1.35463	0.0006	0.09983	*E2f8*	7	

ENSMUSG00000026894	11.2072	2.82499	4.47215	9.43734	52.4067	53.844	6.16811	38.5627	-2.64992	0.0006	0.0999	*Morn5*	2
ENSMUSG00000024521	22.4143	3.76665	15.6525	14.6803	91.2264	113.97	13.9445	73.2922	-2.40229	0.00061	0.101	*Pmaip1*	18
ENSMUSG00000062519	1351.99	781.581	1387.49	614.476	671.582	666.768	1173.69	650.942	0.84994	0.00063	0.10303	*Zfp398*	6
ENSMUSG00000047220	1012.72	442.582	980.519	180.358	256.21	532.158	811.941	322.909	1.32884	0.00064	0.10355	*Ccdc36*	9
ENSMUSG00000104401	41.7722	15.0666	17.8886	6.29156	0	2.6922	24.9091	2.99459	3.07644	0.00065	0.10523	*Gm20750*	3
ENSMUSG00000072423	1.01883	1.88333	5.59019	4.19437	33.9673	43.0752	2.83078	27.0789	-3.2866	0.00068	0.108	*Psmb11*	14
ENSMUSG00000026049	1631.15	746.739	2256.2	403.708	589.09	935.09	1544.7	642.629	1.26453	0.0007	0.10897	*Tex30*	1
ENSMUSG00000043289	49.9229	40.4915	152.053	20.9719	14.5574	30.5116	80.8225	22.0136	1.86968	0.0007	0.10897	*Mei4*	9
ENSMUSG00000028132	297.5	134.658	235.906	865.089	677.405	226.145	222.688	589.546	-1.40467	0.0007	0.10897	*Tmem56*	3
ENSMUSG00000023443	8.15067	0.94166	0	8.38875	59.2001	26.0246	3.03078	31.2045	-3.35774	0.00071	0.10928	*Esx1*	X
ENSMUSG00000024491	2898.58	1206.27	2774.97	995.115	1085.01	1307.51	2293.27	1129.21	1.02177	0.00073	0.11189	*Rbm27*	18
ENSMUSG00000021573	170.145	85.6914	235.906	51.3811	45.6132	89.7399	163.914	62.2447	1.39212	0.00074	0.11233	*Tppp*	13
ENSMUSG00000029055	75.3937	26.3666	48.0756	16.7775	12.6164	13.461	49.9453	14.285	1.80669	0.00075	0.11407	*Plch2*	4
ENSMUSG00000038637	319.914	228.824	292.926	380.639	594.913	540.234	280.555	505.262	-0.84994	0.00077	0.11607	*Lrrc56*	7
ENSMUSG00000024905	873.141	435.049	881.014	183.504	260.092	492.672	729.734	312.089	1.22386	0.00078	0.11607	*Tesmin*	19
ENSMUSG00000116655	27.5085	11.3	38.0133	1.04859	3.88198	6.2818	25.6073	3.73745	2.74935	0.00079	0.11633	*AC154507.2*	17
ENSMUSG00000044471	0	0.94166	0	4.19437	12.6164	22.435	0.31389	13.0819	-5.16178	0.00082	0.1207	*Lncpint*	6
ENSMUSG00000023935	113.091	105.466	112.922	54.5268	48.5247	60.1258	110.493	54.3924	1.02114	0.00086	0.12462	*Spats1*	17

ENSMUSG00000051705	788.577	419.04	877.66	278.926	348.407	422.675	695.093	350.003	0.98855	0.00086	0.12462	*Senp8*	9
ENSMUSG00000041235	266.934	280.616	324.231	709.898	563.857	362.549	290.594	545.435	-0.90803	0.00087	0.12499	*Chd7*	4
ENSMUSG00000037254	6.113	4.70832	6.70823	7.34015	37.8493	58.331	5.84318	34.5068	-2.57128	0.00088	0.125	*Itih2*	2
ENSMUSG00000024269	1652.55	1096.1	1905.14	699.412	721.077	1093.03	1551.26	837.84	0.88803	0.00088	0.125	*Tpgs2*	18
ENSMUSG00000070291	580.735	191.158	613.803	106.957	163.043	262.938	461.899	177.646	1.37648	0.00089	0.12522	*Ddx43*	9
ENSMUSG00000109234	0	0	0	8.38875	10.6754	6.2818	0	8.44866	-5.48665	0.00089	0.12522	*Gm44561*	7
ENSMUSG00000054905	29.5462	45.1999	21.2427	202.378	69.8756	50.2544	31.9963	107.503	-1.74118	0.00091	0.12673	*Stfa3*	16
ENSMUSG00000071037	287.311	130.891	296.28	79.6931	97.0494	137.302	238.161	104.682	1.18258	0.00093	0.12869	*Camkmt*	17
ENSMUSG00000070814	989.288	677.998	783.745	361.765	465.837	568.054	817.01	465.219	0.81153	0.00094	0.12869	*Zswim9*	7
ENSMUSG00000056054	138.561	148.783	176.65	816.854	259.122	187.556	154.665	421.177	-1.4447	0.00095	0.12941	*S100a8*	3
ENSMUSG00000114230	7.13184	0.94166	22.3608	0	0	0	10.1448	0	5.82205	0.00099	0.13438	*Gm48239*	14
ENSMUSG00000028617	4674.41	3402.23	4987.57	2109.77	2272.9	3232.43	4354.74	2538.37	0.77844	0.001	0.13522	*Lrrc42*	4
ENSMUSG00000019814	2023.4	1013.23	2165.64	670.051	812.303	1135.21	1734.09	872.522	0.99034	0.00106	0.1416	*Ltv1*	10
ENSMUSG00000045942	209.88	107.35	243.732	37.7494	56.2886	116.662	186.987	70.2333	1.40728	0.00109	0.14492	*BC049762*	11
ENSMUSG00000079553	356.592	505.673	400.258	917.519	805.51	537.542	420.841	753.524	-0.83938	0.00112	0.14792	*Kifc1*	17
ENSMUSG00000032065	977.062	525.448	1383.01	210.767	362.965	627.282	961.841	400.338	1.26335	0.00113	0.14792	*Tex12*	9
ENSMUSG00000086741	5.09417	9.41664	4.47215	63.9642	24.2623	13.461	6.32765	33.8958	-2.40554	0.00116	0.15163	*Gm15816*	8
ENSMUSG00000071561	57.0547	93.2247	70.4364	398.465	109.666	116.662	73.5719	208.264	-1.49856	0.0012	0.15463	*BC100530*	16

ENSMUSG00000038203	2.03767	3.76665	2.23608	8.38875	19.4099	33.2038	2.68013	20.3341	-2.91726	0.0012	0.15463	*Hoxa13*	6
ENSMUSG00000005952	29.5462	12.2416	23.4788	1.04859	1.94099	6.2818	21.7555	3.09046	2.78413	0.00122	0.15631	*Trpv1*	11
ENSMUSG00000031352	2087.59	995.338	2778.32	575.678	865.68	1223.16	1953.75	888.171	1.13678	0.00123	0.15631	*Hccs*	X
ENSMUSG00000020089	1273.54	907.764	1352.83	590.358	701.667	815.736	1178.04	702.587	0.74487	0.00124	0.15631	*Ppa1*	10
ENSMUSG00000053897	176.258	250.483	305.224	974.143	659.936	217.171	243.988	617.083	-1.33842	0.00124	0.15631	*Slc39a8*	3
ENSMUSG00000108419	6.113	4.70832	0	10.4859	26.2033	41.2804	3.60711	25.9899	-2.8297	0.00126	0.15768	*Gm45020*	7
ENSMUSG00000015619	36.678	32.9582	52.5478	91.2276	74.728	108.585	40.728	91.5137	-1.17377	0.00129	0.15968	*Gata3*	2
ENSMUSG00000098358	2.03767	0	0	2.09719	71.8165	113.072	0.67922	62.3287	-6.51728	0.0013	0.1603	*Gm27164*	9
ENSMUSG00000032649	59.0924	53.6748	58.138	70.2557	205.745	136.405	56.9684	137.468	-1.27254	0.00132	0.16248	*Colgalt2*	1
ENSMUSG00000005045	211.917	123.358	172.178	28.312	87.3444	87.9451	169.151	67.8672	1.31319	0.00135	0.16473	*Chd5*	4
ENSMUSG00000083505	28.5274	8.47497	8.9443	1.04859	0.97049	1.7948	15.3155	1.2713	3.57765	0.0014	0.1703	*Gm7541*	16
ENSMUSG00000042282	22.4143	16.0083	14.5345	18.8747	72.787	99.6113	17.6524	63.7577	-1.85497	0.00143	0.17259	*Gucy2f*	X
ENSMUSG00000023832	1071.81	797.589	1097.91	626.01	637.614	624.59	989.105	629.405	0.65172	0.00144	0.17294	*Acat2*	17
ENSMUSG00000030029	992.344	692.123	1029.71	480.256	567.739	599.463	904.727	549.152	0.71943	0.00152	0.18157	*Lrig1*	6
ENSMUSG00000096992	61.13	19.7749	49.1937	3.14578	11.6459	16.1532	43.3662	10.315	2.05667	0.00159	0.18833	*Gm26788*	1
ENSMUSG00000109870	2.03767	0	0	2.09719	19.4099	26.922	0.67922	16.143	-4.57148	0.00161	0.19005	*Gm35850*	8
ENSMUSG00000035898	4161.94	1671.45	4382.71	1264.6	1607.14	2109.79	3405.37	1660.51	1.03592	0.00163	0.19129	*Uba6*	5
ENSMUSG00000031118	0	1.88333	0	3.14578	13.5869	29.6142	0.62778	15.449	-4.54565	0.00166	0.19384	*1700080O16Rik*	X

ENSMUSG00000027722	5852.18	2119.68	5132.91	1109.41	1718.74	2947.96	4368.26	1925.37	1.18168	0.0017	0.19724	*Spata5*	3
ENSMUSG00000107756	102.902	45.1999	84.9709	11.5345	34.9378	33.2038	77.691	26.5587	1.54098	0.00175	0.20117	*Gm44164*	6
ENSMUSG00000042303	2327.02	1067.85	2108.62	636.496	943.32	1210.59	1834.49	930.136	0.97935	0.00176	0.20192	*Sgsm3*	15
ENSMUSG00000046323	37.6969	23.5416	114.04	12.5831	1.94099	21.5376	58.4261	12.0206	2.27249	0.0018	0.20313	*Dppa3*	6
ENSMUSG00000087207	89.6574	18.8333	101.741	3.14578	14.5574	28.7168	70.0774	15.4733	2.16667	0.0018	0.20313	*Gm13147*	4
ENSMUSG00000067360	1.01883	0	6.70823	3.14578	39.7902	35.896	2.57569	26.2773	-3.39728	0.00181	0.20313	*Pramel3*	X
ENSMUSG00000019303	163.013	165.733	106.214	193.99	296.971	343.704	144.987	278.222	-0.93958	0.00181	0.20313	*Psmc3ip*	11
ENSMUSG00000026389	512.473	563.115	543.367	1093.68	1021.93	629.974	539.652	915.196	-0.76153	0.00184	0.20539	*Steap3*	1
ENSMUSG00000032690	275.085	155.374	150.935	115.345	83.4625	90.6373	193.798	96.4817	1.0077	0.00186	0.20646	*Oas2*	5
ENSMUSG00000083327	2275.06	2189.37	3702.94	1624.27	1641.11	1644.04	2722.46	1636.47	0.73409	0.00189	0.2073	*Vcp-rs*	X
ENSMUSG00000097290	121.241	126.183	165.47	226.496	245.535	233.324	137.631	235.118	-0.77467	0.0019	0.2073	*1300002E11Rik*	16
ENSMUSG00000035877	1855.3	1340.93	1512.71	1031.82	1042.31	1014.96	1569.64	1029.69	0.60815	0.00191	0.2073	*Zhx3*	2
ENSMUSG00000028251	46.8664	78.1581	50.3117	177.212	116.459	93.3295	58.4454	129	-1.13698	0.00192	0.2073	*Tstd3*	4
ENSMUSG00000073119	56.0359	34.8416	10.0623	58.7212	296.001	474.724	33.6466	276.482	-3.03749	0.00193	0.2073	*Speer4a*	5
ENSMUSG00000087086	0	0.94166	1.11804	2.09719	14.5574	27.8194	0.68657	14.8247	-4.4471	0.00193	0.2073	*Gm13269*	2
ENSMUSG00000030671	1594.48	498.14	1432.21	324.015	507.568	714.33	1174.94	515.305	1.18833	0.00198	0.21117	*Pde3b*	7
ENSMUSG00000025480	1349.95	629.031	1123.63	242.225	393.05	752.918	1034.2	462.731	1.15925	0.00205	0.21749	*Syce1*	7
ENSMUSG00000075268	118.185	105.466	88.325	56.624	48.5247	52.9466	103.992	52.6984	0.98234	0.00206	0.21754	*Gm10819*	19

ENSMUSG00000024493	2538.93	1516.08	2777.21	1405.11	1260.67	1390.07	2277.41	1351.95	0.75212	0.00211	0.22149	*Lars*	18
ENSMUSG00000052920	53.9982	16.0083	41.3674	14.6803	7.76395	5.3844	37.1246	9.27622	2.00955	0.00211	0.22149	*Prkg1*	19
ENSMUSG00000020000	39.7345	29.1916	35.7772	101.714	52.4067	90.6373	34.9011	81.5858	-1.22615	0.00213	0.22174	*Moxd1*	10
ENSMUSG00000030291	1489.54	936.014	2048.25	671.1	728.841	1011.37	1491.26	803.77	0.89102	0.00215	0.22339	*Med21*	6
ENSMUSG00000003452	328.065	263.666	456.16	187.698	205.745	205.504	349.297	199.649	0.80528	0.0022	0.22676	*Bicd1*	6
ENSMUSG00000050174	174.221	54.6165	93.9152	29.3606	34.9378	56.5362	107.584	40.2782	1.41286	0.00222	0.22793	*Nudt6*	3
ENSMUSG00000106508	62.1489	48.9665	72.6725	16.7775	22.3214	33.2038	61.2626	24.1009	1.337	0.00225	0.2288	*4933425M03Rik*	3
ENSMUSG00000045573	133.467	111.116	90.5611	203.427	149.456	324.859	111.715	225.914	-1.0161	0.00226	0.2288	*Penk*	4
ENSMUSG00000098519	12.226	3.76665	25.7149	2.09719	0	0	13.9025	0.69906	4.38445	0.00226	0.2288	*Gm27166*	9
ENSMUSG00000073608	4.07534	1.88333	10.0623	11.5345	38.8198	32.3064	5.34034	27.5536	-2.39409	0.00228	0.2288	*Gal3st2c*	1
ENSMUSG00000039782	1250.11	550.873	1498.17	584.066	523.096	586.002	1099.72	564.388	0.96192	0.00228	0.2288	*Cpeb2*	5
ENSMUSG00000026819	810.992	456.707	921.263	320.87	361.024	498.057	729.654	393.317	0.89026	0.00234	0.23321	*Slc25a25*	2
ENSMUSG00000057003	0	0	2.23608	2.09719	3.88198	48.4596	0.74536	18.1462	-4.6888	0.00236	0.23321	*Myh4*	11
ENSMUSG00000017737	12.226	24.4833	8.9443	77.5959	47.5542	27.8194	15.2179	50.9898	-1.73041	0.00236	0.23321	*Mmp9*	2
ENSMUSG00000035367	1921.52	886.105	1483.64	654.322	680.316	986.242	1430.42	773.627	0.88622	0.00238	0.23376	*Rmi1*	13
ENSMUSG00000063172	837.481	387.965	707.718	265.294	311.529	435.239	644.388	337.354	0.93244	0.00245	0.23984	*Hspb11*	4
ENSMUSG00000096916	687.713	275.907	679.767	201.33	212.538	364.344	547.796	259.404	1.07689	0.00249	0.24206	*Zfp850*	7
ENSMUSG00000074093	209.88	187.391	285.1	512.762	354.23	333.833	227.457	400.275	-0.81613	0.00251	0.24261	*Svip*	7

ENSMUSG00000009350	3.0565	71.5664	168.824	5.24297	2.91148	7.1792	81.1489	5.11121	3.98628	0.00252	0.24261	*Mpo*	11
ENSMUSG00000096950	46.8664	24.4833	43.6035	41.9437	96.0789	194.736	38.3177	110.919	-1.53863	0.00253	0.24261	*Gm9530*	1
ENSMUSG00000079597	49.9229	44.2582	44.7215	240.128	84.433	57.4336	46.3009	127.331	-1.45691	0.00254	0.24261	*Gm5483*	16
ENSMUSG00000033880	363.724	405.857	272.801	196.087	193.128	234.221	347.461	207.812	0.74196	0.00256	0.2439	*Lgals3bp*	11
ENSMUSG00000067562	4.07534	25.4249	102.86	2.09719	1.94099	13.461	44.1199	5.83306	2.90001	0.00259	0.24481	*Dmrtc1c1*	X
ENSMUSG00000043740	15.2825	10.3583	10.0623	24.1176	26.2033	78.9712	11.9011	43.0974	-1.86023	0.00265	0.24943	*B430306N03Rik*	17
ENSMUSG00000029521	252.671	239.183	228.08	591.407	407.607	292.552	239.978	430.522	-0.84197	0.00267	0.25078	*Chek2*	5
ENSMUSG00000055184	196.635	87.5747	191.185	60.8184	66.9641	92.4321	158.465	73.4049	1.10635	0.0027	0.25218	*Fam72a*	1
ENSMUSG00000027605	2318.87	1064.08	1952.09	749.744	918.087	1219.57	1778.35	962.466	0.88526	0.00274	0.25484	*Acss2*	2
ENSMUSG00000113852	410.59	102.641	253.795	52.4297	102.872	141.789	255.675	99.0304	1.36563	0.00281	0.25875	*Gm48062*	12
ENSMUSG00000114610	102.902	33.8999	65.9642	18.8747	27.1738	27.8194	67.5888	24.6226	1.45221	0.00281	0.25875	*Cyp2c52-ps*	19
ENSMUSG00000026679	17.3202	13.1833	10.0623	14.6803	47.5542	88.8425	13.5219	50.359	-1.89842	0.00285	0.26089	*Enkur*	2
ENSMUSG00000099145	7.13184	15.0666	8.9443	95.422	36.8788	9.87139	10.3809	47.3907	-2.18132	0.00286	0.26089	*Mir8116*	5
ENSMUSG00000045104	2.03767	5.64998	4.47215	4.19437	4.85247	97.8165	4.05327	35.6211	-3.13668	0.0029	0.264	*Gm5514*	19
ENSMUSG00000048960	113.091	100.758	93.9152	141.56	111.607	610.232	102.588	287.799	-1.48917	0.00293	0.26494	*Prex2*	1
ENSMUSG00000062518	43.8099	8.47497	45.8396	3.14578	4.85247	11.6662	32.7081	6.55481	2.30076	0.003	0.26903	*Zfp534*	4
ENSMUSG00000036712	2453.35	1094.21	2418.32	832.583	1031.63	1318.28	1988.63	1060.83	0.90614	0.00302	0.26903	*Cyld*	8
ENSMUSG00000031998	5.09417	0.94166	17.8886	0	0	0	7.97481	0	5.47413	0.00302	0.26903	*1700128F08Rik*	9

ENSMUSG00000033799	4911.8	1571.64	4861.23	1145.06	1817.73	2315.29	3781.55	1759.36	1.10369	0.00302	0.26903	*Fam208b*	13
ENSMUSG00000051166	1908.28	599.84	1533.95	357.57	578.414	884.836	1347.35	606.94	1.14984	0.00306	0.27144	*Eml5*	12
ENSMUSG00000035365	252.671	154.433	247.086	408.951	320.263	390.369	218.063	373.194	-0.77663	0.00311	0.27422	*Parpbp*	10
ENSMUSG00000054150	31.5839	28.2499	44.7215	117.442	95.1084	44.87	34.8518	85.8069	-1.30108	0.00312	0.27422	*Syne3*	12
ENSMUSG00000025408	175.239	260.841	225.844	770.716	398.873	238.708	220.641	469.432	-1.08804	0.00315	0.27524	*Ddit3*	10
ENSMUSG00000020838	11.2072	9.41664	17.8886	35.6522	42.7017	34.1012	12.8375	37.485	-1.55556	0.00318	0.2767	*Slc6a4*	11
ENSMUSG00000052298	2678.51	2127.22	2915.84	1754.3	1608.11	1826.21	2573.86	1729.54	0.57333	0.00319	0.2767	*Cdc42se2*	11
ENSMUSG00000107470	10.1883	15.0666	168.824	13.6317	12.6164	9.87139	64.6929	12.0398	2.42402	0.00327	0.28269	*Gm3375*	7
ENSMUSG00000026158	225.162	222.233	324.231	618.67	424.106	336.525	257.209	459.767	-0.83826	0.0033	0.28355	*Ogfrl1*	1
ENSMUSG00000038893	727.447	1018.88	1266.74	3136.34	1920.61	1030.21	1004.35	2029.05	-1.01446	0.00332	0.2848	*Fam117a*	11
ENSMUSG00000019295	1164.53	795.706	1115.8	654.322	666.729	673.947	1025.34	664.999	0.62426	0.00336	0.28524	*Tmem129*	5
ENSMUSG00000097532	100.865	87.5747	159.879	46.1381	69.8756	51.1518	116.106	55.7218	1.05588	0.00336	0.28524	*Gm4349*	3
ENSMUSG00000032131	202.748	274.024	239.26	797.979	662.847	181.275	238.677	547.367	-1.19664	0.00339	0.28632	*Abcg4*	9
ENSMUSG00000027678	2241.43	1284.43	1954.33	1043.35	1141.3	1208.8	1826.73	1131.15	0.69116	0.0034	0.28632	*Ncoa3*	2
ENSMUSG00000069270	19.3578	15.0666	31.3051	8.38875	2.91148	1.7948	21.9098	4.36501	2.34689	0.00343	0.28632	*Hist1h2ac*	13
ENSMUSG00000031904	1575.12	790.056	1587.61	612.378	718.165	887.528	1317.6	739.357	0.83293	0.00344	0.28632	*Slc7a6*	8
ENSMUSG00000069581	22.4143	22.5999	42.4854	8.38875	3.88198	10.7688	29.1666	7.67984	1.91718	0.00344	0.28632	*Tspear*	10
ENSMUSG00000038695	667.336	947.314	758.03	1717.6	1562.5	865.093	790.893	1381.73	-0.80441	0.00346	0.28736	*Josd2*	7

ENSMUSG00000040687	1094.23	748.623	1217.54	560.997	523.096	757.405	1020.13	613.833	0.73201	0.00348	0.28736	*Madd*	2
ENSMUSG00000047751	32.6027	22.5999	70.4364	2.09719	12.6164	16.1532	41.8797	10.2889	2.00856	0.00349	0.28736	*Utf1*	7
ENSMUSG00000007944	41.7722	16.9499	25.7149	3.14578	9.70494	8.07659	28.1457	6.97577	2.00179	0.00356	0.29081	*Ttc9b*	7
ENSMUSG00000001964	1471.2	1260.89	1334.94	979.386	920.999	945.859	1355.67	948.748	0.51491	0.00356	0.29081	*Emd*	X
ENSMUSG00000087390	17.3202	17.8916	8.9443	14.6803	64.0526	76.279	14.7187	51.6706	-1.80912	0.00363	0.29443	*Gm7598*	X
ENSMUSG00000003824	2020.35	1045.25	1876.07	657.468	785.129	1243.8	1647.22	895.464	0.87874	0.00363	0.29443	*Syce2*	8
ENSMUSG00000036151	254.708	94.1664	164.352	60.8184	65.0231	105.893	171.075	77.2449	1.14395	0.00365	0.29485	*Tm6sf2*	8
ENSMUSG00000040749	1554.74	645.04	1788.86	390.077	624.028	895.605	1329.55	636.57	1.0618	0.00368	0.29582	*Siah1b*	X
ENSMUSG00000005501	2494.11	1655.44	2650.87	1360.03	1408.19	1567.76	2266.81	1445.32	0.64895	0.00371	0.2968	*Usp40*	1
ENSMUSG00000029148	5336.65	3854.23	4713.65	2900.41	2798.9	3547.42	4634.84	3082.24	0.58841	0.00373	0.2975	*Nrbp1*	5
ENSMUSG00000018654	405.496	214.699	351.064	994.066	872.474	277.296	323.753	714.612	-1.14226	0.00375	0.29798	*Ikzf1*	11
ENSMUSG00000074971	40.7534	50.8498	54.7839	95.422	102.872	85.2529	48.7957	94.5158	-0.95426	0.00378	0.29803	*Fibin*	2
ENSMUSG00000111997	24.452	19.7749	25.7149	6.29156	2.91148	8.07659	23.3139	5.75988	2.01075	0.00378	0.29803	*Gm5176*	10
ENSMUSG00000115138	3.0565	5.64998	8.9443	16.7775	29.1148	26.0246	5.8836	23.9723	-2.03889	0.00387	0.30414	*Gm36899*	15
ENSMUSG00000034610	5460.95	2735.53	4623.09	2465.24	2256.4	2903.98	4273.19	2541.88	0.74929	0.00391	0.30495	*Tut4*	4
ENSMUSG00000103529	38.7157	28.2499	33.5411	175.115	118.4	21.5376	33.5022	105.018	-1.64677	0.00395	0.30495	*A730089K16Rik*	5
ENSMUSG00000024502	1.01883	0	0	1.04859	7.76395	23.3324	0.33961	10.715	-4.87499	0.004	0.30495	*Jakmip2*	18
ENSMUSG00000021259	121.241	125.241	125.22	147.852	229.037	307.808	123.901	228.232	-0.88302	0.00401	0.30495	*Cyp46a1*	12

ENSMUSG00000046532	144.674	151.608	148.699	251.662	230.007	224.35	148.327	235.34	-0.66536	0.00401	0.30495	*Ar*	X
ENSMUSG00000047344	122.26	86.6331	127.456	62.9156	50.4657	62.818	112.116	58.7331	0.93092	0.00402	0.30495	*Lancl3*	X
ENSMUSG00000006200	98.8269	53.6748	171.06	30.4092	29.1148	64.6128	107.854	41.3789	1.37519	0.00402	0.30495	*Rhox6*	X
ENSMUSG00000032221	537.944	598.898	264.975	1352.69	1151.01	465.75	467.272	989.814	-1.08192	0.00402	0.30495	*Mns1*	9
ENSMUSG00000017861	423.835	417.157	403.612	914.373	859.858	425.367	414.868	733.199	-0.82093	0.00404	0.30495	*Mybl2*	2
ENSMUSG00000086335	2.03767	0.94166	21.2427	0	0	0	8.07402	0	5.49147	0.00404	0.30495	*Gm12107*	11
ENSMUSG00000041703	2.03767	0	0	2.09719	8.73444	30.5116	0.67922	13.7811	-4.34472	0.00404	0.30495	*Zic5*	14
ENSMUSG00000040649	3061.6	1556.57	2705.65	1203.78	1259.7	1775.95	2441.27	1413.15	0.78839	0.0041	0.30824	*Rimklb*	6
ENSMUSG00000086429	806.916	465.182	745.731	454.041	348.407	410.112	672.61	404.187	0.73443	0.00417	0.31196	*Gt(ROSA)26Sor*	6
ENSMUSG00000079606	5.09417	0.94166	7.82627	2.09719	24.2623	71.792	4.6207	32.7172	-2.84466	0.00427	0.31747	*Gm595*	X
ENSMUSG00000097233	139.58	66.8581	126.338	35.6522	43.6722	69.9972	110.926	49.7738	1.1503	0.00428	0.31747	*Gm17552*	19
ENSMUSG00000027313	2.03767	3.76665	14.5345	92.2762	25.2328	5.3844	6.77961	40.9645	-2.60742	0.00429	0.31747	*Chac1*	2
ENSMUSG00000050966	121.241	57.4415	153.171	29.3606	40.7607	67.305	110.618	45.8088	1.26506	0.00436	0.32162	*Lin28a*	4
ENSMUSG00000024163	4680.52	3909.79	3914.25	2580.59	3249.21	2817.83	4168.19	2882.55	0.53204	0.0044	0.3239	*Mapk8ip3*	17
ENSMUSG00000115961	64.1865	11.3	46.9576	7.34015	9.70494	15.2558	40.8147	10.767	1.91277	0.00449	0.32746	*Gm41409*	16
ENSMUSG00000010048	639.828	625.265	807.224	1961.92	1391.69	640.743	690.772	1331.45	-0.94659	0.0045	0.32746	*Ifrd2*	9
ENSMUSG00000086681	49.9229	18.8333	49.1937	11.5345	17.4689	7.1792	39.3166	12.0609	1.70511	0.0045	0.32746	*Gm16178*	8
ENSMUSG00000029848	496.172	329.582	1107.98	83.8875	60.1706	260.246	644.577	134.768	2.25693	0.00455	0.33026	*Stra8*	6

ENSMUSG00000052748	1758.51	947.314	1871.6	774.91	887.031	1029.32	1525.81	897.086	0.76572	0.00461	0.33284	*Swt1*	1
ENSMUSG00000062209	3.0565	0.94166	3.35411	2.09719	21.3509	34.1012	2.45076	19.1831	-2.99061	0.00472	0.33973	*Erbb4*	1
ENSMUSG00000047547	153.844	263.666	159.879	484.45	370.729	234.221	192.463	363.133	-0.91351	0.00476	0.34141	*Cltb*	13
ENSMUSG00000045467	32.6027	30.1332	42.4854	45.0895	76.669	129.226	35.0738	83.6613	-1.26024	0.00478	0.34141	*Ttll13*	7
ENSMUSG00000058900	160.976	209.991	249.322	138.414	109.666	113.072	206.763	120.384	0.78064	0.00479	0.34141	*Rsl1*	13
ENSMUSG00000049526	4.07534	1.88333	5.59019	14.6803	16.4984	24.2298	3.84962	18.4695	-2.28356	0.00482	0.34151	*Tmem202*	9
ENSMUSG00000054003	100.865	53.6748	81.6168	84.936	368.788	765.482	78.7187	406.402	-2.36876	0.00485	0.34151	*Tdrd9*	12
ENSMUSG00000073478	182.371	109.233	101.741	52.4297	81.5215	64.6128	131.115	66.188	0.98546	0.00486	0.34151	*D730003I15Rik*	1
ENSMUSG00000050503	94.7516	63.0915	73.7905	33.555	31.0558	45.7674	77.2112	36.7927	1.06579	0.00486	0.34151	*Fbxl22*	9
ENSMUSG00000038187	1736.09	932.247	1788.86	763.376	713.313	1091.24	1485.73	855.975	0.79498	0.00488	0.34151	*Btbd10*	7
ENSMUSG00000029716	142.637	71.5664	103.978	401.611	352.289	69.9972	106.06	274.633	-1.37228	0.00489	0.34151	*Tfr2*	5
ENSMUSG00000090231	43.8099	9.41664	15.6525	3.14578	2.91148	7.1792	22.9597	4.41215	2.36641	0.00494	0.34398	*Cfb*	17
ENSMUSG00000055780	40.7534	20.7166	46.9576	24.1176	143.633	159.737	36.1425	109.163	-1.59999	0.00503	0.34711	*Usp26*	X
ENSMUSG00000027509	2191.51	1631.9	2174.58	1216.37	1292.7	1518.4	1999.33	1342.49	0.57423	0.00504	0.34711	*Rae1*	2
ENSMUSG00000037818	656.129	290.974	809.46	226.496	273.679	389.471	585.521	296.549	0.97997	0.00504	0.34711	*Abhd18*	3
ENSMUSG00000032285	87.6197	185.508	118.512	233.836	262.033	218.068	130.546	237.979	-0.86394	0.00505	0.34711	*Dnaja4*	9
ENSMUSG00000063297	16.3013	31.0749	33.5411	89.1304	50.4657	54.7414	26.9725	64.7792	-1.26313	0.00512	0.35054	*Luzp2*	7
ENSMUSG00000063972	1357.09	504.732	1296.92	225.448	529.89	695.485	1052.91	483.607	1.1216	0.00517	0.3526	*Nr6a1*	2

ENSMUSG00000079432	6.113	3.76665	1.11804	3.14578	24.2623	43.9726	3.6659	23.7936	-2.69169	0.00523	0.35494	*Gm15023*	X
ENSMUSG00000027233	166.07	64.9748	174.414	46.1381	35.9083	87.9451	135.153	56.6638	1.2492	0.00525	0.35494	*Patl2*	2
ENSMUSG00000022949	81.5067	64.9748	80.4987	39.8465	41.7312	31.409	75.6601	37.6623	1.00731	0.00527	0.35494	*Clic6*	16
ENSMUSG00000032344	238.407	104.525	195.657	79.6931	86.3739	109.483	179.529	91.8499	0.96411	0.00531	0.35494	*Mb21d1*	9
ENSMUSG00000113626	139.58	89.458	184.476	97.5192	36.8788	52.0492	137.838	62.149	1.15081	0.00531	0.35494	*Gm7240*	13
ENSMUSG00000074771	3.0565	10.3583	5.59019	8.38875	29.1148	49.357	6.335	28.9535	-2.18782	0.00532	0.35494	*Ankef1*	2
ENSMUSG00000033174	144.674	224.116	220.254	663.759	427.988	173.198	196.348	421.648	-1.10175	0.00532	0.35494	*Mgll*	6
ENSMUSG00000066175	63.1677	49.9082	80.4987	32.5064	23.2919	32.3064	64.5249	29.3682	1.13286	0.00535	0.3559	*2510046G10Rik*	7
ENSMUSG00000078671	1635.23	1608.36	1300.28	1861.25	2544.63	2334.14	1514.62	2246.67	-0.56875	0.00542	0.35717	*Chd2*	7
ENSMUSG00000032376	2626.55	1724.19	2885.66	1342.2	1444.09	1781.34	2412.13	1522.54	0.66346	0.00542	0.35717	*Usp3*	9
ENSMUSG00000018900	873.141	593.248	876.542	447.749	456.132	582.412	780.977	495.431	0.65563	0.00543	0.35717	*Slc22a5*	11
ENSMUSG00000045282	62.1489	122.416	137.519	290.46	228.066	136.405	107.361	218.31	-1.02342	0.00545	0.35738	*Tmem86b*	7
ENSMUSG00000056055	53.9982	30.1332	38.0133	13.6317	14.5574	17.948	40.7149	15.379	1.40033	0.00549	0.35781	*Sag*	1
ENSMUSG00000108293	17.3202	7.53331	20.1247	0	0.97049	4.487	14.9927	1.81916	2.99488	0.00552	0.35781	*Pla2g4c-ps*	7
ENSMUSG00000104432	56.0359	28.2499	70.4364	6.29156	12.6164	29.6142	51.5741	16.1741	1.65865	0.00553	0.35781	*A430027C01Rik*	9
ENSMUSG00000039824	338.253	295.682	349.946	210.767	185.364	238.708	327.96	211.613	0.63094	0.00554	0.35781	*Myl6b*	10
ENSMUSG00000097755	15.2825	16.0083	16.7706	67.11	42.7017	24.2298	16.0205	44.6805	-1.47606	0.00557	0.35781	*2010110K18Rik*	18
ENSMUSG00000046959	154.863	120.533	96.1513	427.826	369.758	87.0477	123.849	294.877	-1.25003	0.00557	0.35781	*Slc26a1*	5

ENSMUSG00000021044	596.018	450.115	621.629	282.072	328.027	429.854	555.921	346.651	0.67985	0.0056	0.35781	*Adck1*	12
ENSMUSG00000037868	2.03767	2.82499	1.11804	20.9719	10.6754	9.87139	1.99357	13.8396	-2.77219	0.0056	0.35781	*Egr2*	10
ENSMUSG00000024660	852.764	844.672	772.564	1742.76	1277.17	939.577	823.334	1319.84	-0.68031	0.00561	0.35781	*Incenp*	19
ENSMUSG00000022837	4951.53	2117.8	5136.27	1192.25	1762.42	3111.28	4068.53	2021.98	1.00848	0.00567	0.36079	*Iqcb1*	16
ENSMUSG00000050244	2722.32	1175.2	2404.9	883.964	1191.77	1435.84	2100.81	1170.52	0.84341	0.00572	0.36125	*Heatr1*	13
ENSMUSG00000022408	6.113	5.64998	6.70823	9.43734	36.8788	28.7168	6.15707	25.011	-2.02836	0.00573	0.36125	*Fam83f*	15
ENSMUSG00000047686	327.046	393.615	278.391	586.164	454.191	507.928	333.018	516.094	-0.63059	0.00573	0.36125	*Rtl3*	X
ENSMUSG00000029054	7.13184	16.0083	17.8886	3.14578	1.94099	0.8974	13.6762	1.99472	2.79839	0.00575	0.36129	*Gabrd*	4
ENSMUSG00000025630	2391.2	1565.04	3249.02	1507.88	1403.33	1480.71	2401.76	1463.97	0.71395	0.0058	0.36257	*Hprt*	X
ENSMUSG00000050106	126.335	162.908	254.913	683.683	394.02	157.942	181.385	411.882	-1.18326	0.0058	0.36257	*Tmc8*	11
ENSMUSG00000093223	40.7534	10.3583	13.4165	0	6.79346	3.5896	21.5094	3.46102	2.6215	0.00583	0.36324	*Gm27903*	15
ENSMUSG00000092417	326.027	297.566	367.835	202.378	202.833	239.606	330.476	214.939	0.61907	0.00597	0.37053	*Gpank1*	17
ENSMUSG00000069873	0	0	0	11.5345	7.76395	0.8974	0	6.73196	-5.15465	0.00599	1	*4930438A08Rik*	11
ENSMUSG00000024301	217.012	225.058	134.165	274.731	394.991	310.5	192.078	326.741	-0.7648	0.006	0.37144	*Kifc5b*	17
ENSMUSG00000032422	1597.53	1046.19	1594.32	976.24	856.946	943.167	1412.68	925.451	0.60993	0.00611	0.37644	*Snx14*	9
ENSMUSG00000044021	26.4897	5.64998	19.0066	5.24297	0	1.7948	17.0488	2.34592	2.8824	0.00612	0.37644	*Muc19*	15
ENSMUSG00000039410	84.5632	68.7414	80.4987	109.054	158.19	143.584	77.9345	136.943	-0.8155	0.00621	0.38075	*Prdm16*	4
ENSMUSG00000033220	219.049	284.382	187.83	830.486	511.45	178.582	230.421	506.84	-1.1359	0.00625	0.38204	*Rac2*	15

ENSMUSG00000006567	191.541	54.6165	95.0332	304.092	382.375	123.841	113.73	270.103	-1.24796	0.00629	0.38241	*Atp7b*	8
ENSMUSG00000032252	1966.35	1207.21	1948.74	873.478	1077.25	1254.56	1707.43	1068.43	0.67583	0.00632	0.38241	*Glce*	9
ENSMUSG00000101397	73.356	84.7497	77.1446	33.555	52.4067	29.6142	78.4168	38.5253	1.02671	0.00632	0.38241	*Mug-ps1*	6
ENSMUSG00000051331	179.315	142.191	133.047	190.844	285.325	283.578	151.518	253.249	-0.74168	0.00635	0.38241	*Cacna1c*	6
ENSMUSG00000031125	66.2242	64.0331	224.726	36.7008	42.7017	60.1258	118.328	46.5094	1.34201	0.00636	0.38241	*3830403N18Rik*	X
ENSMUSG00000031362	312.782	202.458	579.144	89.1304	151.397	257.554	364.794	166.027	1.13291	0.00636	0.38241	*Xlr4c*	X
ENSMUSG00000087107	129.392	108.291	101.741	520.102	238.741	79.8685	113.142	279.571	-1.30365	0.0065	0.38941	*AI662270*	11
ENSMUSG00000101450	71.3184	15.0666	41.3674	10.4859	16.4984	12.5636	42.5841	13.1826	1.68808	0.00652	0.38941	*Gm28941*	1
ENSMUSG00000027556	10.1883	16.0083	27.951	118.491	32.0263	26.922	18.0492	59.1464	-1.71419	0.0066	0.3923	*Car1*	3
ENSMUSG00000030041	2445.2	1594.24	3061.19	772.813	880.238	2020.05	2366.88	1224.37	0.95048	0.00662	0.3923	*M1ap*	6
ENSMUSG00000071350	860.915	397.382	1047.6	276.829	396.932	530.363	768.633	401.375	0.93614	0.00666	0.3923	*Setdb2*	14
ENSMUSG00000034203	504.323	449.174	590.324	273.683	402.755	314.09	514.607	330.176	0.63931	0.00667	0.3923	*Chchd4*	6
ENSMUSG00000022218	99.8457	38.6082	70.4364	14.6803	19.4099	43.9726	69.6301	26.0209	1.41116	0.00667	0.3923	*Tgm1*	14
ENSMUSG00000071568	1.01883	0.94166	3.35411	1.04859	35.9083	13.461	1.77154	16.806	-3.27407	0.00668	0.3923	*Gm5874*	6
ENSMUSG00000100376	8.15067	5.64998	8.9443	1.04859	0	0	7.58165	0.34953	4.44467	0.0067	0.39253	*Gm28060*	7
ENSMUSG00000028955	4800.75	2991.67	5660.63	2690.69	2762.03	3050.26	4484.35	2834.33	0.66173	0.00675	0.39253	*Vamp3*	4
ENSMUSG00000030729	1750.36	714.723	1901.78	651.176	774.454	948.551	1455.62	791.394	0.87863	0.00675	0.39253	*Pgm2l1*	7
ENSMUSG00000112847	86.6009	48.0248	77.1446	32.5064	32.0263	35.896	70.5901	33.4762	1.07323	0.00676	0.39253	*Gm47573*	10

ENSMUSG00000075569	1126.83	308.866	699.892	175.115	340.643	463.058	711.863	326.272	1.12451	0.00681	0.39442	*Rsph10b*	5
ENSMUSG00000035916	3.0565	0.94166	5.59019	9.43734	17.4689	24.2298	3.19612	17.0453	-2.44637	0.00688	0.39696	*Ptprq*	10
ENSMUSG00000039236	87.6197	150.666	190.066	507.519	357.142	115.765	142.784	326.808	-1.19432	0.00689	0.39696	*Isg20*	7
ENSMUSG00000024789	879.254	907.764	1201.89	1986.04	1456.71	1234.82	996.303	1559.19	-0.64618	0.00692	0.39726	*Jak2*	19
ENSMUSG00000078941	234.332	243.891	171.06	141.56	140.722	112.175	216.428	131.486	0.72149	0.00693	0.39726	*Ak6*	13
ENSMUSG00000022780	136.524	244.833	86.0889	320.87	286.296	283.578	155.815	296.914	-0.92688	0.00695	0.39726	*Meltf*	16
ENSMUSG00000107178	69.2807	32.9582	69.3184	14.6803	17.4689	34.1012	57.1858	22.0835	1.36276	0.00698	0.39775	*Gm42531*	5
ENSMUSG00000027869	1.01883	0.94166	1.11804	26.2148	4.85247	4.487	1.02618	11.8514	-3.52656	0.00709	0.40299	*Hsd3b6*	3
ENSMUSG00000048521	17.3202	9.41664	20.1247	2.09719	4.85247	1.7948	15.6205	2.91482	2.41972	0.00713	0.40329	*Cxcr6*	9
ENSMUSG00000097119	1512.97	752.389	1033.07	566.24	637.614	779.84	1099.47	661.232	0.73311	0.00715	0.40329	*B230354K17Rik*	17
ENSMUSG00000021676	168.108	200.574	209.073	390.077	273.679	265.63	192.585	309.795	-0.68505	0.00718	0.40329	*Iqgap2*	13
ENSMUSG00000022940	1896.05	1059.37	2067.25	881.867	890.913	1240.21	1674.22	1004.33	0.73675	0.0072	0.40329	*Pigp*	16
ENSMUSG00000001510	1.01883	3.76665	42.4854	1.04859	0.97049	1.7948	15.757	1.2713	3.61428	0.00721	0.40329	*Dlx3*	11
ENSMUSG00000054434	87.6197	143.133	120.748	343.939	220.302	129.226	117.167	231.155	-0.97813	0.00721	0.40329	*Tmem120b*	5
ENSMUSG00000029701	3003.52	2025.52	2009.11	1386.24	1665.37	1613.52	2346.05	1555.04	0.5932	0.00726	0.40395	*Rbm28*	6
ENSMUSG00000051499	147.731	80.9831	139.755	65.0128	65.9936	66.4076	122.823	65.8046	0.89815	0.00727	0.40395	*Zfp786*	6
ENSMUSG00000026209	2550.14	1956.78	2338.94	1607.49	1588.7	1664.68	2281.95	1620.29	0.49387	0.00729	0.40447	*Dnpep*	1
ENSMUSG00000038725	355.573	249.541	686.475	1454.4	954.966	403.83	430.53	937.731	-1.12352	0.00731	0.40449	*Pkhd1l1*	15

ENSMUSG00000027379	4386.08	1466.17	4725.95	1450.2	1729.42	2285.68	3526.07	1821.77	0.9525	0.00735	0.40527	*Bub1*	2
ENSMUSG00000043592	11.2072	10.3583	13.4165	62.9156	42.7017	11.6662	11.6606	39.0945	-1.74373	0.00743	0.40782	*Unc5cl*	17
ENSMUSG00000058741	144.674	96.9914	149.817	40.8951	39.7902	101.406	130.494	60.6972	1.0985	0.00743	0.40782	*Prr19*	7
ENSMUSG00000103174	19.3578	1.88333	19.0066	1.04859	0	2.6922	13.4159	1.24693	3.39781	0.00748	0.40891	*Gm37168*	1
ENSMUSG00000016487	3275.55	2123.45	3414.49	1857.06	1916.73	2060.43	2937.83	1944.74	0.59495	0.00749	0.40891	*Ppfibp1*	6
ENSMUSG00000024006	4120.16	2670.56	4380.47	2381.36	2270.96	2689.51	3723.73	2447.27	0.60539	0.00759	0.4128	*Stk38*	17
ENSMUSG00000092286	73.356	30.1332	59.256	10.4859	11.6459	34.1012	54.2484	18.7443	1.52174	0.0076	0.4128	*Dtnbos*	12
ENSMUSG00000031297	62.1489	42.3749	64.8462	30.4092	26.2033	20.6402	56.4566	25.7509	1.13394	0.00769	0.41657	*Slc7a3*	X
ENSMUSG00000107496	0	0	1.11804	3.14578	5.82296	15.2558	0.37268	8.07484	-4.46673	0.00774	0.41795	*4933431M02Rik*	6
ENSMUSG00000068245	68.2619	26.3666	42.4854	5.24297	17.4689	22.435	45.7046	15.0489	1.59169	0.00776	0.41816	*Phf11d*	14
ENSMUSG00000025069	36.678	24.4833	33.5411	35.6522	80.551	105.893	31.5675	74.0321	-1.2351	0.00783	0.42052	*Gsto2*	19
ENSMUSG00000058626	8.15067	14.125	4.47215	37.7494	55.3181	9.87139	8.91593	34.313	-1.92989	0.00787	0.42126	*Capn11*	17
ENSMUSG00000028551	195.616	275.907	307.46	652.225	484.276	272.809	259.661	469.77	-0.8548	0.00788	0.42126	*Cdkn2c*	4
ENSMUSG00000042433	8.15067	1.88333	4.47215	3.14578	30.0853	44.87	4.83538	26.0337	-2.43889	0.0079	0.42138	*Pih1h3b*	X
ENSMUSG00000029621	4876.14	3296.76	5520.87	2801.84	3004.65	3245.89	4564.59	3017.46	0.59698	0.00794	0.42165	*Arpc1a*	5
ENSMUSG00000038357	33.6215	28.2499	49.1937	160.435	49.4952	61.9206	37.0217	90.6168	-1.29237	0.00795	0.42165	*Camp*	9
ENSMUSG00000025893	252.671	159.141	297.398	114.297	135.869	157.942	236.403	136.036	0.79434	0.00807	0.42719	*Kbtbd3*	9
ENSMUSG00000005968	602.131	329.582	603.741	320.87	277.561	333.833	511.818	310.754	0.71896	0.00825	0.43487	*Tuft1*	3

ENSMUSG00000056758	1136	1153.54	1407.61	845.166	594.913	972.781	1232.38	804.287	0.6153	0.00826	0.43487	*Hmga2*	10
ENSMUSG00000005148	89.6574	48.9665	73.7905	133.171	125.194	122.944	70.8048	127.103	-0.84624	0.00833	0.43735	*Klf5*	14
ENSMUSG00000041406	1607.72	710.956	1508.23	513.811	748.251	891.118	1275.64	717.726	0.82906	0.00835	0.43753	*BC055324*	1
ENSMUSG00000046275	31.5839	10.3583	20.1247	2.09719	8.73444	2.6922	20.6889	4.50794	2.19442	0.00848	0.44321	*Tusc5*	11
ENSMUSG00000050592	116.147	185.508	162.116	471.867	345.496	132.815	154.59	316.726	-1.03337	0.00859	0.44608	*Fam78a*	2
ENSMUSG00000020897	455.419	451.057	503.117	1141.92	770.572	484.596	469.864	799.029	-0.76555	0.0086	0.44608	*Aurkb*	11
ENSMUSG00000030672	53.9982	50.8498	40.2494	112.199	104.813	64.6128	48.3658	93.8752	-0.95255	0.00862	0.44608	*Mylpf*	7
ENSMUSG00000055296	2694.82	1673.34	2513.35	1373.66	1605.2	1588.4	2293.83	1522.42	0.59113	0.00863	0.44608	*Tmem245*	4
ENSMUSG00000041229	5050.36	2052.83	4038.35	1544.58	2174.88	2604.25	3713.85	2107.9	0.8169	0.00866	0.44682	*Phf8*	X
ENSMUSG00000071745	0	0	0	4.19437	2.91148	9.87139	0	5.65908	-4.91345	0.00869	1	*DXBay18*	X
ENSMUSG00000037315	4195.56	1742.08	4283.2	1236.29	1753.68	2526.18	3406.95	1838.72	0.8895	0.00872	0.44861	*Jade3*	X
ENSMUSG00000003410	33.6215	23.5416	32.4231	32.5064	76.669	103.201	29.8621	70.7921	-1.25092	0.00875	0.44864	*Elavl3*	9
ENSMUSG00000049577	912.875	1216.63	971.575	3171.99	1837.14	892.912	1033.69	1967.35	-0.92811	0.00876	0.44864	*Zfpm1*	8
ENSMUSG00000035472	89.6574	129.95	163.234	498.082	257.181	104.996	127.614	286.753	-1.16747	0.00883	0.44925	*Slc25a21*	12
ENSMUSG00000059991	205.804	112.058	231.434	154.143	449.339	551.901	183.099	385.127	-1.07473	0.00888	0.44925	*Nptx2*	5
ENSMUSG00000060332	2.03767	5.64998	6.70823	10.4859	19.4099	31.409	4.79863	20.4349	-2.09952	0.00888	0.44925	*Tmc2*	2
ENSMUSG00000011257	2234.3	2889.02	2494.34	6772.86	4502.12	2302.73	2539.22	4525.9	-0.83368	0.00891	0.44925	*Pabpc4*	4
ENSMUSG00000052212	19.3578	19.7749	14.5345	71.3043	35.9083	30.5116	17.8891	45.9081	-1.35223	0.00893	0.44925	*Cd177*	7

ENSMUSG00000089409	9.16951	0.94166	14.5345	0	0.97049	0	8.21522	0.3235	4.55601	0.00893	0.44925	*Gm24884*	4
ENSMUSG00000025041	1657.64	1194.03	1807.87	1067.47	1012.23	1092.14	1553.18	1057.28	0.55452	0.00894	0.44925	*Nt5c2*	19
ENSMUSG00000086013	414.665	230.708	354.418	145.754	191.187	244.99	333.264	193.977	0.77861	0.00896	0.44925	*Gm15706*	6
ENSMUSG00000029516	452.362	465.182	623.865	1226.85	730.782	616.513	513.803	858.05	-0.73978	0.00897	0.44925	*Cit*	5
ENSMUSG00000011884	266.934	303.216	261.621	506.471	477.483	318.577	277.257	434.177	-0.64573	0.00899	0.4493	*Gltp*	5
ENSMUSG00000048279	4325.97	1751.49	3205.42	1675.65	1821.62	2000.3	3094.29	1832.52	0.75562	0.00902	0.44958	*Sacs*	14
ENSMUSG00000044197	107.996	153.491	225.844	594.552	313.47	147.174	162.444	351.732	-1.11447	0.00904	0.44958	*Gpr146*	5
ENSMUSG00000034640	1663.76	974.622	2062.78	832.583	758.926	1190.85	1567.05	927.453	0.75615	0.00909	0.45111	*Tiparp*	3
ENSMUSG00000020649	1156.38	1323.98	1634.57	4173.4	2746.5	1071.49	1371.64	2663.8	-0.9575	0.00913	0.45202	*Rrm2*	12
ENSMUSG00000016541	4019.3	3438.96	4491.16	2953.89	2477.67	3026.93	3983.14	2819.5	0.49835	0.00929	0.45889	*Atxn10*	15
ENSMUSG00000023025	3229.7	1649.79	3093.61	1470.13	1693.51	1804.67	2657.7	1656.1	0.68213	0.00932	0.45921	*Larp4*	15
ENSMUSG00000085586	18.339	32.9582	15.6525	36.7008	48.5247	78.9712	22.3166	54.7322	-1.28996	0.00942	0.46309	*Gm11613*	11
ENSMUSG00000105589	20.3767	3.76665	15.6525	1.04859	0	3.5896	13.2653	1.54606	3.06802	0.00956	0.46748	*Fbxw14*	9
ENSMUSG00000035864	158.938	115.825	138.637	230.69	210.597	207.299	137.8	216.196	-0.65032	0.00956	0.46748	*Syt1*	10
ENSMUSG00000006784	20.3767	22.5999	21.2427	36.7008	43.6722	65.5102	21.4064	48.6277	-1.18623	0.00963	0.47003	*Ttc25*	11
ENSMUSG00000038507	1545.57	812.656	1604.38	556.803	707.49	1032.91	1320.87	765.733	0.78585	0.00967	0.47082	*Parp12*	6
ENSMUSG00000027297	35.6592	20.7166	21.2427	20.9719	71.8165	122.944	25.8728	71.9107	-1.47784	0.00969	0.47082	*Ltk*	2
ENSMUSG00000045624	1944.95	983.097	1336.06	916.47	971.464	785.225	1421.37	891.053	0.67369	0.00977	0.4735	*Esf1*	2

ENSMUSG00000027994	28.5274	68.7414	17.8886	207.621	117.43	26.0246	38.3858	117.025	-1.60141	0.00981	0.47407	*Mcub*	3
ENSMUSG00000049755	1957.18	1256.18	1886.13	1144.02	1111.22	1189.05	1699.83	1148.09	0.56586	0.00986	0.47537	*Zfp672*	11
ENSMUSG00000024483	5114.55	2393.71	3933.26	2065.73	2288.42	2708.35	3813.84	2354.17	0.69587	0.0099	0.4754	*Ankhd1*	18
ENSMUSG00000059108	11.2072	13.1833	5.59019	74.4501	28.1443	9.87139	9.99355	37.4886	-1.89498	0.0099	0.4754	*Ifitm6*	7
ENSMUSG00000024758	6180.25	4498.33	6767.48	4131.46	3958.64	4040.09	5815.35	4043.4	0.52421	0.00996	0.47684	*Rtn3*	19
ENSMUSG00000094295	3.0565	1.88333	0	5.24297	14.5574	17.948	1.64661	12.5828	-2.91283	0.00998	0.47684	*Olfr819*	10
ENSMUSG00000025877	9.16951	16.0083	15.6525	52.4297	23.2919	34.9986	13.6101	36.9067	-1.43691	0.01007	0.47886	*Hk3*	13
ENSMUSG00000011179	4483.89	4470.08	4838.87	8002.86	5777.35	5828.61	4597.61	6536.27	-0.50754	0.01012	0.47886	*Odc1*	12
ENSMUSG00000050335	7.13184	20.7166	5.59019	50.3325	29.1148	26.0246	11.1462	35.1573	-1.63931	0.01013	0.47886	*Lgals3*	14
ENSMUSG00000027496	430.967	475.54	527.714	1336.96	704.579	499.851	478.074	847.129	-0.82491	0.01015	0.47886	*Aurka*	2
ENSMUSG00000028282	1951.07	1036.77	1854.83	942.685	950.113	1157.65	1614.22	1016.81	0.66637	0.01016	0.47886	*Casp8ap2*	4
ENSMUSG00000035246	127.354	178.916	234.788	583.018	340.643	167.814	180.353	363.825	-1.0121	0.01016	0.47886	*Pcyt1b*	X
ENSMUSG00000087475	24.452	9.41664	26.8329	2.09719	3.88198	8.07659	20.2339	4.68525	2.08864	0.01025	0.4821	*4933406I18 Rik*	7
ENSMUSG00000086493	2.03767	1.88333	1.11804	5.24297	7.76395	24.2298	1.67968	12.4122	-2.88232	0.01034	0.48522	*Gm12204*	11
ENSMUSG00000074224	285.274	92.283	206.837	39.8465	76.669	139.097	194.798	85.2042	1.1891	0.0104	0.48682	*4932431P2 0Rik*	7
ENSMUSG00000049092	778.389	270.257	834.056	157.289	276.591	462.161	627.568	298.68	1.06972	0.01043	0.48698	*Gpr137c*	14
ENSMUSG00000033769	2323.96	1482.18	2326.64	1373.66	1232.53	1490.58	2044.26	1365.59	0.58177	0.01047	0.48701	*Exoc6b*	6
ENSMUSG00000064061	5559.78	1992.56	4807.56	1594.91	2126.35	3010.78	4119.97	2244.01	0.87635	0.01047	0.48701	*Dzip3*	16

ENSMUSG00000028331	188.484	161.966	176.65	270.537	248.446	267.425	175.7	262.136	-0.57763	0.01054	0.48841	*Trmo*	4
ENSMUSG00000090098	23.4332	12.2416	24.5968	4.19437	4.85247	7.1792	20.0905	5.40868	1.8812	0.01055	0.48841	*Alms1-ps2*	6
ENSMUSG00000046733	90.6762	113	90.5611	65.0128	55.3181	43.9726	98.079	54.7678	0.84498	0.01062	0.48952	*Gprc5a*	6
ENSMUSG00000037415	942.421	1369.18	1794.45	3696.29	2418.47	1343.41	1368.68	2486.06	-0.86103	0.01062	0.48952	*Ranbp10*	8
ENSMUSG00000002109	1370.33	676.114	1207.48	527.442	603.647	823.813	1084.64	651.634	0.73436	0.01069	0.49154	*Ddb2*	2
ENSMUSG00000032456	132.448	245.774	241.496	563.095	397.902	210.889	206.573	390.629	-0.91829	0.01077	0.49272	*Nmnat3*	9
ENSMUSG00000005802	2273.02	1323.04	2385.89	1197.49	1162.65	1470.84	1993.98	1276.99	0.64253	0.01079	0.49272	*Slc30a4*	2
ENSMUSG00000035560	1.01883	0	4.47215	4.19437	13.5869	24.2298	1.83033	14.0037	-2.98898	0.01081	0.49272	*Wdr20rt*	12
ENSMUSG00000029784	52.9794	28.2499	45.8396	32.5064	102.872	186.659	42.3563	107.346	-1.34598	0.01085	0.49272	*Ssmem1*	6
ENSMUSG00000030979	472.739	702.481	829.584	2217.77	1172.36	570.746	668.268	1320.29	-0.98217	0.01086	0.49272	*Uros*	7
ENSMUSG00000001089	3400.87	1474.65	2476.45	1012.94	1470.3	1795.7	2450.66	1426.31	0.78058	0.01086	0.49272	*Luzp1*	4
ENSMUSG00000049124	127.354	160.083	133.047	308.286	260.092	153.455	140.161	240.611	-0.77734	0.01094	0.49332	*Gm8186*	17
ENSMUSG00000021209	1238.9	357.832	1425.5	148.9	250.387	460.366	1007.41	286.551	1.81329	0.01094	0.49332	*Ppp4r4*	12
ENSMUSG00000072889	3222.57	1740.19	3289.27	1312.84	1677.01	2078.38	2750.68	1689.41	0.70294	0.01094	0.49332	*Nfxl1*	5
ENSMUSG00000045842	1.01883	7.53331	4.47215	3.14578	3.88198	85.2529	4.34143	30.7602	-2.82266	0.01103	0.49504	*Tgif2-ps2*	17
ENSMUSG00000000440	220.068	50.8498	207.955	38.7979	66.9641	95.1243	159.624	66.9621	1.24924	0.01104	0.49504	*Pparg*	6
ENSMUSG00000025498	122.26	91.3414	72.6725	61.867	48.5247	43.0752	95.4246	51.1556	0.90339	0.01106	0.49504	*Irf7*	7
ENSMUSG00000080935	32.6027	15.0666	31.3051	6.29156	12.6164	5.3844	26.3248	8.09746	1.69884	0.01111	0.49504	*Got2-ps1*	5

ENSMUSG00000031367	1561.87	709.073	1845.88	610.281	782.218	948.551	1372.28	780.35	0.81376	0.01115	0.49504	*Ap1s2*	X
ENSMUSG00000028640	15.2825	15.0666	29.069	3.14578	0.97049	8.97399	19.806	4.36342	2.16088	0.01117	0.49504	*Tfap2c*	2
ENSMUSG00000107096	393.27	209.991	462.868	192.941	169.836	249.477	355.376	204.085	0.79832	0.01117	0.49504	*Gm43597*	5
ENSMUSG00000039954	3.0565	5.64998	6.70823	28.312	22.3214	9.87139	5.13824	20.1683	-1.97208	0.01117	0.49504	*Stk32a*	18
ENSMUSG00000030086	810.992	472.715	949.214	297.8	386.257	595.873	744.307	426.643	0.80155	0.01122	0.49622	*Chchd6*	6
ENSMUSG00000031659	444.212	332.407	410.32	1170.23	744.369	329.346	395.646	747.981	-0.91841	0.01129	0.49741	*Adcy7*	8
ENSMUSG00000022805	11.2072	1.88333	2.23608	5.24297	34.9378	34.9986	5.10886	25.0598	-2.29707	0.0113	0.49741	*Maats1*	16
ENSMUSG00000100199	248.595	92.283	115.158	67.11	35.9083	103.201	152.012	68.7397	1.14303	0.01132	0.49741	*Gm20324*	15
ENSMUSG00000042312	49.9229	80.0414	43.6035	115.345	104.813	98.7139	57.8559	106.291	-0.87183	0.01138	0.49859	*S100a13*	3
ENSMUSG00000028078	1433.5	592.306	1347.24	502.276	622.087	802.275	1124.35	642.213	0.80728	0.0114	0.49859	*Dclk2*	3
ENSMUSG00000026348	19.3578	17.8916	23.4788	76.5473	34.9378	34.9986	20.2428	48.8279	-1.26943	0.01145	0.49989	*Acmsd*	1
ENSMUSG00000073077	18.339	14.125	12.2984	20.9719	36.8788	61.9206	14.9208	39.9237	-1.42277	0.01152	0.50192	*Gm7173*	X
ENSMUSG00000037573	808.954	1011.35	822.876	1174.42	1187.88	1336.23	881.059	1232.85	-0.48446	0.01162	0.50341	*Tob1*	11
ENSMUSG00000087523	34.6404	46.1415	31.3051	49.2839	86.3739	90.6373	37.3623	75.4317	-1.01246	0.01163	0.50341	*Gm12319*	11
ENSMUSG00000038518	2700.93	1959.6	2788.39	1572.89	1736.21	1881.85	2482.97	1730.32	0.52073	0.01165	0.50341	*Jarid2*	13
ENSMUSG00000034127	70.2995	88.5164	183.358	470.818	205.745	109.483	114.058	262.015	-1.20065	0.01167	0.50341	*Tspan8*	10
ENSMUSG00000025876	146.712	159.141	159.879	108.005	73.7575	103.201	155.244	94.9879	0.70903	0.01168	0.50341	*Unc5a*	13
ENSMUSG00000024539	2029.52	1313.62	1782.15	1170.23	1008.34	1294.05	1708.43	1157.54	0.56134	0.0117	0.50354	*Ptpn2*	18

ENSMUSG00000036292	68.2619	91.3414	79.3807	108.005	128.105	178.582	79.6613	138.231	-0.79611	0.01174	0.50354	*Gramd1c*	16
ENSMUSG00000098661	1.01883	5.64998	1.11804	22.0205	13.5869	8.97399	2.59562	14.8605	-2.48228	0.01175	0.50354	*Mir7052*	7
ENSMUSG00000039252	203.767	205.283	111.804	303.043	281.443	269.22	173.618	284.569	-0.71027	0.01182	0.50392	*Lgi2*	5
ENSMUSG00000035133	4927.08	2407.83	5612.55	2186.32	2367.03	3169.61	4315.82	2574.32	0.74524	0.01183	0.50392	*Arhgap5*	12
ENSMUSG00000020262	1097.28	556.523	897.785	299.898	541.536	627.282	850.531	489.572	0.7959	0.01183	0.50392	*Adarb1*	10
ENSMUSG00000029269	2.03767	0	0	1.04859	9.70494	21.5376	0.67922	10.7637	-3.98827	0.01187	0.50431	*Sult1b1*	5
ENSMUSG00000043991	560.359	385.14	624.983	320.87	372.67	335.627	523.494	343.056	0.60875	0.0119	0.50453	*Pura*	18
ENSMUSG00000000253	496.172	886.105	796.043	2011.2	1260.67	698.177	726.107	1323.35	-0.86556	0.01192	0.50453	*Gmpr*	13
ENSMUSG00000079845	426.891	287.207	717.78	195.038	216.42	365.242	477.293	258.9	0.88053	0.01198	0.50604	*Xlr4a*	X
ENSMUSG00000115422	0	0	0	1.04859	2.91148	13.461	0	5.80702	-4.95374	0.01201	1	*4930452G13Rik*	14
ENSMUSG00000033054	2289.32	1162.01	2596.08	1150.31	1135.48	1408.92	2015.81	1231.57	0.7105	0.01208	0.50902	*Npat*	9
ENSMUSG00000021798	24.452	19.7749	8.9443	31.4578	44.6427	56.5362	17.7238	44.2122	-1.3142	0.01214	0.50932	*Ldb3*	14
ENSMUSG00000068859	4.07534	2.82499	5.59019	3.14578	26.2033	31.409	4.16351	20.2527	-2.29691	0.01216	0.50932	*Sp9*	2
ENSMUSG00000021697	83.5444	85.6914	201.247	428.875	205.745	148.071	123.494	260.897	-1.08035	0.01216	0.50932	*Depdc1b*	13
ENSMUSG00000102151	35.6592	14.125	26.8329	10.4859	6.79346	7.1792	25.539	8.15286	1.64973	0.01231	0.51286	*Gm37472*	14
ENSMUSG00000003992	576.66	649.748	600.386	824.194	848.212	827.402	608.931	833.269	-0.45229	0.01231	0.51286	*Ssbp2*	13
ENSMUSG00000031715	6842.49	3790.2	7271.72	3464.55	3648.09	4355.08	5968.13	3822.57	0.6426	0.01232	0.51286	*Smarca5*	8
ENSMUSG00000038267	251.652	201.516	323.113	674.245	377.522	302.424	258.76	451.397	-0.8029	0.01235	0.51314	*Slc22a23*	13

ENSMUSG00000020181	187.465	58.3831	193.421	71.3043	69.8756	71.792	146.423	70.9906	1.04231	0.0125	0.51847	*Nav3*	10
ENSMUSG00000042510	26.4897	66.8581	57.0199	257.954	114.518	33.2038	50.1226	135.225	-1.42939	0.01253	0.51859	*AA986860*	1
ENSMUSG00000042476	194.597	159.141	260.503	1106.27	842.389	182.172	204.747	710.276	-1.79464	0.01261	0.52096	*Abcb4*	5
ENSMUSG00000056445	8.15067	15.0666	6.70823	38.7979	28.1443	20.6402	9.97517	29.1942	-1.53485	0.01273	0.52471	*Hoxaas2*	6
ENSMUSG00000038425	341.309	251.424	287.336	318.772	518.244	561.772	293.356	466.263	-0.66957	0.01277	0.52484	*Poli*	18
ENSMUSG00000036966	737.636	612.081	820.64	468.721	456.132	573.438	723.452	499.43	0.53365	0.0128	0.52484	*Spryd3*	15
ENSMUSG00000036985	739.673	672.348	796.043	527.442	522.126	547.414	736.021	532.327	0.46695	0.01281	0.52484	*Zdhhc9*	X
ENSMUSG00000002076	922.045	439.757	920.145	169.872	363.935	613.821	760.649	382.543	0.99035	0.01286	0.52564	*Hsf2bp*	17
ENSMUSG00000074580	127.354	87.5747	121.866	34.6036	47.5542	87.9451	112.265	56.701	0.97928	0.01288	0.52564	*4931440P22Rik*	3
ENSMUSG00000047040	5.09417	5.64998	7.82627	25.1662	21.3509	15.2558	6.19014	20.591	-1.7372	0.01294	0.52639	*Prr15l*	11
ENSMUSG00000027702	156.9	106.408	204.601	74.4501	61.1411	116.662	155.97	84.0844	0.88717	0.01295	0.52639	*Lrrc34*	3
ENSMUSG00000024027	6.113	0.94166	8.9443	0	0	0	5.33299	0	4.895	0.01297	1	*Glp1r*	17
ENSMUSG00000112569	1.01883	11.3	6.70823	76.5473	17.4689	4.487	6.34234	32.8344	-2.36206	0.01302	0.52834	*Gm33111*	12
ENSMUSG00000099338	3.0565	11.3	10.0623	57.6726	28.1443	8.07659	8.1396	31.2978	-1.93737	0.01306	0.52893	*2810030D12Rik*	8
ENSMUSG00000097857	23.4332	3.76665	10.0623	2.09719	0	2.6922	12.4207	1.59646	2.95133	0.01308	0.52895	*Gm26603*	2
ENSMUSG00000030850	1521.12	1099.86	1556.31	919.616	1007.37	1003.29	1392.43	976.76	0.51111	0.01322	0.53345	*Ate1*	7
ENSMUSG00000012126	87.6197	165.733	102.86	181.407	203.804	214.478	118.737	199.896	-0.74933	0.01327	0.53388	*Ubxn11*	4
ENSMUSG00000025324	168.108	122.416	188.948	99.6164	88.3149	104.098	159.824	97.3432	0.71319	0.01328	0.53388	*Atp10a*	7

ENSMUSG00000055403	224.143	102.641	320.877	37.7494	87.3444	158.84	215.887	94.6445	1.18557	0.01337	0.5351	*4933427D06Rik*	6
ENSMUSG00000013629	1608.74	1345.64	1896.19	1018.18	1250	1141.49	1616.86	1136.56	0.50811	0.01344	0.5351	*Cad*	5
ENSMUSG00000038056	7837.89	2978.48	6345.98	2827.01	3311.32	3986.25	5720.79	3374.86	0.76127	0.01346	0.5351	*Kmt2c*	5
ENSMUSG00000078160	153.844	72.5081	144.227	65.0128	76.669	57.4336	123.526	66.3718	0.89472	0.01347	0.5351	*Gm16503*	4
ENSMUSG00000079467	89.6574	57.4415	114.04	31.4578	41.7312	57.4336	87.0463	43.5409	0.99249	0.01347	0.5351	*Gm14966*	19
ENSMUSG00000114853	28.5274	7.53331	22.3608	0	2.91148	8.07659	19.4738	3.66269	2.38033	0.01348	0.5351	*D030051J21Rik*	14
ENSMUSG00000001120	628.621	465.182	569.081	327.161	360.053	440.623	554.295	375.946	0.55899	0.01354	0.5351	*Pcbp3*	10
ENSMUSG00000040738	1936.8	1434.15	2421.67	1283.48	1282.02	1372.12	1930.88	1312.54	0.55653	0.01355	0.5351	*Ints8*	4
ENSMUSG00000062591	143.656	129.95	110.686	91.2276	65.0231	74.4842	128.097	76.9116	0.7384	0.01355	0.5351	*Tubb4a*	17
ENSMUSG00000002068	392.251	385.14	431.563	827.34	746.31	389.471	402.985	654.374	-0.69896	0.01357	0.53513	*Ccne1*	7
ENSMUSG00000031170	157.919	239.183	282.864	859.846	417.312	167.814	226.655	481.657	-1.08703	0.01366	0.53666	*Slc38a5*	X
ENSMUSG00000039611	303.613	217.524	230.316	178.261	162.072	148.968	250.484	163.101	0.61972	0.01368	0.53666	*Tmem246*	4
ENSMUSG00000005649	15.2825	8.47497	19.0066	3.14578	0.97049	4.487	14.2547	2.86776	2.3013	0.01369	0.53666	*Cabp5*	7
ENSMUSG00000026833	1886.88	2754.37	2978.45	1932.56	1362.57	1796.59	2539.9	1697.24	0.58158	0.01372	0.53677	*Olfm1*	2
ENSMUSG00000014907	843.595	535.807	932.444	535.831	417.312	542.927	770.615	498.69	0.62726	0.01379	0.53739	*Naf1*	8
ENSMUSG00000075704	207.842	381.374	383.487	983.58	642.467	271.015	324.234	632.354	-0.96316	0.01381	0.53739	*Txnrd2*	16
ENSMUSG00000046603	336.215	203.399	400.258	164.629	198.951	208.197	313.291	190.592	0.7149	0.01381	0.53739	*Tcaim*	9
ENSMUSG00000021171	3495.62	1884.27	3756.61	1934.65	1953.6	1997.61	3045.5	1961.96	0.6342	0.01392	0.53881	*Esyt2*	12

ENSMUSG00000041718	4007.07	1399.31	4163.57	943.734	1558.61	2443.62	3189.99	1648.66	0.95197	0.01393	0.53881	*Alg13*	X
ENSMUSG00000116589	92.7139	18.8333	44.7215	5.24297	15.5279	28.7168	52.0896	16.4959	1.64877	0.01393	0.53881	*CT030190.1*	16
ENSMUSG00000001228	1796.2	1598.94	1867.12	2579.54	2699.91	2035.3	1754.09	2438.25	-0.47514	0.01399	0.53967	*Uhrf1*	17
ENSMUSG00000054945	102.902	68.7414	105.096	40.8951	55.3181	55.6388	92.2464	50.6173	0.86152	0.014	0.53967	*Gm9958*	5
ENSMUSG00000024867	77.4314	145.016	147.581	450.895	191.187	126.533	123.343	256.205	-1.0534	0.01404	0.54023	*Pip5k1b*	19
ENSMUSG00000071753	0	0	0	5.24297	5.82296	3.5896	0	4.88518	-4.69593	0.0141	1	*C230004F18Rik*	X
ENSMUSG00000029330	100.865	110.175	163.234	177.212	204.774	222.555	124.758	201.514	-0.69503	0.01412	0.54241	*Cds1*	5
ENSMUSG00000007656	2522.63	2163.94	3091.38	1832.94	1761.45	1956.33	2592.65	1850.24	0.48646	0.01422	0.54522	*Arpp19*	9
ENSMUSG00000106173	0	0	0	1.04859	2.91148	12.5636	0	5.50789	-4.87745	0.01429	1	*Gm42764*	5
ENSMUSG00000036752	3256.19	3105.61	3574.37	5781.94	4634.11	3754.72	3312.06	4723.59	-0.51211	0.01436	0.54958	*Tubb4b*	2
ENSMUSG00000069300	57.0547	21.6583	16.7706	11.5345	8.73444	11.6662	31.8278	10.6451	1.5812	0.01442	0.55074	*Hist1h2bj*	13
ENSMUSG00000040729	857.858	272.141	651.816	208.67	284.355	439.726	593.938	310.917	0.93256	0.01452	0.55253	*Cep126*	9
ENSMUSG00000031824	676.506	452.94	640.636	331.355	414.401	436.136	590.027	393.964	0.58158	0.01452	0.55253	*6430548M08Rik*	8
ENSMUSG00000109536	171.164	75.3331	150.935	61.867	85.4035	66.4076	132.477	71.226	0.89308	0.01469	0.55741	*9330162G02Rik*	7
ENSMUSG00000033943	5505.78	2709.17	4895.89	2163.25	2818.31	3245	4370.28	2742.19	0.67222	0.0147	0.55741	*Mga*	2
ENSMUSG00000114891	20.3767	0.94166	6.70823	0	0.97049	0.8974	9.34219	0.62263	3.87334	0.01477	0.55906	*Gm47272*	18
ENSMUSG00000110177	6.113	0	12.2984	0	0	0	6.13714	0	5.09765	0.01482	1	*Gm6524*	8
ENSMUSG00000044465	2783.45	1484.06	2284.15	1153.45	1361.6	1698.78	2183.89	1404.61	0.63639	0.01487	0.56174	*Fam160a2*	7

ENSMUSG00000030717	69.2807	45.1999	41.3674	19.9233	19.4099	32.3064	51.9493	23.8798	1.11714	0.01492	0.56224	*Nupr1*	7
ENSMUSG00000034543	4939.31	2455.86	4286.56	1912.63	2400.03	3006.29	3893.91	2439.65	0.67434	0.01494	0.56224	*Morc2a*	11
ENSMUSG00000038115	24.452	17.8916	33.5411	12.5831	5.82296	7.1792	25.2949	8.52843	1.57261	0.01501	0.56396	*Ano2*	6
ENSMUSG00000067261	1.01883	0	0	6.29156	4.85247	8.07659	0.33961	6.40687	-4.12811	0.01519	1	*Foxd3*	4
ENSMUSG00000078498	16.3013	11.3	46.9576	7.34015	5.82296	8.07659	24.853	7.0799	1.8032	0.01521	0.56938	*Zfp988*	4
ENSMUSG00000091834	49.9229	18.8333	20.1247	9.43734	14.5574	3.5896	29.6269	9.19478	1.69465	0.01521	0.56938	*Gm17177*	13
ENSMUSG00000004677	3901.12	2489.76	3128.27	1784.71	2296.19	2419.39	3173.05	2166.76	0.55013	0.01526	0.57038	*Myo9b*	8
ENSMUSG00000032187	5327.48	4170.63	5106.08	3327.19	3468.54	3901	4868.06	3565.58	0.44908	0.0154	0.57422	*Smarca4*	9
ENSMUSG00000020273	1469.16	672.348	1513.82	605.038	652.172	922.527	1218.44	726.579	0.74519	0.01558	0.57925	*Papolg*	11
ENSMUSG00000029787	1767.68	1030.18	1935.32	1046.5	995.727	1069.7	1577.73	1037.31	0.60465	0.01559	0.57925	*Avl9*	6
ENSMUSG00000019992	173.202	109.233	146.463	224.399	219.332	221.658	142.966	221.796	-0.63489	0.01574	0.58236	*Mtfr2*	10
ENSMUSG00000034218	2473.73	712.839	1838.05	620.767	989.904	1099.31	1674.87	903.328	0.89035	0.01576	0.58236	*Atm*	9
ENSMUSG00000015880	884.348	492.49	1005.12	1291.87	1181.09	1230.33	793.985	1234.43	-0.6373	0.01579	0.58236	*Ncapg*	5
ENSMUSG00000038843	144.674	121.475	282.864	675.294	310.558	166.916	183.004	384.256	-1.07076	0.0158	0.58236	*Gcnt1*	19
ENSMUSG00000042554	4.07534	1.88333	14.5345	0	0.97049	0	6.83105	0.3235	4.28783	0.01581	1	*Zp3r*	1
ENSMUSG00000025089	15.2825	6.59165	11.1804	49.2839	16.4984	29.6142	11.0182	31.7988	-1.53083	0.01581	0.58236	*Gfra1*	19
ENSMUSG00000033392	2381.01	1509.49	2918.08	1323.32	1350.93	1740.06	2269.53	1471.44	0.62478	0.01586	0.58262	*Clasp2*	9
ENSMUSG00000002633	6.113	15.0666	23.4788	2.09719	3.88198	3.5896	14.8861	3.18959	2.21152	0.01587	0.58262	*Shh*	5

ENSMUSG00000045374	453.381	607.373	551.193	1018.18	853.064	584.207	537.316	818.485	-0.60652	0.01592	0.58326	*Wdr81*	11
ENSMUSG00000029428	671.412	767.456	883.25	1944.09	1356.75	675.742	774.039	1325.53	-0.77586	0.01606	0.58677	*Stx2*	5
ENSMUSG00000027074	1086.08	1313.62	1413.2	3305.17	1898.29	1220.46	1270.97	2141.3	-0.75237	0.01607	0.58677	*Slc43a3*	2
ENSMUSG00000029458	1428.41	1050.9	1463.51	887.11	953.025	967.397	1314.27	935.844	0.48948	0.01623	0.59153	*Brap*	5
ENSMUSG00000004460	3102.35	1939.83	2209.24	1415.6	1639.16	1881.85	2417.14	1645.54	0.55454	0.01636	0.59399	*Dnajb11*	16
ENSMUSG00000103788	12.226	15.0666	55.9019	139.463	92.1969	19.7428	27.7315	83.8009	-1.60053	0.01636	0.59399	*Gm37527*	6
ENSMUSG00000029776	1858.35	1678.99	2277.44	1415.6	1344.13	1460.97	1938.26	1406.9	0.46195	0.01641	0.59399	*Hibadh*	6
ENSMUSG00000063445	245.539	387.965	301.87	479.207	531.831	400.24	311.792	470.426	-0.59233	0.01641	0.59399	*Nmral1*	16
ENSMUSG00000031931	1484.44	678.939	1489.23	586.164	749.221	875.862	1217.54	737.082	0.72343	0.01646	0.5947	*Ankrd49*	9
ENSMUSG00000036117	0	0	0	4.19437	2.91148	7.1792	0	4.76168	-4.66299	0.0165	1	*Il5*	11
ENSMUSG00000038292	383.082	193.041	315.287	90.179	196.04	207.299	297.136	164.506	0.85067	0.01661	0.59915	*Ccdc155*	7
ENSMUSG00000020467	71.3184	58.3831	73.7905	111.151	99.9609	124.739	67.8307	111.95	-0.72545	0.01673	0.601	*Efemp1*	11
ENSMUSG00000042826	1433.5	1599.89	1215.31	1031.82	1111.22	927.014	1416.23	1023.35	0.46918	0.01674	0.601	*Fgf11*	11
ENSMUSG00000109724	124.298	197.749	508.707	139.463	161.102	106.791	276.918	135.785	1.02721	0.01675	0.601	*Gm18194*	7
ENSMUSG00000050705	1935.78	1066.9	1918.55	697.314	996.697	1329.95	1640.41	1007.99	0.70201	0.01685	0.60146	*2310061I04 Rik*	17
ENSMUSG00000025977	337.234	133.716	371.189	67.11	93.1674	234.221	280.713	131.5	1.09091	0.01686	0.60146	*Boll*	1
ENSMUSG00000020261	458.475	454.824	385.723	519.054	677.405	645.23	433.007	613.896	-0.50349	0.01688	0.60146	*Slc36a1*	11
ENSMUSG00000042682	1551.68	1363.53	1653.58	1069.57	1030.66	1243.8	1522.93	1114.68	0.44983	0.01689	0.60146	*Selenok*	14

ENSMUSG00000054252	336.215	266.491	334.293	162.532	222.243	235.119	312.333	206.631	0.59401	0.01691	0.60146	*Fgfr3*	5
ENSMUSG00000086480	27.5085	17.8916	5.59019	1.04859	1.94099	7.1792	16.9968	3.38959	2.30547	0.01696	0.6018	*Gm15287*	13
ENSMUSG00000058019	35.6592	5.64998	25.7149	7.34015	3.88198	6.2818	22.3413	5.83464	1.93533	0.01698	0.6018	*Ces5a*	8
ENSMUSG00000037617	473.758	306.982	282.864	202.378	203.804	269.22	354.535	225.134	0.65452	0.01706	0.60363	*Spag1*	15
ENSMUSG00000020669	93.7327	159.141	181.122	525.345	208.656	145.379	144.665	293.127	-1.01798	0.01711	0.60451	*Sh3yl1*	12
ENSMUSG00000108319	0	0	0	1.04859	2.91148	11.6662	0	5.20876	-4.79687	0.01712	1	*Gm44552*	7
ENSMUSG00000031109	526.737	461.415	556.783	411.049	337.732	346.396	514.978	365.059	0.49646	0.01717	0.60491	*Enox2*	X
ENSMUSG00000037426	1597.53	971.797	1494.82	700.46	887.031	1082.26	1354.72	889.918	0.60562	0.01719	0.60491	*Depdc5*	5
ENSMUSG00000102030	17.3202	16.9499	15.6525	2.09719	3.88198	7.1792	16.6409	4.38612	1.90684	0.01725	0.60491	*Gm29106*	1
ENSMUSG00000052525	735.598	321.107	963.749	162.532	301.824	543.824	673.485	336.06	1.0015	0.01727	0.60491	*Spdya*	17
ENSMUSG00000103598	12.226	6.59165	1.11804	1.04859	0	0	6.64523	0.34953	4.26106	0.01733	1	*Gm4856*	3
ENSMUSG00000048264	2229.21	1122.46	2145.52	878.721	1141.3	1409.81	1832.4	1143.28	0.68009	0.01736	0.60491	*Dip2c*	13
ENSMUSG00000039155	66.2242	17.8916	26.8329	20.9719	5.82296	9.87139	36.9829	12.2221	1.60456	0.01739	0.60491	*Cdh26*	2
ENSMUSG00000020423	384.1	771.223	780.391	1903.2	1044.25	623.693	645.238	1190.38	-0.88323	0.01739	0.60491	*Btg2*	1
ENSMUSG00000047014	20.3767	5.64998	24.5968	1.04859	1.94099	7.1792	16.8745	3.38959	2.28636	0.01741	0.60491	*Catsperb*	12
ENSMUSG00000002944	132.448	106.408	223.608	327.161	222.243	234.221	154.155	261.208	-0.76296	0.01741	0.60491	*Cd36*	5
ENSMUSG00000010592	10144.5	3425.77	13290.1	1381	2346.65	4791.22	8953.47	2839.62	1.65669	0.01741	0.60491	*Dazl*	17
ENSMUSG00000033904	3675.95	1428.5	2906.9	1183.86	1653.72	1922.23	2670.45	1586.6	0.75088	0.01745	0.60505	*Ccp110*	7

ENSMUSG00000078737	10.1883	2.82499	5.59019	1.04859	0	0	6.20117	0.34953	4.15509	0.01754	1	*Gm2128*	7
ENSMUSG00000074637	13.2448	19.7749	52.5478	3.14578	6.79346	14.3584	28.5225	8.09921	1.79793	0.01756	0.60649	*Sox2*	3
ENSMUSG00000007035	1827.79	546.165	1210.84	364.91	567.739	934.193	1194.93	622.281	0.94066	0.01759	0.60649	*Msh5*	17
ENSMUSG00000030031	260.821	105.466	317.523	116.394	130.046	126.533	227.937	124.324	0.8725	0.01761	0.60649	*Kbtbd8*	6
ENSMUSG00000074656	3589.35	2480.34	4095.37	2412.81	2087.53	2512.72	3388.36	2337.69	0.53533	0.01761	0.60649	*Eif2s2*	2
ENSMUSG00000053289	3800.25	1661.09	3652.63	1171.28	1597.43	2492.98	3037.99	1753.9	0.79225	0.01766	0.60649	*Ddx10*	9
ENSMUSG00000081965	187.465	21.6583	62.6101	19.9233	48.5247	28.7168	90.5779	32.3882	1.48204	0.01766	0.60649	*Gm11620*	11
ENSMUSG00000097818	1.01883	3.76665	10.0623	0	0	0	4.94928	0	4.78777	0.01769	1	*Gm26681*	2
ENSMUSG00000074235	0	0.94166	0	10.4859	1.94099	7.1792	0.31389	6.53537	-4.15316	0.01777	1	*Gm10649*	8
ENSMUSG00000041912	1642.36	580.065	1362.89	333.453	620.146	939.577	1195.1	631.058	0.92058	0.01778	0.6095	*Tdrkh*	3
ENSMUSG00000027287	1328.56	971.797	1271.21	916.47	854.035	787.019	1190.52	852.508	0.48174	0.01793	0.61358	*Snap23*	2
ENSMUSG00000030655	9563.79	4205.47	7077.18	3500.2	4640.9	4951.85	6948.82	4364.32	0.67092	0.01799	0.61448	*Smg1*	7
ENSMUSG00000087351	36.678	12.2416	24.5968	7.34015	6.79346	9.87139	24.5055	8.00167	1.60827	0.01806	0.61572	*Gm11464*	2
ENSMUSG00000024691	461.532	398.324	478.52	1070.61	648.29	457.674	446.125	725.526	-0.70116	0.01808	0.61572	*Fam111a*	19
ENSMUSG00000020183	88.6386	116.766	154.289	58.7212	78.61	71.792	119.898	69.7077	0.77959	0.01813	0.6163	*Cpm*	10
ENSMUSG00000113768	1.01883	2.82499	11.1804	0	0	0	5.00807	0	4.80336	0.01813	1	*Gm9237*	12
ENSMUSG00000030688	585.83	753.331	466.222	955.268	1268.44	645.23	601.794	956.311	-0.66743	0.01819	0.61724	*Stard10*	7
ENSMUSG00000026688	480.89	1103.63	613.803	2081.46	1324.72	675.742	732.774	1360.64	-0.89223	0.0183	0.62012	*Mgst3*	1

ENSMUSG00000073633	183.39	114.883	181.122	70.2557	92.1969	119.354	159.798	93.9356	0.76246	0.01835	0.62026	*Fbxo36*	1
ENSMUSG00000050702	18.339	10.3583	13.4165	13.6317	24.2623	92.4321	14.0379	43.4421	-1.63593	0.01839	0.62026	*4930563M21Rik*	9
ENSMUSG00000053916	5.09417	16.0083	4.47215	35.6522	10.6754	40.383	8.52487	28.9035	-1.7474	0.0184	0.62026	*Nanp*	2
ENSMUSG00000041147	5123.72	1574.46	3955.62	1292.92	1944.87	2622.2	3551.27	1953.33	0.86219	0.01842	0.62026	*Brca2*	5
ENSMUSG00000097613	148.75	48.0248	110.686	27.2634	36.8788	74.4842	102.487	46.2088	1.14325	0.01846	0.62045	*Gm17597*	15
ENSMUSG00000070336	1333.65	399.265	1078.91	234.885	468.749	717.022	937.275	473.552	0.98409	0.01863	0.62493	*Fbxo47*	11
ENSMUSG00000036095	98.8269	38.6082	51.4298	22.0205	18.4394	42.1778	62.955	27.5459	1.18702	0.01865	0.62493	*Dgkb*	12
ENSMUSG00000016024	114.109	129.95	117.394	155.192	103.843	513.312	120.484	257.449	-1.09648	0.01875	0.62707	*Lbp*	2
ENSMUSG00000112381	29.5462	12.2416	16.7706	4.19437	4.85247	8.07659	19.5195	5.70781	1.76361	0.01895	0.63179	*Gm8170*	10
ENSMUSG00000027860	338.253	342.766	432.681	714.092	528.919	430.752	371.233	557.921	-0.58762	0.01896	0.63179	*Vangl1*	3
ENSMUSG00000020021	1280.67	590.423	1263.38	653.274	615.293	698.177	1044.83	655.581	0.67193	0.01902	0.63179	*Fgd6*	10
ENSMUSG00000077416	0	0	0	2.09719	3.88198	8.07659	0	4.68525	-4.64216	0.01906	1	*Gm23747*	15
ENSMUSG00000086573	4.07534	1.88333	7.82627	0	0	0	4.59498	0	4.68027	0.01907	1	*Gm15384*	X
ENSMUSG00000050944	158.938	66.8581	163.234	31.4578	74.728	83.4581	129.677	63.2147	1.03155	0.01909	0.63179	*Efcab5*	11
ENSMUSG00000026418	735.598	750.506	646.226	642.788	365.876	406.522	710.777	471.729	0.59248	0.0191	0.63179	*Tnni1*	1
ENSMUSG00000038372	597.037	255.191	594.796	197.136	250.387	375.113	482.341	274.212	0.81309	0.0191	0.63179	*Gmds*	13
ENSMUSG00000038418	236.369	187.391	183.358	382.737	305.706	240.503	202.373	309.648	-0.61233	0.01914	0.63179	*Egr1*	18
ENSMUSG00000031432	623.526	455.765	500.881	615.524	845.3	820.223	526.724	760.349	-0.53011	0.01915	0.63179	*Prps1*	X

ENSMUSG00000027715	1320.41	1211.92	1689.36	3699.44	2038.04	1351.48	1407.23	2362.99	-0.74769	0.01916	0.63179	*Ccna2*	3
ENSMUSG00000033998	82.5255	79.0997	51.4298	48.2353	29.1148	34.9986	71.0183	37.4496	0.92841	0.01929	0.63504	*Kcnk1*	8
ENSMUSG00000101838	16.3013	7.53331	15.6525	3.14578	3.88198	1.7948	13.1624	2.94085	2.16592	0.01934	0.63558	*Gm18800*	1
ENSMUSG00000032261	683.638	417.157	648.462	292.557	334.82	487.288	583.086	371.555	0.64872	0.01937	0.63566	*Sh3bgrl2*	9
ENSMUSG00000004655	487.003	539.573	724.489	2677.06	2203.02	480.109	583.688	1786.73	-1.61405	0.01943	0.63594	*Aqp1*	6
ENSMUSG00000063810	3051.41	976.505	2281.92	671.1	1069.48	1662.88	2103.28	1134.49	0.89022	0.01944	0.63594	*Alms1*	6
ENSMUSG00000002346	1047.36	557.465	1035.3	606.087	559.004	561.772	880.043	575.621	0.61204	0.01959	0.63976	*Slc25a42*	8
ENSMUSG00000048787	695.864	389.849	641.754	318.772	382.375	424.47	575.822	375.206	0.6168	0.0197	0.64113	*Dcun1d3*	7
ENSMUSG00000045275	218.03	187.391	158.761	167.775	327.056	507.928	188.061	334.253	-0.83055	0.01972	0.64113	*Lca5l*	16
ENSMUSG00000041708	8.15067	6.59165	13.4165	10.4859	32.9968	41.2804	9.38626	28.2544	-1.60337	0.01972	0.64113	*Mpped1*	15
ENSMUSG00000022018	377.987	462.357	550.075	1556.11	850.153	308.705	463.473	904.99	-0.96512	0.01984	0.64382	*Rgcc*	14
ENSMUSG00000109957	0	1.88333	0	22.0205	5.82296	0.8974	0.62778	9.58027	-3.85729	0.01994	0.64546	*Gm45353*	8
ENSMUSG00000037533	2677.5	1211.92	2554.72	1129.33	1306.28	1590.19	2148.04	1341.94	0.67836	0.01995	0.64546	*Rapgef6*	11
ENSMUSG00000113905	10.1883	0.94166	3.35411	0	0	0	4.82804	0	4.75621	0.01996	1	*Gm48872*	13
ENSMUSG00000055407	6.113	1.88333	2.23608	4.19437	14.5574	30.5116	3.4108	16.4211	-2.27402	0.02	0.64554	*Map6*	7
ENSMUSG00000006057	1146.19	1281.6	1390.84	1093.68	845.3	802.275	1272.88	913.753	0.47853	0.02002	0.64554	*Atp5g1*	11
ENSMUSG00000085304	0	0	0	3.14578	0.97049	10.7688	0	4.96169	-4.7251	0.02008	1	*Gm12802*	4
ENSMUSG00000066151	1642.36	1009.46	1446.74	960.511	896.736	996.113	1366.19	951.12	0.52217	0.02008	0.64556	*Fkbp15*	4

ENSMUSG00000027833	8.15067	0	2.23608	14.6803	20.3804	14.3584	3.46225	16.473	-2.25827	0.02011	0.64556	*Shox2*	3
ENSMUSG00000026404	252.671	222.233	197.893	283.12	328.997	365.242	224.265	325.786	-0.53887	0.02012	0.64556	*Ddx59*	1
ENSMUSG00000090523	764.125	972.739	1122.51	2722.15	1368.4	883.041	953.125	1657.86	-0.79839	0.02016	0.64556	*Gypc*	18
ENSMUSG00000117284	737.636	484.015	898.903	495.985	404.696	496.262	706.851	465.647	0.60145	0.02017	0.64556	*Gm7072*	17
ENSMUSG00000083038	11.2072	4.70832	12.2984	0	1.94099	1.7948	9.40464	1.24526	2.88411	0.0202	0.64556	*Gm13233*	4
ENSMUSG00000036167	1664.77	1022.65	1857.06	902.839	940.409	1178.29	1514.83	1007.18	0.5883	0.02026	0.64626	*Pphln1*	15
ENSMUSG00000030704	3611.77	3266.63	5610.32	3239.1	2546.58	2685.02	4162.9	2823.57	0.55999	0.0203	0.64668	*Rab6a*	7
ENSMUSG00000105044	39.7345	32.9582	34.6592	15.7289	10.6754	20.6402	35.784	15.6815	1.18652	0.0204	0.64875	*Gm10416*	5
ENSMUSG00000010803	20.3767	18.8333	13.4165	37.7494	33.9673	43.0752	17.5421	38.2639	-1.12181	0.02048	0.65001	*Gabra1*	11
ENSMUSG00000026622	317.876	253.308	421.5	477.11	493.981	488.185	330.895	486.426	-0.55745	0.0205	0.65001	*Nek2*	1
ENSMUSG00000034730	107.996	123.358	84.9709	135.269	180.512	185.762	105.442	167.181	-0.66371	0.02066	0.65276	*Adgrb1*	15
ENSMUSG00000024169	1197.13	1257.12	1443.39	3196.11	1862.38	1252.77	1299.21	2103.75	-0.69516	0.02066	0.65276	*Ift140*	17
ENSMUSG00000041225	2719.27	2050	2606.15	1707.11	1787.65	1933	2458.47	1809.25	0.44213	0.02071	0.65276	*Arhgap12*	18
ENSMUSG00000106870	259.803	131.833	219.135	119.54	104.813	144.481	203.59	122.945	0.72566	0.02073	0.65276	*Gm43681*	5
ENSMUSG00000111521	42.791	23.5416	55.9019	16.7775	10.6754	23.3324	40.7448	16.9284	1.25945	0.02074	0.65276	*Gm48529*	9
ENSMUSG00000024145	250.633	178.916	329.821	162.532	134.899	179.48	253.123	158.97	0.66901	0.02081	0.65365	*Pigf*	17
ENSMUSG00000031644	2516.52	1233.58	2715.71	1048.59	1361.6	1618.01	2155.27	1342.74	0.6823	0.02086	0.65365	*Nek1*	8
ENSMUSG00000023952	1266.41	1436.98	1398.67	2644.55	2145.76	1351.48	1367.35	2047.27	-0.58206	0.02087	0.65365	*Gtpbp2*	17

ENSMUSG00000038383	1234.83	992.513	1064.37	790.639	853.064	795.993	1097.24	813.232	0.43204	0.0209	0.65365	*Pigu*	2
ENSMUSG00000106206	23.4332	6.59165	2.23608	1.04859	0	2.6922	10.7536	1.24693	3.08747	0.02096	0.65392	*Gm43094*	5
ENSMUSG00000097615	9.16951	6.59165	16.7706	67.11	29.1148	8.97399	10.8439	35.0663	-1.69722	0.02097	0.65392	*Gm2061*	1
ENSMUSG00000041552	5.09417	7.53331	1.11804	0	0	0	4.58184	0	4.68847	0.02098	1	*Ptchd1*	X
ENSMUSG00000074832	0	0	0	0	1.94099	16.1532	0	6.03139	-5.00787	0.02098	1	*2410141K09Rik*	13
ENSMUSG00000114553	4.07534	0.94166	2.23608	2.09719	22.3214	17.0506	2.41769	13.823	-2.52756	0.02103	0.65495	*Gm7644*	14
ENSMUSG00000105561	12.226	13.1833	6.70823	1.04859	0.97049	3.5896	10.7058	1.86956	2.49696	0.02109	0.65499	*Gm43462*	3
ENSMUSG00000030867	461.532	551.815	414.792	1102.07	648.29	517.799	476.046	756.054	-0.66634	0.0211	0.65499	*Plk1*	7
ENSMUSG00000009487	5.09417	0.94166	6.70823	5.24297	23.2919	26.0246	4.24802	18.1865	-2.1217	0.02116	0.65581	*Otog*	7
ENSMUSG00000020932	57.0547	105.466	157.643	452.992	169.836	89.7399	106.721	237.523	-1.15407	0.02125	0.65733	*Gfap*	11
ENSMUSG00000098975	3.0565	3.76665	1.11804	11.5345	14.5574	10.7688	2.64706	12.2869	-2.19464	0.02128	0.65733	*Gm27177*	14
ENSMUSG00000026229	3241.93	2081.08	3600.08	2025.88	1972.04	2170.81	2974.36	2056.24	0.53235	0.0213	0.65733	*Psmd1*	1
ENSMUSG00000047369	60.1112	27.3082	62.6101	78.6445	74.728	139.097	50.0099	97.4898	-0.96927	0.02133	0.65733	*Dnah14*	1
ENSMUSG00000103108	0	0	0	0	6.79346	9.87139	0	5.55495	-4.88768	0.0214	1	*Rncr4*	6
ENSMUSG00000036185	110.034	57.4415	112.922	28.312	43.6722	69.0998	93.4658	47.028	0.984	0.02142	0.6591	*Sapcd1*	17
ENSMUSG00000031595	169.126	202.458	139.755	315.627	214.479	253.067	170.446	261.057	-0.61263	0.02145	0.6591	*Pdgfrl*	8
ENSMUSG00000026205	6.113	5.64998	2.23608	18.8747	14.5574	16.1532	4.66635	16.5284	-1.81069	0.02153	0.6605	*Slc23a3*	1
ENSMUSG00000005672	727.447	627.148	906.729	1046.5	1056.87	1032.01	753.775	1045.12	-0.47214	0.02166	0.66333	*Kit*	5

ENSMUSG00000043122	16.3013	24.4833	13.4165	41.9437	42.7017	34.1012	18.067	39.5822	-1.12291	0.02172	0.66333	*A530016L24Rik*	12
ENSMUSG00000087013	41.7722	39.5499	40.2494	105.908	65.0231	55.6388	40.5238	75.5233	-0.89516	0.02174	0.66333	*2610027K06Rik*	11
ENSMUSG00000028309	4755.92	2407.83	3740.96	2171.64	2369.95	2728.99	3634.9	2423.52	0.58465	0.02175	0.66333	*Rnf20*	4
ENSMUSG00000036825	606.206	632.798	961.513	2003.86	1138.39	669.46	733.506	1270.57	-0.79256	0.0218	0.66397	*Ssx2ip*	3
ENSMUSG00000024965	307.688	252.366	226.962	700.46	670.611	157.942	262.338	509.671	-0.95738	0.02189	0.66499	*Fermt3*	19
ENSMUSG00000042535	909.819	1052.78	787.099	1462.79	1460.59	1010.47	916.566	1311.28	-0.51605	0.0219	0.66499	*Gtpbp1*	15
ENSMUSG00000102813	24.452	28.2499	29.069	59.7698	46.5837	50.2544	27.257	52.2026	-0.93682	0.02206	0.66878	*Gm37795*	4
ENSMUSG00000079509	2731.49	1507.6	2842.05	1464.88	1507.18	1748.13	2360.38	1573.4	0.58484	0.02212	0.66972	*Zfx*	X
ENSMUSG00000091698	1.01883	0	2.23608	7.34015	1.94099	19.7428	1.08497	9.67464	-3.20676	0.02229	0.67311	*Gm6526*	14
ENSMUSG00000026614	109.015	140.308	235.906	1012.94	521.155	147.174	161.743	560.423	-1.79296	0.0223	0.67311	*Slc30a10*	1
ENSMUSG00000107770	44.8287	48.9665	45.8396	17.8261	31.0558	19.7428	46.5449	22.8749	1.02438	0.02238	0.67456	*Gm44126*	6
ENSMUSG00000015697	7190.93	3526.53	4767.31	2655.04	3280.27	4084.96	5161.59	3340.09	0.62781	0.02251	0.6774	*Setdb1*	3
ENSMUSG00000025728	1488.52	1649.79	2373.59	4680.92	2867.81	1686.21	1837.3	3078.31	-0.74453	0.02254	0.6774	*Pigq*	17
ENSMUSG00000040124	624.545	384.199	676.413	334.501	333.85	438.828	561.719	369.06	0.60469	0.02257	0.6774	*Gorab*	1
ENSMUSG00000026425	3382.53	1840.95	2939.32	1684.04	1855.58	2013.76	2720.93	1851.13	0.55548	0.02262	0.67742	*Srgap2*	1
ENSMUSG00000047260	1074.87	870.097	1184	691.023	744.369	829.197	1042.99	754.863	0.46574	0.02272	0.67742	*Emc6*	11
ENSMUSG00000001416	7854.19	5658.46	7167.74	4344.32	4896.14	5724.51	6893.46	4988.32	0.46657	0.02273	0.67742	*Cct3*	3
ENSMUSG00000086184	16.3013	9.41664	7.82627	1.04859	2.91148	2.6922	11.1814	2.21742	2.32242	0.02277	0.67742	*Gm12764*	7

ENSMUSG00000074646	70.2995	60.2665	58.138	96.4706	101.902	106.791	62.9013	101.721	-0.69358	0.02278	0.67742	*6430550D23Rik*	2
ENSMUSG00000066009	25.4708	7.53331	25.7149	2.09719	9.70494	3.5896	19.573	5.13057	1.92371	0.02282	0.67742	*Zfp987*	4
ENSMUSG00000030683	163.013	121.475	122.984	74.4501	70.846	104.996	135.824	83.4306	0.7009	0.02282	0.67742	*Sez6l2*	7
ENSMUSG00000025584	1203.24	696.831	1302.51	658.517	626.939	826.505	1067.53	703.987	0.59997	0.02287	0.67742	*Pde8a*	7
ENSMUSG00000004880	1063.66	892.697	1416.55	3095.45	1885.67	916.245	1124.3	1965.79	-0.80604	0.02289	0.67742	*Lbr*	1
ENSMUSG00000022236	41.7722	27.3082	38.0133	54.5268	65.9936	76.279	35.6979	65.5998	-0.88257	0.0229	0.67742	*Ropn1l*	15
ENSMUSG00000034906	688.732	615.848	736.787	1435.52	949.143	716.125	680.456	1033.6	-0.60283	0.02298	0.67872	*Ncaph*	2
ENSMUSG00000087294	23.4332	4.70832	5.59019	0	0.97049	3.5896	11.2439	1.52003	2.84837	0.02303	0.67872	*Gm13556*	2
ENSMUSG00000103966	14.2637	29.1916	26.8329	11.5345	4.85247	8.07659	23.4294	8.15453	1.52826	0.02307	0.67872	*Gm37120*	1
ENSMUSG00000061232	5318.31	2353.22	4561.6	1342.2	2293.28	3454.99	4077.71	2363.49	0.78662	0.02307	0.67872	*H2-K1*	17
ENSMUSG00000085881	7.13184	15.0666	22.3608	2.09719	1.94099	6.2818	14.8531	3.43999	2.08956	0.0232	0.67965	*Gm15912*	13
ENSMUSG00000026429	699.939	366.307	832.938	410	324.145	448.7	633.061	394.282	0.68224	0.02322	0.67965	*Ube2t*	1
ENSMUSG00000029695	42.791	92.283	59.256	109.054	95.1084	141.789	64.7767	115.317	-0.82993	0.02323	0.67965	*Aass*	6
ENSMUSG00000033948	342.328	175.149	422.618	118.491	157.22	253.067	313.365	176.259	0.82742	0.02324	0.67965	*Zswim5*	4
ENSMUSG00000022054	21.3955	27.3082	24.5968	54.5268	41.7312	47.5622	24.4335	47.9401	-0.97025	0.02328	0.68003	*Nefm*	14
ENSMUSG00000021385	610.282	304.157	581.38	235.933	316.381	376.908	498.606	309.741	0.68535	0.02341	0.68283	*Ippk*	13
ENSMUSG00000024293	1098.3	703.423	1444.51	678.44	629.85	813.941	1082.08	707.411	0.61249	0.02346	0.68319	*Esco1*	18
ENSMUSG00000035984	45.8475	53.6748	31.3051	50.3325	79.5805	125.636	43.6091	85.183	-0.96528	0.02352	0.68405	*Nme5*	18

ENSMUSG00000021981	1349.95	790.997	1688.24	847.263	759.897	894.707	1276.4	833.956	0.61355	0.02355	0.68405	*Cab39l*	14
ENSMUSG00000066224	15.2825	3.76665	15.6525	0	1.94099	3.5896	11.5672	1.84353	2.61298	0.02359	0.68405	*Arid3c*	4
ENSMUSG00000076441	53.9982	87.5747	93.9152	102.762	175.659	124.739	78.496	134.387	-0.77698	0.02362	0.68405	*Ass1*	2
ENSMUSG00000040310	20.3767	29.1916	15.6525	58.7212	29.1148	53.844	21.7403	47.2267	-1.11234	0.02369	0.68483	*Alx4*	2
ENSMUSG00000038718	3439.58	2217.62	3320.57	2074.12	1928.37	2345.8	2992.59	2116.1	0.49979	0.02371	0.68483	*Pbx3*	2
ENSMUSG00000097138	0	0	0	2.09719	3.88198	7.1792	0	4.38612	-4.5466	0.02375	1	*Gm26625*	6
ENSMUSG00000048916	13.2448	15.0666	7.82627	17.8261	55.3181	24.2298	12.0459	32.458	-1.42415	0.02376	0.68487	*Gm5540*	3
ENSMUSG00000045896	2869.04	1873.91	2851	1509.97	1683.81	2081.07	2531.31	1758.28	0.52539	0.02379	0.68487	*Paip2b*	6
ENSMUSG00000107856	0	0	0	2.09719	0	15.2558	0	5.78433	-4.94662	0.02381	1	*Gm7838*	6
ENSMUSG00000048442	42.791	64.9748	72.6725	219.156	154.309	34.1012	60.1461	135.855	-1.17418	0.02382	0.68487	*Smim5*	11
ENSMUSG00000044285	184.409	201.516	148.699	207.621	175.659	651.512	178.208	344.931	-0.95325	0.02385	0.68487	*Gm1821*	14
ENSMUSG00000028232	605.187	457.649	633.928	393.222	400.814	418.188	565.588	404.075	0.48427	0.02392	0.68487	*Tmem68*	4
ENSMUSG00000066839	801.822	442.582	714.426	282.072	404.696	540.234	652.944	409.001	0.67359	0.02398	0.68487	*Ecsit*	9
ENSMUSG00000009863	2116.12	1711	2471.98	1459.64	1446.04	1670.96	2099.7	1525.55	0.46051	0.02398	0.68487	*Sdhb*	4
ENSMUSG00000046958	21.3955	2.82499	3.35411	1.04859	0	1.7948	9.19154	0.9478	3.26276	0.02399	0.68487	*4930432E11Rik*	7
ENSMUSG00000029047	412.628	305.099	317.523	206.573	273.679	229.734	345.083	236.662	0.54371	0.02402	0.68487	*Pex10*	4
ENSMUSG00000069516	267.953	480.248	316.405	944.782	433.811	432.547	354.869	603.713	-0.76533	0.02407	0.68487	*Lyz2*	10
ENSMUSG00000091509	428.929	389.849	640.636	332.404	296.001	356.268	486.471	328.224	0.56648	0.02407	0.68487	*Gm17066*	14

ENSMUSG00000070000	261.84	259.899	298.516	545.268	500.775	254.861	273.419	433.635	-0.66483	0.02414	0.6858	*Fcho1*	8
ENSMUSG00000032839	499.229	189.274	709.954	145.754	218.361	358.96	466.152	241.025	0.94979	0.02425	0.68734	*Trpc1*	9
ENSMUSG00000081998	93.7327	90.3997	79.3807	53.4783	63.0821	35.896	87.8377	50.8188	0.79286	0.02429	0.68734	*Gm13245*	4
ENSMUSG00000100175	1.01883	1.88333	0	2.09719	11.6459	12.5636	0.96739	8.7689	-3.14174	0.02429	0.68734	*1700025M24Rik*	5
ENSMUSG00000049281	26.4897	35.7832	20.1247	41.9437	47.5542	78.0738	27.4659	55.8572	-1.02164	0.02437	0.68867	*Scn3b*	9
ENSMUSG00000022519	796.728	529.215	665.233	419.437	465.837	513.312	663.725	466.196	0.50891	0.02441	0.68881	*Srl*	16
ENSMUSG00000041528	765.144	830.547	1103.5	2038.47	1231.56	955.73	899.732	1408.58	-0.6466	0.02445	0.68895	*Rnf123*	9
ENSMUSG00000034799	886.386	276.849	449.451	182.455	253.299	428.06	537.562	287.938	0.89958	0.02449	0.68899	*Unc13a*	8
ENSMUSG00000115249	89.6574	44.2582	60.3741	24.1176	19.4099	48.4596	64.7632	30.6624	1.07245	0.02456	0.69001	*Gm49085*	15
ENSMUSG00000022385	471.72	270.257	364.48	552.609	563.857	507.928	368.819	541.465	-0.5544	0.02459	0.69001	*Gtse1*	15
ENSMUSG00000034998	389.195	339.941	431.563	570.435	527.949	498.954	386.899	532.446	-0.46121	0.02468	0.69175	*Foxn2*	17
ENSMUSG00000032375	465.607	451.999	651.816	370.153	331.909	393.958	523.141	365.34	0.51695	0.02475	0.69274	*Aph1b*	9
ENSMUSG00000063851	57.0547	8.47497	39.1313	4.19437	11.6459	17.0506	34.887	10.9636	1.65816	0.02482	0.69308	*Rnf183*	4
ENSMUSG00000074934	29.5462	3.76665	20.1247	6.29156	1.94099	4.487	17.8125	4.23985	2.07228	0.02483	0.69308	*Grem1*	2
ENSMUSG00000008845	0	0	0	4.19437	4.85247	3.5896	0	4.21215	-4.4829	0.02483	1	*Cd163*	6
ENSMUSG00000034121	252.671	258.957	191.185	359.667	357.142	297.039	234.271	337.949	-0.52679	0.02494	0.69518	*Mks1*	11
ENSMUSG00000035031	35.6592	5.64998	12.2984	8.38875	0	0	17.8692	2.79625	2.70894	0.025	0.69567	*C8a*	4
ENSMUSG00000093954	8.15067	27.3082	20.1247	88.0818	31.0558	26.0246	18.5279	48.3874	-1.37855	0.02503	0.69567	*Gm16867*	14

ENSMUSG00000049493	151.806	60.2665	242.614	45.0895	71.8165	103.201	151.562	73.369	1.042	0.02507	0.69568	*Pls1*	9
ENSMUSG00000030450	2.03767	8.47497	5.59019	27.2634	9.70494	19.7428	5.36761	18.9037	-1.8074	0.0251	0.69568	*Oca2*	7
ENSMUSG00000011349	1075.89	619.615	1170.59	276.829	438.663	889.323	955.363	534.938	0.83566	0.02513	0.69568	*Dmrtc2*	7
ENSMUSG00000035757	420.778	356.891	373.425	275.78	289.207	269.22	383.698	278.069	0.46451	0.02526	0.69718	*Selenoo*	15
ENSMUSG00000036875	424.854	286.266	363.362	572.532	713.313	372.421	358.161	552.755	-0.62602	0.02527	0.69718	*Dna2*	10
ENSMUSG00000055069	88.6386	41.4332	120.748	25.1662	43.6722	52.9466	83.6066	40.595	1.03527	0.02528	0.69718	*Rab39*	9
ENSMUSG00000097325	61.13	49.9082	73.7905	32.5064	26.2033	40.383	61.6096	33.0309	0.89476	0.02534	0.69794	*Gm16897*	1
ENSMUSG00000034164	140.599	256.133	133.047	330.307	263.004	255.759	176.593	283.023	-0.67758	0.02544	0.69957	*Emid1*	11
ENSMUSG00000046213	13.2448	6.59165	22.3608	4.19437	0.97049	4.487	14.0657	3.21729	2.12057	0.02557	0.70044	*Cym*	3
ENSMUSG00000058589	9.16951	11.3	17.8886	4.19437	36.8788	87.9451	12.786	43.0061	-1.75771	0.02561	0.70044	*Anks1b*	10
ENSMUSG00000033282	1309.2	731.673	1361.77	613.427	736.605	888.425	1134.21	746.152	0.60343	0.02563	0.70044	*Rpgrip1l*	8
ENSMUSG00000028879	2208.83	1880.5	2589.38	1621.13	1586.76	1745.44	2226.24	1651.11	0.43088	0.02566	0.70044	*Stx12*	4
ENSMUSG00000018909	1206.3	1440.75	1566.37	4009.82	2407.8	1011.37	1404.47	2476.33	-0.81802	0.02571	0.70044	*Arrb1*	7
ENSMUSG00000048191	187.465	110.175	137.519	823.146	528.919	107.688	145.053	486.584	-1.74596	0.02572	0.70044	*Muc6*	7
ENSMUSG00000060038	481.908	523.565	475.166	351.279	397.902	338.32	493.547	362.5	0.44561	0.02577	0.70044	*Dhps*	8
ENSMUSG00000037419	403.458	541.457	642.872	1247.83	818.126	512.415	529.262	859.456	-0.69917	0.02577	0.70044	*Endod1*	9
ENSMUSG00000028031	11.2072	5.64998	7.82627	25.1662	19.4099	22.435	8.22781	22.337	-1.44355	0.02577	0.70044	*Dkk2*	3
ENSMUSG00000029110	4320.87	2921.04	5031.17	2935.01	2628.1	3049.36	4091.03	2870.82	0.51085	0.02581	0.70054	*Rnf4*	5

ENSMUSG00000025963	4.07534	0	3.35411	9.43734	9.70494	17.948	2.47648	12.3634	-2.34627	0.0259	0.70125	*Mdh1b*	1
ENSMUSG00000053799	208.861	186.449	191.185	467.673	356.171	166.916	195.498	330.253	-0.75523	0.02592	0.70125	*Exoc6*	19
ENSMUSG00000040963	0	1.88333	0	6.29156	5.82296	9.87139	0.62778	7.32864	-3.47349	0.02595	0.70125	*Asgr2*	11
ENSMUSG00000015342	234.332	250.483	579.144	1031.82	607.529	343.704	354.653	661.016	-0.89877	0.02597	0.70125	*Xk*	X
ENSMUSG00000105572	225.162	43.3165	167.706	37.7494	91.2264	69.9972	145.395	66.3243	1.12967	0.02606	0.70203	*Gm43300*	3
ENSMUSG00000081150	2.03767	3.76665	6.70823	0	0	0	4.17085	0	4.54299	0.02606	1	*Gm12684*	4
ENSMUSG00000044737	17.3202	1.88333	8.9443	1.04859	1.94099	0.8974	9.3826	1.29566	2.85509	0.02611	0.70203	*Klk14*	7
ENSMUSG00000068117	641.865	264.607	602.623	141.56	217.391	446.008	503.032	268.319	0.90497	0.02611	0.70203	*Mei1*	15
ENSMUSG00000017664	1136	933.189	1121.39	752.89	815.215	822.018	1063.53	796.708	0.41631	0.02614	0.70203	*Slc35c2*	2
ENSMUSG00000056211	4571.51	2666.79	4007.05	2444.27	2560.16	2880.65	3748.45	2628.36	0.51197	0.02617	0.70203	*R3hdm1*	1
ENSMUSG00000022021	359.648	311.691	476.284	857.749	500.775	442.418	382.541	600.314	-0.65019	0.02624	0.70303	*Diaph3*	14
ENSMUSG00000051435	4.07534	4.70832	4.47215	8.38875	10.6754	30.5116	4.4186	16.5253	-1.90882	0.02629	0.70357	*Fhad1*	4
ENSMUSG00000068151	0	0	0	1.04859	6.79346	5.3844	0	4.40882	-4.55316	0.02639	1	*A230006K03Rik*	7
ENSMUSG00000031256	3808.4	1925.7	4250.78	1686.14	2118.59	2575.54	3328.29	2126.75	0.64587	0.02648	0.70754	*Cstf2*	X
ENSMUSG00000071267	827.293	572.531	1052.07	564.143	452.25	624.59	817.3	546.994	0.57857	0.02656	0.70774	*Zfp942*	17
ENSMUSG00000015970	17.3202	20.7166	10.0623	41.9437	32.9968	32.3064	16.033	35.749	-1.14694	0.02665	0.70774	*Chdh*	14
ENSMUSG00000049036	105.959	119.591	109.568	69.2071	77.6395	67.305	111.706	71.3839	0.64677	0.02669	0.70774	*Tmem121*	12
ENSMUSG00000081803	0	0	0	6.29156	1.94099	4.487	0	4.23985	-4.49114	0.02669	1	*Gm15282*	X

ENSMUSG00000106791	16.3013	4.70832	13.4165	3.14578	2.91148	0.8974	11.4754	2.31822	2.31937	0.02669	0.70774	*4930553P18Rik*	6
ENSMUSG00000003198	244.52	183.624	260.503	160.435	140.722	164.224	229.549	155.127	0.56392	0.0267	0.70774	*Zfp959*	17
ENSMUSG00000091387	86.6009	37.6665	86.0889	22.0205	32.9968	47.5622	70.1188	34.1931	1.02852	0.02671	0.70774	*Gcnt4*	13
ENSMUSG00000019359	497.191	489.665	442.743	348.133	363.935	346.396	476.533	352.821	0.43399	0.02672	0.70774	*Gdpd2*	X
ENSMUSG00000102308	22.4143	0.94166	2.23608	1.04859	0.97049	0	8.5307	0.67303	3.69651	0.02679	0.7079	*2310046K23Rik*	3
ENSMUSG00000111353	44.8287	18.8333	45.8396	10.4859	18.4394	17.0506	36.5005	15.3253	1.24388	0.0268	0.7079	*Gm19324*	9
ENSMUSG00000037012	831.368	977.447	575.79	1754.3	1350.93	740.355	794.868	1281.86	-0.68874	0.02687	0.70831	*Hk1*	10
ENSMUSG00000037416	2861.9	1224.16	3312.75	1506.83	1439.24	1653.01	2466.27	1533.03	0.68571	0.0269	0.70831	*Dmxl1*	18
ENSMUSG00000115026	28.5274	30.1332	36.8953	11.5345	19.4099	10.7688	31.8519	13.9044	1.19487	0.02691	0.70831	*Gm49041*	6
ENSMUSG00000039414	2148.72	1071.61	1653.58	817.903	1018.05	1329.95	1624.64	1055.3	0.62203	0.02696	0.70859	*Heatr5b*	17
ENSMUSG00000088327	0	0.94166	0	5.24297	7.76395	3.5896	0.31389	5.53217	-3.91359	0.02699	1	*Gm25666*	5
ENSMUSG00000028730	42.791	29.1916	19.0066	50.3325	77.6395	52.0492	30.3297	60.007	-0.98106	0.02705	0.71019	*Cfap57*	4
ENSMUSG00000038860	1326.52	874.806	1243.26	631.253	658.965	1024.83	1148.2	771.683	0.57257	0.02716	0.71025	*Garnl3*	2
ENSMUSG00000037544	488.021	478.365	614.921	1145.06	728.841	552.798	527.103	808.901	-0.61767	0.02719	0.71025	*Dlgap5*	14
ENSMUSG00000074805	103.921	29.1916	73.7905	10.4859	24.2623	49.357	68.9677	28.0351	1.2902	0.02719	0.71025	*Il1bos*	2
ENSMUSG00000030763	1075.89	775.931	1120.27	722.481	643.437	768.174	990.698	711.364	0.47733	0.02722	0.71025	*Lcmt1*	7
ENSMUSG00000042406	1537.42	2321.2	1682.65	3821.07	2483.49	2045.17	1847.09	2783.25	-0.59121	0.02723	0.71025	*Atf4*	15
ENSMUSG00000079235	8.15067	15.0666	15.6525	15.7289	32.9968	50.2544	12.9566	32.9934	-1.35406	0.02732	0.71175	*Ccdc13*	9

ENSMUSG00000029119	448.287	607.373	354.418	728.772	690.992	623.693	470.026	681.152	-0.53391	0.02739	0.71175	*Man2b2*	5
ENSMUSG00000037369	3814.51	2012.34	4228.42	2176.88	2239.9	2287.47	3351.76	2234.75	0.58463	0.02742	0.71175	*Kdm6a*	X
ENSMUSG00000113047	116.147	65.9165	91.6791	91.2276	205.745	181.275	91.2475	159.416	-0.8075	0.02746	0.71175	*Gm47469*	13
ENSMUSG00000064193	69.2807	88.5164	48.0756	80.7417	109.666	731.381	68.6242	307.263	-2.16252	0.02751	0.71175	*Gm4735*	2
ENSMUSG00000097583	81.5067	18.8333	46.9576	10.4859	15.5279	31.409	49.0992	19.1409	1.35017	0.02752	0.71175	*6430590A07Rik*	3
ENSMUSG00000027333	377.987	640.331	716.662	1622.17	800.657	574.336	578.327	999.056	-0.7884	0.02757	0.71175	*Smox*	2
ENSMUSG00000002658	1331.62	1330.57	695.42	1321.23	1816.76	2001.2	1119.2	1713.06	-0.61386	0.02758	0.71175	*Gtf2f1*	17
ENSMUSG00000106422	1.01883	0.94166	0	2.09719	12.6164	7.1792	0.6535	7.2976	-3.4471	0.02759	0.71175	*Gm42812*	3
ENSMUSG00000078864	8.15067	13.1833	10.0623	31.4578	27.1738	19.7428	10.4654	26.1248	-1.31314	0.02771	0.7138	*Gm14322*	2
ENSMUSG00000108841	12.226	4.70832	3.35411	6.29156	21.3509	44.87	6.76281	24.1708	-1.83973	0.02774	0.71382	*Frmpd2*	14
ENSMUSG00000053580	2106.95	1012.29	2202.54	832.583	1176.24	1351.48	1773.92	1120.1	0.66285	0.02784	0.71455	*Tanc2*	11
ENSMUSG00000032218	806.916	807.006	1194.06	1927.31	1358.69	990.729	935.996	1425.58	-0.60702	0.02784	0.71455	*Ccnb2*	9
ENSMUSG00000000346	5869.5	3371.16	4820.98	3421.56	3052.2	3449.6	4687.21	3307.79	0.50279	0.02796	0.71599	*Dazap2*	15
ENSMUSG00000081044	52.9794	30.1332	128.574	10.4859	21.3509	50.2544	70.5623	27.3637	1.35668	0.02797	0.71599	*AU015836*	X
ENSMUSG00000113505	3.0565	1.88333	12.2984	0	0.97049	0	5.74608	0.3235	4.03798	0.02798	1	*Gm48593*	13
ENSMUSG00000116777	40.7534	9.41664	16.7706	5.24297	10.6754	4.487	22.3135	6.8018	1.71394	0.02806	0.71748	*AC078895.2*	16
ENSMUSG00000023943	3.0565	0	0	2.09719	7.76395	18.8454	1.01883	9.56884	-3.23395	0.02813	0.71781	*Sult1c1*	17
ENSMUSG00000031766	16.3013	4.70832	14.5345	2.09719	1.94099	3.5896	11.8481	2.54259	2.20591	0.02817	0.71781	*Slc12a3*	8

ENSMUSG00000114438	8.15067	9.41664	14.5345	23.069	41.7312	17.948	10.7006	27.5828	-1.37156	0.02819	0.71781	*Gm47827*	13
ENSMUSG00000085196	4.07534	0	7.82627	16.7775	8.73444	25.1272	3.9672	16.8797	-2.11882	0.02821	0.71781	*Gm14963*	19
ENSMUSG00000093806	4.07534	0.94166	7.82627	0	0	0	4.28109	0	4.57716	0.02824	1	*Asmt*	X
ENSMUSG00000107634	16.3013	11.3	19.0066	8.38875	0	0	15.536	2.79625	2.50968	0.02826	0.71821	*Gm36816*	6
ENSMUSG00000000706	28.5274	48.9665	76.0266	247.468	102.872	27.8194	51.1735	126.053	-1.30038	0.0284	0.72086	*Btn1a1*	13
ENSMUSG00000035299	82.5255	45.1999	54.7839	98.5678	108.695	98.7139	60.8364	101.992	-0.74642	0.02847	0.72168	*Mid1*	X
ENSMUSG00000042333	90.6762	102.641	160.997	663.759	455.162	83.4581	118.105	400.793	-1.76291	0.02856	0.72168	*Tnfrsf14*	4
ENSMUSG00000114357	0	0	0	3.14578	7.76395	1.7948	0	4.23484	-4.49006	0.02858	1	*Gm48449*	14
ENSMUSG00000030269	1019.85	813.597	951.45	662.711	652.172	756.508	928.3	690.463	0.42654	0.02859	0.72168	*Mtmr14*	6
ENSMUSG00000036352	1487.5	1712.89	2084.02	4573.96	2523.28	1570.45	1761.47	2889.23	-0.71381	0.0287	0.72168	*Ubac1*	2
ENSMUSG00000030879	1213.43	1274.07	1589.85	983.58	923.91	1108.29	1359.12	1005.26	0.43468	0.02871	0.72168	*Mrpl17*	7
ENSMUSG00000027806	2533.84	1439.8	2711.24	1345.35	1379.07	1775.95	2228.3	1500.12	0.5705	0.02874	0.72168	*Tsc22d2*	3
ENSMUSG00000040929	914.913	422.807	1007.35	361.765	535.713	551.003	781.691	482.827	0.69414	0.02875	0.72168	*Rfx3*	19
ENSMUSG00000022157	0	1.88333	30.187	2.09719	0	0	10.6901	0.69906	3.982	0.02879	0.72168	*Mcpt8*	14
ENSMUSG00000026991	673.449	667.64	673.059	811.611	880.238	967.397	671.383	886.415	-0.40117	0.02881	0.72168	*Pkp4*	2
ENSMUSG00000059187	18.339	4.70832	32.4231	0	0	8.97399	18.4901	2.99133	2.59329	0.02883	0.72168	*Fam19a1*	6
ENSMUSG00000065968	63.1677	48.9665	41.3674	22.0205	19.4099	35.896	51.1672	25.7754	0.98544	0.02885	0.72168	*Ifitm7*	16
ENSMUSG00000040482	813.029	812.656	1007.35	624.962	656.054	682.024	877.679	654.346	0.42305	0.02885	0.72168	*Dxo*	17

ENSMUSG00000037798	1.01883	0	0	2.09719	4.85247	9.87139	0.33961	5.60702	-3.94061	0.02895	1	*Mat1a*	14
ENSMUSG00000031872	47.8852	36.7249	39.1313	71.3043	71.8165	68.2024	41.2471	70.4411	-0.7723	0.02921	0.7298	*Bean1*	8
ENSMUSG00000030691	2384.07	1257.12	2514.47	1106.27	1213.12	1689.8	2051.89	1336.4	0.61819	0.02926	0.73013	*Fchsd2*	7
ENSMUSG00000101258	12.226	14.125	5.59019	36.7008	31.0558	16.1532	10.6471	27.9699	-1.37968	0.02935	0.73154	*Gm29477*	11
ENSMUSG00000029686	5165.49	3852.35	5100.49	3498.11	3448.16	3664.08	4706.11	3536.78	0.41199	0.02946	0.73335	*Cul1*	6
ENSMUSG00000055044	152.825	113	106.214	219.156	278.532	122.046	124.013	206.578	-0.73486	0.02957	0.73423	*Pdlim1*	19
ENSMUSG00000022429	2225.13	895.522	1855.94	529.54	856.946	1460.97	1658.87	949.151	0.80497	0.02959	0.73423	*Dmc1*	15
ENSMUSG00000021061	431.986	823.956	1683.77	6280.02	3175.46	969.191	979.902	3474.89	-1.82631	0.0296	0.73423	*Sptb*	12
ENSMUSG00000030677	946.497	888.93	912.319	1564.5	1178.18	1041.88	915.915	1261.52	-0.46154	0.02971	0.73601	*Kif22*	7
ENSMUSG00000074274	1.01883	0	0	12.5831	7.76395	0	0.33961	6.78236	-4.20329	0.02974	1	*D930028M14Rik*	7
ENSMUSG00000110649	21.3955	25.4249	38.0133	46.1381	64.0526	51.1518	28.2779	53.7808	-0.93293	0.02983	0.73818	*Gm40466*	8
ENSMUSG00000099418	25.4708	2.82499	23.4788	8.38875	0	0.8974	17.2582	3.09538	2.50267	0.02991	0.73845	*Gm6657*	12
ENSMUSG00000022404	1629.12	1233.58	1696.06	1102.07	968.553	1250.97	1519.59	1107.2	0.45637	0.02998	0.73845	*Slc25a17*	15
ENSMUSG00000070730	473.758	429.399	498.645	610.281	651.201	604.847	467.267	622.11	-0.41341	0.03007	0.73845	*Rmdn3*	2
ENSMUSG00000028577	2825.23	1926.64	3032.12	1544.58	1713.89	2197.73	2594.66	1818.73	0.51227	0.03008	0.73845	*Plaa*	4
ENSMUSG00000039563	94.7516	39.5499	49.1937	18.8747	42.7017	26.0246	61.165	29.2003	1.0646	0.03009	0.73845	*2210406O10Rik*	5
ENSMUSG00000031844	21.3955	25.4249	13.4165	37.7494	51.4362	35.896	20.079	41.6938	-1.04697	0.03014	0.73845	*Hsd17b2*	8
ENSMUSG00000017499	440.136	459.532	521.006	1080.05	962.73	346.396	473.558	796.392	-0.74959	0.03016	0.73845	*Cdc6*	11

ENSMUSG00000029192	2229.21	1642.26	2624.04	1625.32	1564.44	1505.84	2165.17	1565.2	0.46792	0.03018	0.73845	*Tbc1d14*	5
ENSMUSG00000112909	5.09417	6.59165	10.0623	1.04859	0.97049	0.8974	7.24939	0.97216	2.89848	0.0302	0.73845	*Gm10120*	10
ENSMUSG00000067338	49.9229	60.2665	175.532	35.6522	21.3509	69.0998	95.2404	42.0343	1.17415	0.03026	0.73845	*Tuba3b*	6
ENSMUSG00000028684	1454.89	2088.61	2175.7	5269.18	2866.84	1563.27	1906.4	3233.1	-0.76194	0.03029	0.73845	*Urod*	4
ENSMUSG00000074582	1920.5	977.447	1986.75	1079	1065.6	1122.65	1628.23	1089.08	0.57987	0.0303	0.73845	*Arfgef2*	2
ENSMUSG00000107251	0	4.70832	0	11.5345	4.85247	17.0506	1.56944	11.1459	-2.7789	0.03036	0.73845	*Gm43102*	5
ENSMUSG00000068740	185.428	263.666	173.296	268.44	349.378	299.731	207.463	305.85	-0.55847	0.03039	0.73845	*Celsr2*	3
ENSMUSG00000075289	272.029	210.933	195.657	102.762	157.22	178.582	226.206	146.188	0.628	0.03039	0.73845	*Carns1*	19
ENSMUSG00000030609	465.607	286.266	301.87	318.772	878.297	1801.98	351.248	999.682	-1.50914	0.03041	0.73845	*Aen*	7
ENSMUSG00000080824	142.637	62.1498	190.066	55.5754	60.1706	94.2269	131.618	69.991	0.9064	0.03048	0.73849	*Gm9001*	6
ENSMUSG00000028633	1515.01	1226.99	1834.7	978.337	1097.63	1232.13	1525.56	1102.7	0.46775	0.03048	0.73849	*Ctps*	4
ENSMUSG00000089563	10.1883	4.70832	19.0066	3.14578	2.91148	0.8974	11.3011	2.31822	2.2937	0.03059	0.73954	*Gm24667*	16
ENSMUSG00000047495	73.356	18.8333	52.5478	6.29156	17.4689	31.409	48.2457	18.3898	1.38085	0.03063	0.73954	*Dlgap2*	8
ENSMUSG00000024672	30.565	19.7749	17.8886	72.3529	39.7902	32.3064	22.7429	48.1499	-1.07716	0.03063	0.73954	*Ms4a7*	19
ENSMUSG00000026809	13.2448	27.3082	19.0066	45.0895	43.6722	34.9986	19.8532	41.2534	-1.04933	0.03069	0.73996	*Spaca9*	2
ENSMUSG00000096433	231.275	179.858	301.87	142.609	145.574	181.275	237.668	156.486	0.60022	0.03078	0.73996	*Zfp994*	17
ENSMUSG00000003847	10131.3	7263.05	9284.19	6961.61	6501.34	6602.17	8892.84	6688.37	0.41096	0.0308	0.73996	*Nfat5*	8
ENSMUSG00000084796	20.3767	15.0666	6.70823	60.8184	74.728	3.5896	14.0505	46.3787	-1.71659	0.03081	0.73996	*Mir142hg*	11

ENSMUSG00000078735	1.01883	1.88333	1.11804	3.14578	17.4689	6.2818	1.34007	8.96549	-2.73227	0.03083	0.73996	*Il11ra2*	4
ENSMUSG00000028212	1490.55	673.289	1891.72	878.721	678.375	941.372	1351.85	832.823	0.69845	0.0309	0.74062	*Ccne2*	4
ENSMUSG00000004071	2863.94	2925.75	2559.19	2145.42	2135.09	2217.47	2782.96	2165.99	0.36167	0.03094	0.74062	*Cdip1*	16
ENSMUSG00000044177	75.3937	102.641	54.7839	138.414	106.754	136.405	77.6063	127.191	-0.70853	0.03096	0.74062	*Wfikkn2*	11
ENSMUSG00000044201	118.185	140.308	133.047	262.148	165.954	170.506	130.513	199.536	-0.61071	0.03101	0.74093	*Cdc25c*	18
ENSMUSG00000039253	315.839	553.698	447.215	1160.79	726.9	367.036	438.917	751.576	-0.77521	0.03125	0.74575	*Fn3krp*	11
ENSMUSG00000036403	423.835	341.824	365.598	571.483	529.89	452.289	377.086	517.887	-0.45741	0.03136	0.7475	*Cep135*	5
ENSMUSG00000056073	33.6215	16.0083	24.5968	46.1381	38.8198	63.7154	24.7422	49.5577	-1.00703	0.03145	0.74838	*Grik2*	10
ENSMUSG00000030433	13.2448	20.7166	16.7706	38.7979	51.4362	22.435	16.9107	37.5564	-1.14654	0.03147	0.74838	*Sbk2*	7
ENSMUSG00000033688	158.938	80.9831	175.532	457.187	234.86	111.278	138.484	267.775	-0.95146	0.03152	0.74879	*1300017J02 Rik*	9
ENSMUSG00000116607	0	0	1.11804	2.09719	8.73444	5.3844	0.37268	5.40534	-3.88443	0.03162	1	*AC134560. 2*	16
ENSMUSG00000024406	27.5085	37.6665	98.3874	17.8261	3.88198	36.7934	54.5208	19.5005	1.47549	0.03163	0.74986	*Pou5f1*	17
ENSMUSG00000029920	1948.01	1543.39	2305.39	1423.99	1289.79	1526.48	1932.26	1413.42	0.45077	0.03164	0.74986	*Smarcad1*	6
ENSMUSG00000029822	383.082	320.166	339.884	264.245	268.827	216.273	347.71	249.782	0.47783	0.0317	0.7504	*Osbpl3*	6
ENSMUSG00000004508	1816.58	1314.56	1823.52	1082.15	1075.31	1415.2	1651.55	1190.88	0.47133	0.03173	0.7504	*Gab2*	7
ENSMUSG00000029104	4548.07	1718.54	3391.01	1563.45	2084.62	2405.93	3219.21	2018	0.67359	0.03187	0.75282	*Htt*	5
ENSMUSG00000031604	852.764	762.748	912.319	731.918	578.414	549.208	842.61	619.847	0.44323	0.03191	0.75292	*Msmo1*	8
ENSMUSG00000004098	28.5274	32.9582	26.8329	68.1586	42.7017	52.9466	29.4395	54.6023	-0.88734	0.03207	0.75568	*Col5a3*	9

ENSMUSG00000037926	946.497	677.998	1271.21	626.01	712.342	656.896	965.235	665.083	0.53668	0.03211	0.75575	*Ssh2*	11
ENSMUSG00000033416	726.429	931.305	1008.47	1953.53	1219.91	897.399	888.735	1356.95	-0.61027	0.03228	0.75859	*Gucd1*	10
ENSMUSG00000062729	1342.82	1958.66	2028.12	4462.81	2818.31	1475.32	1776.53	2918.82	-0.71618	0.0323	0.75859	*Ppox*	1
ENSMUSG00000020279	71.3184	26.3666	22.3608	33.555	98.9904	139.994	40.0152	90.8466	-1.1842	0.03252	0.76189	*Il9r*	11
ENSMUSG00000045210	2172.15	1331.51	2455.21	1377.85	1315.02	1458.27	1986.29	1383.71	0.52122	0.03253	0.76189	*Vcpip1*	1
ENSMUSG00000029166	366.78	435.99	402.494	438.312	573.562	693.69	401.755	568.521	-0.50144	0.03255	0.76189	*Mapre3*	5
ENSMUSG00000039745	48.904	79.0997	130.81	274.731	133.928	91.5347	86.2714	166.731	-0.95104	0.03264	0.76279	*Htatip2*	7
ENSMUSG00000053040	177.277	146.9	193.421	118.491	102.872	127.431	172.532	116.265	0.56769	0.03266	0.76279	*Aph1c*	9
ENSMUSG00000096906	0	5.64998	13.4165	0	0.97049	0	6.35548	0.3235	4.18767	0.0327	1	*Gm26506*	7
ENSMUSG00000037443	1570.02	863.506	1734.08	734.015	901.589	1113.67	1389.2	916.426	0.59955	0.03274	0.76373	*Cep85*	4
ENSMUSG00000039109	61.13	58.3831	115.158	114.297	108.695	180.377	78.2237	134.456	-0.78689	0.03279	0.76373	*F13a1*	13
ENSMUSG00000035722	319.914	352.182	409.202	926.956	675.464	257.554	360.433	619.991	-0.78207	0.03281	0.76373	*Abca7*	10
ENSMUSG00000026602	44.8287	32.9582	17.8886	51.3811	70.846	61.0232	31.8918	61.0834	-0.93367	0.03293	0.76566	*Nphs2*	1
ENSMUSG00000037379	116.147	152.55	139.755	260.051	165.954	191.146	136.15	205.717	-0.59394	0.03316	0.76847	*Spon2*	5
ENSMUSG00000027737	28.5274	44.2582	13.4165	84.936	47.5542	48.4596	28.734	60.3166	-1.05927	0.03319	0.76847	*Slc7a11*	3
ENSMUSG00000114547	16.3013	20.7166	22.3608	11.5345	127.135	28.7168	19.7929	55.7953	-1.49642	0.0332	0.76847	*Gm3226*	13
ENSMUSG00000043953	13.2448	32.9582	51.4298	169.872	61.1411	21.5376	32.5443	84.1836	-1.37117	0.0332	0.76847	*Ccrl2*	9
ENSMUSG00000001348	233.313	478.365	536.658	2398.13	1099.57	386.779	416.112	1294.83	-1.63766	0.03328	0.76855	*Acp5*	9

ENSMUSG00000034645	319.914	79.0997	212.427	53.4783	97.0494	154.353	203.814	101.627	1.00101	0.03328	0.76855	*Zyg11a*	4
ENSMUSG00000069274	17.3202	6.59165	1.11804	0	0.97049	1.7948	8.34329	0.92176	3.14522	0.03334	0.76892	*Hist1h4f*	13
ENSMUSG00000086540	13.2448	5.64998	13.4165	5.24297	46.5837	46.6648	10.7704	32.8305	-1.61775	0.03337	0.76892	*Scml1*	X
ENSMUSG00000103928	182.371	117.708	160.997	84.936	115.489	96.9191	153.692	99.1147	0.63101	0.03343	0.76954	*Gm37893*	1
ENSMUSG00000086096	0	0	0	1.04859	1.94099	9.87139	0	4.28699	-4.51619	0.03347	1	*Gm12688*	4
ENSMUSG00000056531	1122.75	317.341	868.716	353.376	401.784	573.438	769.604	442.866	0.79648	0.03349	0.76989	*Ccdc18*	5
ENSMUSG00000059891	15.2825	17.8916	11.1804	6.29156	59.2001	59.2284	14.7848	41.5733	-1.49146	0.03364	0.77053	*Tsks*	7
ENSMUSG00000074476	307.688	314.516	342.12	497.033	504.657	350.883	321.441	450.858	-0.48774	0.03369	0.77053	*Spc24*	9
ENSMUSG00000063077	3615.84	2572.63	3511.76	2312.15	2527.17	2405.03	3233.41	2414.78	0.42101	0.03373	0.77053	*Kif1b*	4
ENSMUSG00000004085	269.991	278.732	333.175	537.928	439.634	311.398	293.966	429.653	-0.54716	0.03376	0.77053	*Map3k20*	2
ENSMUSG00000031004	7288.74	4992.7	8548.52	17270.3	9745.7	6507.94	6943.32	11174.7	-0.68653	0.03383	0.77053	*Mki67*	7
ENSMUSG00000024206	360.667	294.741	374.543	574.629	451.28	415.496	343.317	480.468	-0.48496	0.03386	0.77053	*Rfx2*	17
ENSMUSG00000033633	1.01883	0.94166	2.23608	1.04859	8.73444	18.8454	1.39886	9.54281	-2.79629	0.03388	0.77053	*Clec18a*	8
ENSMUSG00000019732	307.688	140.308	138.637	74.4501	117.43	143.584	195.544	111.821	0.80456	0.03391	0.77053	*Calr3*	8
ENSMUSG00000036977	344.366	328.641	337.648	246.419	230.007	265.63	336.885	247.352	0.44524	0.03392	0.77053	*Anapc10*	8
ENSMUSG00000092581	34.6404	7.53331	11.1804	9.43734	0	0	17.7847	3.14578	2.52999	0.034	0.77053	*Gm3211*	9
ENSMUSG00000045662	12.226	0.94166	7.82627	4.19437	21.3509	56.5362	6.99798	27.3605	-1.97955	0.03402	0.77053	*Henmt1*	3
ENSMUSG00000032387	3073.82	2056.59	2874.48	1529.9	1879.85	2279.39	2668.3	1896.38	0.49236	0.03405	0.77053	*Rbpms2*	9

ENSMUSG00000026239	1178.79	958.614	1195.18	851.458	796.775	874.067	1110.86	840.767	0.40156	0.03405	0.77053	*Pde6d*	1
ENSMUSG00000019726	1769.71	782.522	1366.24	616.573	855.976	1037.39	1306.16	836.647	0.64211	0.03406	0.77053	*Lyst*	13
ENSMUSG00000059900	18.339	30.1332	20.1247	85.9846	74.728	11.6662	22.8656	57.4596	-1.32371	0.03407	0.77053	*Tmem40*	6
ENSMUSG00000104864	1.01883	0	0	3.14578	11.6459	1.7948	0.33961	5.52883	-3.91321	0.0341	1	*Gm43655*	3
ENSMUSG00000001630	918.988	730.731	1019.65	573.58	688.08	692.792	889.79	651.484	0.44894	0.03414	0.77112	*Stk38l*	6
ENSMUSG00000051606	20.3767	14.125	17.8886	7.34015	36.8788	105.893	17.4634	50.0374	-1.5239	0.03418	0.77112	*2010001K21Rik*	13
ENSMUSG00000020679	26.4897	7.53331	12.2984	13.6317	66.9641	42.1778	15.4405	40.9245	-1.41023	0.03423	0.77112	*Hnf1b*	11
ENSMUSG00000073411	2841.53	1688.4	2353.47	928.005	1554.73	1999.41	2294.47	1494.05	0.61857	0.03433	0.77112	*H2-D1*	17
ENSMUSG00000091900	7.13184	5.64998	21.2427	12.5831	51.4362	31.409	11.3415	31.8094	-1.5035	0.03434	0.77112	*Gm4353*	7
ENSMUSG00000087714	22.4143	1.88333	7.82627	0	0.97049	3.5896	10.708	1.52003	2.77873	0.03442	0.77112	*Gm25827*	4
ENSMUSG00000054169	0	0	0	6.29156	1.94099	3.5896	0	3.94071	-4.3845	0.03448	1	*Ceacam10*	7
ENSMUSG00000113198	3.0565	1.88333	3.35411	2.09719	10.6754	26.922	2.76465	13.2315	-2.27445	0.03452	0.77112	*Gm6988*	12
ENSMUSG00000113885	2.03767	0	0	1.04859	7.76395	14.3584	0.67922	7.72364	-3.50995	0.03452	0.77112	*Gm47802*	13
ENSMUSG00000071072	2995.37	2375.82	3744.31	2193.66	2068.12	2395.16	3038.5	2218.98	0.45323	0.03453	0.77112	*Ptges3*	10
ENSMUSG00000027824	106.978	57.4415	91.6791	25.1662	19.4099	76.279	85.3661	40.285	1.07656	0.03454	0.77112	*Vmn2r1*	3
ENSMUSG00000064936	11.2072	2.82499	12.2984	1.04859	0	2.6922	8.77686	1.24693	2.786	0.03455	0.77112	*Gm23722*	1
ENSMUSG00000021432	891.48	639.39	1035.3	523.248	575.503	703.561	855.391	600.771	0.50879	0.03456	0.77112	*Slc35b3*	13
ENSMUSG00000032915	25.4708	7.53331	15.6525	3.14578	4.85247	6.2818	16.2189	4.76001	1.7572	0.03458	0.77112	*Adgre4*	17

ENSMUSG00000081296	19.3578	8.47497	23.4788	1.04859	3.88198	8.97399	17.1039	4.63485	1.85852	0.03463	0.77147	*Gm12203*	11
ENSMUSG00000041245	729.485	317.341	816.168	278.926	275.62	540.234	620.998	364.927	0.76567	0.03471	0.77232	*Wnk3*	X
ENSMUSG00000107278	142.637	112.058	192.303	68.1586	90.2559	116.662	148.999	91.6921	0.6958	0.03488	0.77376	*Gm42600*	6
ENSMUSG00000040466	683.638	1291.02	816.168	4007.72	2298.13	872.272	930.275	2392.71	-1.36278	0.03489	0.77376	*Blvrb*	7
ENSMUSG00000022422	170.145	150.666	147.581	260.051	230.007	186.659	156.131	225.572	-0.52948	0.0349	0.77376	*Dscc1*	15
ENSMUSG00000071192	31.5839	29.1916	21.2427	6.29156	8.73444	17.948	27.3394	10.9913	1.30503	0.03492	0.77376	*Wfikkn1*	17
ENSMUSG00000106120	25.4708	4.70832	17.8886	2.09719	0.97049	8.07659	16.0226	3.71476	2.08632	0.035	0.77431	*Gm42697*	3
ENSMUSG00000023008	2083.52	1517.02	1713.95	1305.5	1283.96	1407.12	1771.5	1332.19	0.41101	0.03503	0.77431	*Fmnl3*	15
ENSMUSG00000096944	19.3578	26.3666	40.2494	13.6317	12.6164	10.7688	28.6579	12.339	1.21479	0.03506	0.77431	*Gm26722*	4
ENSMUSG00000008129	644.922	291.916	674.177	145.754	306.676	454.982	537.005	302.471	0.82655	0.03522	0.77529	*4930432K21Rik*	8
ENSMUSG00000026616	6.113	0.94166	4.47215	20.9719	14.5574	8.07659	3.84227	14.5353	-1.92877	0.03522	0.77529	*Cr2*	1
ENSMUSG00000026319	2299.51	1212.86	2771.62	1190.15	1304.34	1630.57	2094.66	1375.02	0.60687	0.03523	0.77529	*Relch*	1
ENSMUSG00000032913	1956.16	1257.12	2132.1	1075.86	1314.05	1376.61	1781.79	1255.51	0.50462	0.03525	0.77529	*Lrig2*	3
ENSMUSG00000037697	505.342	474.598	616.039	676.343	690.992	778.045	531.993	715.127	-0.42776	0.03535	0.7758	*Ddhd1*	14
ENSMUSG00000043800	2.03767	0	0	2.09719	8.73444	10.7688	0.67922	7.20014	-3.40748	0.03535	0.7758	*Kifc5c-ps*	10
ENSMUSG00000115057	5.09417	0	8.9443	0	0	0	4.67949	0	4.7063	0.03554	1	*Gm8705*	15
ENSMUSG00000041642	391.232	428.457	499.763	357.57	321.233	278.194	439.817	318.999	0.46367	0.03557	0.77656	*Kif21b*	1
ENSMUSG00000000125	3.0565	0	1.11804	2.09719	15.5279	10.7688	1.39151	9.46463	-2.7833	0.03561	0.77656	*Wnt3*	11

ENSMUSG00000032419	3.0565	1.88333	4.47215	4.19437	8.73444	27.8194	3.13733	13.5827	-2.13459	0.03561	0.77656	*Tbx18*	9
ENSMUSG00000063887	30.565	22.5999	15.6525	50.3325	38.8198	45.7674	22.9392	44.9732	-0.96688	0.03564	0.77656	*Nlgn1*	3
ENSMUSG00000103694	3.0565	0.94166	7.82627	4.19437	23.2919	20.6402	3.94148	16.0421	-2.05367	0.03565	0.77656	*Gm37530*	4
ENSMUSG00000041237	549.151	365.365	261.621	1708.16	1544.06	288.963	392.046	1180.39	-1.59	0.03565	0.77656	*Pklr*	3
ENSMUSG00000020024	2123.25	1068.79	2124.27	969.949	1097.63	1447.51	1772.1	1171.69	0.59641	0.03566	0.77656	*Cep83*	10
ENSMUSG00000108584	8.15067	4.70832	2.23608	0	0.97049	0	5.03169	0.3235	3.85864	0.0357	1	*Gm45216*	6
ENSMUSG00000031845	2.03767	0.94166	1.11804	4.19437	5.82296	15.2558	1.36579	8.42438	-2.6336	0.03572	0.77656	*Bco1*	8
ENSMUSG00000085389	2.03767	0.94166	1.11804	4.19437	8.73444	11.6662	1.36579	8.19834	-2.5927	0.03572	0.77656	*1700003M07Rik*	4
ENSMUSG00000025968	2485.95	1865.44	2550.24	1717.6	1644.02	1826.21	2300.55	1729.27	0.41157	0.03602	0.78219	*Ndufs1*	1
ENSMUSG00000085180	64.1865	32.9582	36.8953	58.7212	77.6395	105.893	44.68	80.7513	-0.85644	0.03608	0.78264	*AI838599*	4
ENSMUSG00000030180	5540.42	2520.83	5051.3	2322.63	2932.83	3384.99	4370.85	2880.15	0.6016	0.03612	0.78264	*Kdm5a*	6
ENSMUSG00000023345	190.522	202.458	145.345	247.468	254.269	262.938	179.442	254.892	-0.50479	0.03616	0.78273	*Poc1a*	9
ENSMUSG00000086279	0	0	0	3.14578	2.91148	5.3844	0	3.81389	-4.34269	0.03619	1	*Gm15634*	8
ENSMUSG00000095539	34.6404	10.3583	22.3608	37.7494	49.4952	53.844	22.4531	47.0295	-1.07263	0.03633	0.78374	*Gm25287*	10
ENSMUSG00000048188	8.15067	5.64998	6.70823	1.04859	0.97049	0.8974	6.83629	0.97216	2.81637	0.03634	0.78374	*Gm8181*	18
ENSMUSG00000082079	4.07534	1.88333	5.59019	10.4859	17.4689	12.5636	3.84962	13.5061	-1.82827	0.03634	0.78374	*Dnmt3c*	2
ENSMUSG00000082063	29.5462	8.47497	27.951	1.04859	9.70494	8.97399	21.9907	6.57584	1.72569	0.03636	0.78374	*Gm12993*	4
ENSMUSG00000085502	6.113	1.88333	3.35411	0	0	0	3.78348	0	4.4045	0.03639	1	*Gm12320*	11

ENSMUSG00000083255	6.113	1.88333	3.35411	0	0	0	3.78348	0	4.4045	0.03639	1	*Gm12738*	4
ENSMUSG00000058071	0	0	0	0	8.73444	5.3844	0	4.70628	-4.6469	0.03648	1	*Olfr818*	10
ENSMUSG00000029538	1415.16	1747.73	2033.71	1190.15	1236.41	1425.07	1732.2	1283.88	0.43178	0.03648	0.78569	*Srsf9*	5
ENSMUSG00000111212	22.4143	0.94166	14.5345	4.19437	0.97049	1.7948	12.6302	2.31989	2.45485	0.03667	0.78597	*Gm47087*	9
ENSMUSG00000025340	507.379	457.649	414.792	720.384	607.529	545.619	459.94	624.51	-0.44046	0.03667	0.78597	*Rabgef1*	5
ENSMUSG00000046196	17.3202	15.0666	17.8886	15.7289	31.0558	74.4842	16.7585	40.423	-1.2769	0.0367	0.78597	*Ttc39d*	17
ENSMUSG00000020490	21.3955	128.066	226.962	1265.65	610.441	116.662	125.475	664.251	-2.40455	0.03675	0.78597	*Btnl10*	11
ENSMUSG00000022025	10.1883	10.3583	5.59019	44.0409	18.4394	11.6662	8.71228	24.7155	-1.49168	0.03679	0.78597	*Cnmd*	14
ENSMUSG00000049001	212.936	110.175	182.24	294.655	237.771	237.811	168.45	256.746	-0.60909	0.03692	0.78597	*Ndnf*	6
ENSMUSG00000028063	1067.74	1332.45	915.673	2155.91	1835.2	1007.78	1105.29	1666.3	-0.59172	0.03693	0.78597	*Lmna*	3
ENSMUSG00000005683	5953.05	4075.52	5393.42	3632.33	4147.89	3738.57	5140.66	3839.59	0.42092	0.03693	0.78597	*Cs*	10
ENSMUSG00000043795	77.4314	73.4498	59.256	97.5192	135.869	98.7139	70.0457	110.701	-0.65864	0.03694	0.78597	*Prr33*	7
ENSMUSG00000020185	165.051	124.3	153.171	261.1	196.04	191.146	147.507	216.095	-0.55078	0.03695	0.78597	*E2f7*	10
ENSMUSG00000107741	245.539	142.191	281.746	103.811	135.869	176.788	223.159	138.823	0.68153	0.03696	0.78597	*Gm2011*	3
ENSMUSG00000020520	303.613	404.915	391.313	777.008	485.247	393.061	366.614	551.772	-0.58897	0.03699	0.78597	*Galnt10*	11
ENSMUSG00000085139	64.1865	51.7915	41.3674	30.4092	24.2623	31.409	52.4485	28.6935	0.87138	0.03699	0.78597	*A730046J19Rik*	X
ENSMUSG00000092574	7.13184	0.94166	16.7706	0	1.94099	0.8974	8.28136	0.94613	3.10371	0.03705	0.78639	*2810047C21Rik*	7
ENSMUSG00000075266	528.775	317.341	406.966	223.35	314.44	314.987	417.694	284.259	0.55406	0.03714	0.78704	*Cenpw*	10

ENSMUSG00000046591	1289.84	610.198	1261.15	665.857	661.877	773.558	1053.73	700.431	0.58864	0.03715	0.78704	*Ticrr*	7
ENSMUSG00000045349	26.4897	12.2416	22.3608	20.9719	41.7312	75.3816	20.364	46.0282	-1.18466	0.0372	0.78712	*Sh2d5*	4
ENSMUSG00000027397	1088.11	808.889	1284.63	1723.89	1468.36	1257.26	1060.54	1483.17	-0.48413	0.03725	0.78745	*Slc20a1*	2
ENSMUSG00000090935	992.344	742.973	1056.55	695.217	610.441	742.149	930.621	682.602	0.44659	0.03738	0.7885	*Synj2bp*	12
ENSMUSG00000003872	26.4897	30.1332	33.5411	12.5831	17.4689	11.6662	30.0547	13.9061	1.11167	0.03741	0.7885	*Lin7b*	7
ENSMUSG00000078607	46.8664	36.7249	40.2494	59.7698	65.0231	85.2529	41.2802	70.0153	-0.76497	0.03741	0.7885	*1810010H24Rik*	11
ENSMUSG00000079173	29.5462	58.3831	42.4854	68.1586	66.9641	94.2269	43.4716	76.4499	-0.81319	0.03746	0.78878	*Zan*	5
ENSMUSG00000031701	3737.08	2961.53	4206.06	2770.38	2630.04	2835.78	3634.89	2745.4	0.40474	0.03766	0.7919	*Dnaja2*	8
ENSMUSG00000021176	49.9229	71.5664	65.9642	108.005	82.492	109.483	62.4845	99.9933	-0.67781	0.03769	0.7919	*Efcab11*	12
ENSMUSG00000106107	0	0	1.11804	2.09719	7.76395	5.3844	0.37268	5.08184	-3.79564	0.03774	1	*Gm43190*	3
ENSMUSG00000021066	754.956	335.232	731.197	225.448	366.847	507.031	607.128	366.442	0.72706	0.03779	0.79198	*Atl1*	12
ENSMUSG00000031893	15.2825	10.3583	13.4165	12.5831	35.9083	46.6648	13.0191	31.7187	-1.29187	0.0378	0.79198	*Tsnaxip1*	8
ENSMUSG00000042351	19.3578	46.1415	97.2693	251.662	83.4625	48.4596	54.2562	127.861	-1.23795	0.03781	0.79198	*Grap2*	15
ENSMUSG00000102880	0	0	0	0	5.82296	8.07659	0	4.63319	-4.62603	0.03791	1	*4930517J16Rik*	4
ENSMUSG00000055717	52.9794	67.7998	74.9086	95.422	87.3444	131.918	65.2292	104.895	-0.68794	0.03792	0.79292	*Slain1*	14
ENSMUSG00000028158	401.421	219.408	471.812	152.046	217.391	303.321	364.213	224.253	0.69735	0.03793	0.79292	*Mttp*	3
ENSMUSG00000050890	963.817	623.381	1004	606.087	536.683	690.1	863.732	610.957	0.49882	0.03804	0.79426	*Pdik1l*	4
ENSMUSG00000060461	79.469	58.3831	200.129	23.069	24.2623	98.7139	112.66	48.6818	1.20386	0.03812	0.79426	*Dppa5a*	9

ENSMUSG00000047067	249.614	307.924	241.496	203.427	146.545	205.504	266.345	185.159	0.52535	0.0382	0.79426	*Dusp28*	1
ENSMUSG00000051147	56.0359	65.9165	88.325	222.302	92.1969	74.4842	70.0924	129.661	-0.88643	0.03823	0.79426	*Nat2*	8
ENSMUSG00000050439	6.113	8.47497	17.8886	7.34015	38.8198	46.6648	10.8255	30.9416	-1.52779	0.03824	0.79426	*Enthd1*	15
ENSMUSG00000027652	3978.55	1947.36	3190.88	1741.71	2064.24	2405.93	3038.93	2070.63	0.55328	0.03827	0.79426	*Ralgapb*	2
ENSMUSG00000024892	3503.77	1792.93	2767.14	1462.79	1923.52	2125.04	2687.95	1837.12	0.54882	0.03829	0.79426	*Pcx*	19
ENSMUSG00000035202	8580.62	3306.18	2909.14	3139.49	2985.24	2925.52	4931.98	3016.75	0.7092	0.0383	0.79426	*Lars2*	9
ENSMUSG00000099632	56.0359	43.3165	44.7215	29.3606	28.1443	20.6402	48.0246	26.0484	0.88584	0.03846	0.79673	*2900093K20Rik*	2
ENSMUSG00000030546	44.8287	69.6831	67.0823	37.7494	34.9378	30.5116	60.5314	34.3996	0.81727	0.0385	0.79682	*Plin1*	7
ENSMUSG00000002831	28.5274	30.1332	30.187	12.5831	19.4099	7.1792	29.6159	13.0574	1.18591	0.03863	0.79845	*Plin4*	17
ENSMUSG00000071230	6.113	8.47497	7.82627	0	0	2.6922	7.47141	0.8974	2.99974	0.03876	0.79845	*Npw*	17
ENSMUSG00000037130	1.01883	0	0	2.09719	5.82296	7.1792	0.33961	5.03311	-3.78343	0.03878	1	*H2-M10.6*	17
ENSMUSG00000048416	538.963	242.949	777.036	257.954	256.21	407.419	519.65	307.195	0.75693	0.03882	0.79845	*Mlf1*	3
ENSMUSG00000039158	170.145	211.874	160.997	262.148	293.089	220.76	181.006	258.666	-0.51314	0.03887	0.79845	*Akna*	4
ENSMUSG00000058402	320.933	256.133	221.372	196.087	210.597	129.226	266.146	178.637	0.57695	0.0389	0.79845	*Zfp420*	7
ENSMUSG00000091649	13.2448	5.64998	8.9443	1.04859	1.94099	2.6922	9.27971	1.89393	2.27536	0.03891	0.79845	*Phf11b*	14
ENSMUSG00000023328	63.1677	153.491	174.414	909.13	488.158	85.2529	130.358	494.181	-1.92248	0.03891	0.79845	*Ache*	5
ENSMUSG00000023170	855.821	903.997	778.155	1455.45	1122.86	916.245	845.991	1164.85	-0.46082	0.03897	0.79845	*Gps2*	11
ENSMUSG00000031343	79.469	65.9165	60.3741	111.151	98.0199	108.585	68.5865	105.919	-0.62595	0.03901	0.79845	*Gabra3*	X

ENSMUSG00000032999	15.2825	11.3	33.5411	4.19437	2.91148	11.6662	20.0412	6.25735	1.66038	0.03909	0.79845	*Nlrp4f*	13
ENSMUSG00000021065	2700.93	2034.94	2962.8	1850.77	1776	2103.5	2566.22	1910.09	0.42573	0.03912	0.79845	*Fut8*	12
ENSMUSG00000003813	519.605	703.423	673.059	1633.71	878.297	552.798	632.029	1021.6	-0.69228	0.03913	0.79845	*Rad23a*	8
ENSMUSG00000036561	643.903	700.598	727.843	999.309	973.405	776.251	690.781	916.322	-0.40739	0.03916	0.79845	*Ppp6r2*	15
ENSMUSG00000028273	1181.85	601.723	1330.47	594.552	705.549	764.584	1038.01	688.229	0.59217	0.03916	0.79845	*Pdlim5*	3
ENSMUSG00000031220	9.16951	1.88333	5.59019	15.7289	16.4984	18.8454	5.54767	17.0242	-1.62774	0.03917	0.79845	*Awat2*	X
ENSMUSG00000026180	1.01883	5.64998	2.23608	27.2634	5.82296	7.1792	2.9683	13.4219	-2.154	0.03919	0.79845	*Cxcr2*	1
ENSMUSG00000096029	0	0	0	3.14578	1.94099	6.2818	0	3.78952	-4.33433	0.03921	1	*Olfr679*	7
ENSMUSG00000060791	16.3013	14.125	14.5345	47.1867	31.0558	20.6402	14.9869	32.9609	-1.13276	0.03929	0.79845	*Gmfg*	7
ENSMUSG00000086725	281.198	116.766	174.414	100.665	132.958	116.662	190.793	116.762	0.70728	0.03929	0.79845	*A630052C17Rik*	11
ENSMUSG00000054074	369.837	300.391	337.648	242.225	241.653	256.656	335.958	246.845	0.44405	0.03931	0.79845	*Skida1*	2
ENSMUSG00000022789	4150.73	2761.9	4491.16	2635.11	2627.13	3027.83	3801.26	2763.36	0.45987	0.03942	0.79878	*Dnm1l*	16
ENSMUSG00000086507	9.16951	9.41664	5.59019	0	2.91148	0.8974	8.05878	1.26963	2.65603	0.03942	0.79878	*Adap2os*	11
ENSMUSG00000091745	14.2637	21.6583	19.0066	1.04859	10.6754	5.3844	18.3095	5.70281	1.67443	0.03956	0.79878	*Gm17098*	9
ENSMUSG00000097486	37.6969	15.0666	49.1937	17.8261	13.5869	12.5636	33.9857	14.6589	1.21113	0.03959	0.79878	*Gm17733*	6
ENSMUSG00000021276	1380.52	926.597	1343.88	729.821	823.949	1049.96	1217	867.909	0.48705	0.03959	0.79878	*Cinp*	12
ENSMUSG00000066113	150.787	159.141	155.407	241.176	223.214	191.146	155.112	218.512	-0.49333	0.0396	0.79878	*Adamtsl1*	4
ENSMUSG00000076862	0	0	17.8886	0	0	0	5.96287	0	5.05581	0.03961	1	*Trav19*	14

ENSMUSG00000058355	3414.11	2276	3455.86	2271.25	2163.23	2315.29	3048.66	2249.92	0.43813	0.03965	0.79878	*Abce1*	8
ENSMUSG00000027857	170.145	46.1415	72.6725	15.7289	26.2033	81.6633	96.3198	41.1985	1.21954	0.03974	0.79878	*Tshb*	3
ENSMUSG00000030075	88.6386	110.175	152.053	241.176	165.954	144.481	116.955	183.871	-0.65314	0.03974	0.79878	*Cntn3*	6
ENSMUSG00000041805	852.764	276.849	902.257	114.297	243.594	400.24	677.29	252.71	1.42165	0.03977	0.79878	*Pramel1*	4
ENSMUSG00000054893	801.822	499.082	847.473	549.463	439.634	516.005	716.126	501.7	0.51287	0.03981	0.79878	*Zfp667*	7
ENSMUSG00000082396	171.164	69.6831	150.935	19.9233	57.2591	110.38	130.594	62.5208	1.05736	0.03983	0.79878	*Zfp389*	13
ENSMUSG00000094096	0	7.53331	0	2.09719	17.4689	27.8194	2.5111	15.7952	-2.62168	0.03983	0.79878	*Gm25649*	13
ENSMUSG00000022847	19.3578	16.9499	31.3051	20.9719	49.4952	76.279	22.5376	48.9153	-1.12741	0.03993	0.79878	*Thpo*	16
ENSMUSG00000023341	51.9605	21.6583	41.3674	16.7775	8.73444	25.1272	38.3287	16.8797	1.17662	0.03999	0.79878	*Mx2*	16
ENSMUSG00000046215	3.0565	5.64998	12.2984	1.04859	0.97049	0.8974	7.00163	0.97216	2.84519	0.04001	0.79878	*Rprml*	11
ENSMUSG00000060143	89.6574	270.257	105.096	187.698	331.909	303.321	155.003	274.309	-0.82163	0.04002	0.79878	*Gm10076*	14
ENSMUSG00000021361	1214.45	1827.77	1563.02	3947.95	2507.76	1145.08	1535.08	2533.6	-0.72265	0.04002	0.79878	*Tmem14c*	13
ENSMUSG00000109420	8.15067	12.2416	4.47215	1.04859	2.91148	0	8.28815	1.32002	2.67032	0.04008	0.79927	*Gm44702*	7
ENSMUSG00000087052	74.3749	29.1916	83.8529	13.6317	27.1738	45.7674	62.4731	28.8576	1.10499	0.04013	0.79946	*Gm8093*	9
ENSMUSG00000094958	19.3578	14.125	12.2984	0	4.85247	7.1792	15.2604	4.01055	1.90631	0.04028	0.80011	*3110021N24Rik*	4
ENSMUSG00000095698	0	4.70832	1.11804	10.4859	7.76395	12.5636	1.94212	10.2712	-2.37226	0.0403	0.80011	*Rhox2d*	X
ENSMUSG00000028351	92.7139	72.5081	57.0199	152.046	90.2559	117.559	74.0806	119.954	-0.6926	0.04035	0.80011	*Brinp1*	4
ENSMUSG00000039396	269.991	244.833	566.845	1031.82	517.273	349.986	360.556	633.025	-0.81244	0.04036	0.80011	*Neil3*	8

ENSMUSG00000096146	19.3578	21.6583	52.5478	13.6317	11.6459	14.3584	31.188	13.212	1.23274	0.04037	0.80011	*Kcnj11*	7
ENSMUSG00000025537	5.09417	17.8916	8.9443	25.1662	32.0263	22.435	10.6434	26.5425	-1.30824	0.0404	0.80011	*Phkg1*	5
ENSMUSG00000085609	11.2072	1.88333	2.23608	0	0.97049	0	5.10886	0.3235	3.87786	0.04043	1	*1700016P03Rik*	11
ENSMUSG00000036053	3762.55	3390.93	5131.79	3012.61	2939.63	3190.25	4095.09	3047.5	0.4261	0.04043	0.80011	*Fmnl2*	2
ENSMUSG00000034412	571.566	732.614	604.859	429.923	518.244	474.724	636.346	474.297	0.42419	0.0405	0.80011	*Tbc1d10a*	11
ENSMUSG00000020023	89.6574	60.2665	88.325	141.56	98.0199	133.713	79.4163	124.431	-0.64997	0.04054	0.80011	*Tmcc3*	10
ENSMUSG00000041064	164.032	200.574	159.879	389.028	235.83	187.556	174.829	270.805	-0.62902	0.04055	0.80011	*Pif1*	9
ENSMUSG00000081960	0	0	0	0	12.6164	1.7948	0	4.80374	-4.6742	0.04057	1	*Mageb17-ps*	X
ENSMUSG00000097236	163.013	65.9165	69.3184	29.3606	58.2296	68.2024	99.4161	51.9309	0.93361	0.04061	0.80046	*4831440E17Rik*	5
ENSMUSG00000097391	283.236	301.332	223.608	377.494	343.555	389.471	269.392	370.173	-0.45731	0.04074	0.80241	*Mirg*	12
ENSMUSG00000034031	36.678	92.283	51.4298	131.074	80.551	106.791	60.1303	106.139	-0.8153	0.04096	0.806	*Ccdc182*	11
ENSMUSG00000096982	66.2242	125.241	145.345	655.371	358.112	79.8685	112.27	364.451	-1.6986	0.04109	0.80769	*Redrum*	18
ENSMUSG00000029650	111.053	148.783	98.3874	282.072	214.479	104.996	119.408	200.515	-0.74461	0.04116	0.80789	*Slc46a3*	5
ENSMUSG00000033382	3685.12	1535.85	3156.22	1411.41	1743.01	2270.42	2792.4	1808.28	0.62663	0.04119	0.80789	*Trappc8*	18
ENSMUSG00000097718	1.01883	0.94166	0	5.24297	2.91148	10.7688	0.6535	6.30775	-3.23843	0.04119	1	*Gm26896*	10
ENSMUSG00000038344	3392.72	1264.65	3299.33	1054.88	1520.76	2237.22	2652.23	1604.29	0.72496	0.04122	0.80789	*Txlng*	X
ENSMUSG00000051910	590.924	363.482	919.027	1538.29	920.028	647.025	624.478	1035.11	-0.72939	0.04132	0.80827	*Sox6*	7
ENSMUSG00000016526	49.9229	90.3997	136.401	277.877	141.692	95.1243	92.2411	171.565	-0.8954	0.04133	0.80827	*Dyrk3*	1

ENSMUSG00000043282	6.113	6.59165	6.70823	19.9233	17.4689	15.2558	6.47096	17.5493	-1.43793	0.04135	0.80827	*Teddm1b*	1
ENSMUSG00000064330	2.03767	5.64998	6.70823	0	0	0.8974	4.79863	0.29913	3.78556	0.04141	1	*Pde6h*	6
ENSMUSG00000056708	462.551	575.356	393.549	671.1	691.962	590.489	477.152	651.184	-0.44746	0.04165	0.81257	*Ier5*	1
ENSMUSG00000063193	8.15067	8.47497	3.35411	22.0205	16.4984	17.0506	6.65992	18.5231	-1.46234	0.04165	0.81257	*Cd300lb*	11
ENSMUSG00000094724	99.8457	145.958	168.824	104.859	86.3739	74.4842	138.209	88.5725	0.64297	0.04188	0.81585	*Rnaset2b*	17
ENSMUSG00000068259	49.9229	8.47497	13.4165	0	9.70494	8.97399	23.9381	6.22631	1.93077	0.0419	0.81585	*Olfr461*	6
ENSMUSG00000020808	327.046	465.182	344.356	268.44	276.591	276.399	378.861	273.81	0.46929	0.04195	0.81616	*Pimreg*	11
ENSMUSG00000097726	8.15067	9.41664	6.70823	3.14578	0.97049	0	8.09185	1.37209	2.6048	0.04199	0.8162	*9530036O11Rik*	5
ENSMUSG00000109058	17.3202	0	0	0	0	0	5.77339	0	5.01623	0.04217	1	*Olfr705*	7
ENSMUSG00000059657	36.678	40.4915	34.6592	156.24	57.2591	27.8194	37.2762	80.4396	-1.10532	0.04225	0.82005	*Stfa2l1*	16
ENSMUSG00000019986	408.552	407.74	429.327	596.65	492.04	554.593	415.206	547.761	-0.39972	0.04227	0.82005	*Ahi1*	10
ENSMUSG00000115338	398.364	350.299	338.766	633.35	555.122	364.344	362.476	517.606	-0.51306	0.04234	0.8206	*Pnp*	14
ENSMUSG00000053012	1281.69	987.805	1369.6	896.547	779.307	1018.55	1213.03	898.134	0.43312	0.04239	0.82077	*Krcc1*	6
ENSMUSG00000035451	6.113	0.94166	1.11804	3.14578	13.5869	21.5376	2.72424	12.7568	-2.23221	0.04246	0.82103	*Foxa1*	12
ENSMUSG00000053508	28.5274	14.125	10.0623	8.38875	4.85247	4.487	17.5715	5.9094	1.58238	0.04248	0.82103	*Gtsf2*	15
ENSMUSG00000085560	53.9982	6.59165	42.4854	9.43734	6.79346	19.7428	34.3584	11.9912	1.50941	0.04258	0.82179	*C230038L03Rik*	7
ENSMUSG00000020228	283.236	291.916	328.703	588.261	424.106	307.808	301.285	440.058	-0.54589	0.0426	0.82179	*Helb*	10
ENSMUSG00000073000	1.01883	9.41664	6.70823	18.8747	24.2623	11.6662	5.71457	18.2677	-1.66953	0.04288	0.82483	*Gm10451*	12

ENSMUSG00000061533	237.388	205.283	287.336	478.158	356.171	252.169	243.336	362.166	-0.57362	0.04293	0.82483	*Cep128*	12
ENSMUSG00000082033	91.6951	32.9582	105.096	14.6803	66.9641	17.948	76.583	33.1975	1.20337	0.04301	0.82483	*Mageb10-ps*	X
ENSMUSG00000042401	32.6027	10.3583	20.1247	19.9233	55.3181	67.305	21.0286	47.5155	-1.18248	0.04303	0.82483	*Crtac1*	19
ENSMUSG00000048732	445.23	127.125	250.441	98.5678	188.276	176.788	274.265	154.544	0.82608	0.04307	0.82483	*Klhl11*	11
ENSMUSG00000025492	499.229	537.69	512.061	357.57	254.269	470.237	516.327	360.692	0.51695	0.04307	0.82483	*Ifitm3*	7
ENSMUSG00000026725	12.226	0.94166	8.9443	39.8465	16.4984	13.461	7.37066	23.2686	-1.66544	0.04314	0.82483	*Tnn*	1
ENSMUSG00000011832	1296.98	1172.37	1156.05	908.082	870.533	1028.42	1208.47	935.678	0.36892	0.04317	0.82483	*Evi5l*	8
ENSMUSG00000108521	3.0565	0.94166	1.11804	6.29156	8.73444	10.7688	1.7054	8.59827	-2.33776	0.04322	0.82483	*Gm44639*	7
ENSMUSG00000085572	6.113	3.76665	12.2984	7.34015	14.5574	48.4596	7.39269	23.4524	-1.68389	0.04323	0.82483	*Gm16234*	10
ENSMUSG00000062391	77.4314	80.9831	45.8396	110.102	101.902	113.072	68.0847	108.359	-0.66649	0.04325	0.82483	*4932435O22Rik*	11
ENSMUSG00000078566	476.814	390.79	465.104	340.793	330.938	328.448	444.236	333.393	0.41373	0.04326	0.82483	*Bnip3*	7
ENSMUSG00000073716	197.654	82.8664	95.0332	46.1381	106.754	50.2544	125.184	67.7156	0.88614	0.04327	0.82483	*Gm13241*	4
ENSMUSG00000024786	928.158	366.307	836.293	221.253	393.05	627.282	710.252	413.862	0.77803	0.04333	0.82507	*Majin*	19
ENSMUSG00000106103	21.3955	29.1916	22.3608	14.6803	10.6754	4.487	24.3159	9.94758	1.30274	0.04338	0.82517	*Gm43081*	5
ENSMUSG00000069306	25.4708	8.47497	2.23608	5.24297	0.97049	0.8974	12.0606	2.37029	2.37473	0.04345	0.82517	*Hist1h4m*	13
ENSMUSG00000075707	55.017	74.3914	40.2494	89.1304	81.5215	108.585	56.5526	93.0791	-0.71504	0.04356	0.82517	*Dio3*	12
ENSMUSG00000029178	900.649	892.697	1332.7	2392.89	1340.25	1052.65	1042.02	1595.26	-0.6144	0.04356	0.82517	*Klf3*	5
ENSMUSG00000057054	948.534	520.74	633.928	255.857	432.84	634.461	701.067	441.053	0.66762	0.04362	0.82517	*Inca1*	11

ENSMUSG00000095687	47.8852	54.6165	64.8462	33.555	26.2033	35.896	55.7826	31.8848	0.80489	0.04363	0.82517	*Rnaset2a*	17
ENSMUSG00000034997	16.3013	15.0666	7.82627	45.0895	21.3509	24.2298	13.0647	30.2234	-1.19965	0.04363	0.82517	*Htr2a*	14
ENSMUSG00000045179	14.2637	8.47497	11.1804	6.29156	32.0263	55.6388	11.3063	31.3189	-1.47691	0.04365	0.82517	*Sox3*	X
ENSMUSG00000078427	916.951	744.856	967.103	793.785	617.234	417.291	876.303	609.437	0.52446	0.04384	0.82586	*Sarnp*	10
ENSMUSG00000115725	6.113	1.88333	11.1804	39.8465	11.6459	10.7688	6.39224	20.7538	-1.71063	0.04385	0.82586	*4930572G02Rik*	14
ENSMUSG00000047773	5.09417	1.88333	1.11804	4.19437	24.2623	8.07659	2.69851	12.1778	-2.16972	0.04388	0.82586	*Ankfn1*	11
ENSMUSG00000026277	6224.06	3847.64	5039	2906.7	3750.96	4219.57	5036.9	3625.74	0.47412	0.04393	0.82586	*Stk25*	1
ENSMUSG00000037887	82.5255	114.883	125.22	299.898	154.309	101.406	107.543	185.204	-0.78266	0.04398	0.82586	*Dusp8*	7
ENSMUSG00000049287	239.426	308.866	226.962	560.997	398.873	239.606	258.418	399.825	-0.6279	0.04399	0.82586	*Iba57*	11
ENSMUSG00000103633	10.1883	3.76665	6.70823	0	0.97049	1.7948	6.88774	0.92176	2.86037	0.04401	0.82586	*Gm38328*	1
ENSMUSG00000024087	1940.88	1551.86	1709.48	1093.68	1391.69	1445.71	1734.07	1310.36	0.40388	0.04404	0.82586	*Cyp1b1*	17
ENSMUSG00000021678	12.226	21.6583	13.4165	58.7212	32.0263	17.948	15.7669	36.2318	-1.19055	0.04406	0.82586	*F2rl1*	13
ENSMUSG00000097429	0	0	0	5.24297	3.88198	1.7948	0	3.63991	-4.26886	0.04411	1	*Gm26520*	12
ENSMUSG00000027306	838.5	920.947	1397.55	2710.61	1313.08	1030.21	1052.33	1684.64	-0.67882	0.04411	0.82586	*Nusap1*	2
ENSMUSG00000040657	31.5839	24.4833	15.6525	100.665	65.9936	10.7688	23.9065	59.1424	-1.30126	0.04412	0.82586	*1700063H04Rik*	6
ENSMUSG00000043050	1.01883	0	1.11804	2.09719	8.73444	8.07659	0.71229	6.30274	-3.18852	0.0444	1	*Tnp2*	16
ENSMUSG00000087436	0	0.94166	0	6.29156	1.94099	6.2818	0.31389	4.83811	-3.72153	0.04443	1	*Gm16156*	8
ENSMUSG00000030549	46.8664	19.7749	44.7215	9.43734	19.4099	21.5376	37.1209	16.7949	1.13443	0.04447	0.83164	*Rhcg*	7

ENSMUSG00000034883	403.458	169.499	458.396	149.949	211.568	262.938	343.784	208.152	0.72176	0.04459	0.83187	*Lrr1*	12
ENSMUSG00000074704	1552.7	389.849	1332.7	210.767	326.086	663.178	1091.75	400.01	1.44817	0.0446	0.83187	*Rad21l*	2
ENSMUSG00000098202	10.1883	7.53331	6.70823	2.09719	1.94099	0.8974	8.14329	1.64519	2.32192	0.04461	0.83187	*B830012L14Rik*	12
ENSMUSG00000037469	17.3202	6.59165	13.4165	0	1.94099	6.2818	12.4428	2.74093	2.1494	0.04466	0.83213	*Acp7*	7
ENSMUSG00000018143	188.484	267.432	329.821	725.626	365.876	231.529	261.913	441.011	-0.75123	0.04474	0.83284	*Mafk*	5
ENSMUSG00000035232	1899.11	1305.15	2034.83	1198.54	1252.91	1403.53	1746.36	1284.99	0.44218	0.04483	0.83372	*Pdk3*	X
ENSMUSG00000105284	4.07534	0	8.9443	0	0	0	4.33988	0	4.59697	0.04499	1	*Gm42693*	3
ENSMUSG00000025766	646.96	397.382	603.741	396.368	355.201	422.675	549.361	391.415	0.48827	0.04502	0.83654	*D3Ertd751e*	3
ENSMUSG00000112203	29.5462	25.4249	44.7215	14.6803	15.5279	17.948	33.2309	16.0521	1.04338	0.04507	0.83674	*Gm49167*	10
ENSMUSG00000101641	0	4.70832	0	9.43734	22.3214	1.7948	1.56944	11.1845	-2.78644	0.04517	0.83786	*Gm29560*	3
ENSMUSG00000022679	194.597	143.133	182.24	134.22	101.902	120.252	173.323	118.791	0.54473	0.04532	0.83991	*Mpv17l*	16
ENSMUSG00000025872	1109.51	1032.06	1362.89	865.089	857.917	948.551	1168.15	890.519	0.39099	0.04548	0.84114	*Thoc3*	13
ENSMUSG00000037738	0	5.64998	1.11804	4.19437	13.5869	17.0506	2.25601	11.6106	-2.33804	0.04551	0.84114	*Nek5*	8
ENSMUSG00000086363	13.2448	8.47497	22.3608	3.14578	2.91148	7.1792	14.6935	4.41215	1.71731	0.04554	0.84114	*A330102I10Rik*	13
ENSMUSG00000001632	1500.74	1035.83	1360.65	898.644	1029.69	991.626	1299.07	973.322	0.41614	0.04555	0.84114	*Brpf1*	6
ENSMUSG00000074215	139.58	109.233	84.9709	54.5268	67.9346	86.1503	111.261	69.5373	0.67648	0.04563	0.84197	*Gm10643*	8
ENSMUSG00000110126	1.01883	5.64998	52.5478	4.19437	5.82296	2.6922	19.7389	4.23651	2.21642	0.04571	0.84263	*Gm9347*	7
ENSMUSG00000061353	719.297	798.531	688.711	1248.87	776.395	993.421	735.513	1006.23	-0.45165	0.04587	0.84414	*Cxcl12*	6

ENSMUSG00000034041	173.202	198.691	229.198	659.565	296.001	134.61	200.364	363.392	-0.85807	0.04589	0.84414	*Lyl1*	8
ENSMUSG00000028008	0	0	0	0	5.82296	7.1792	0	4.33405	-4.52958	0.0459	1	*Asic5*	3
ENSMUSG00000086604	65.2054	28.2499	58.138	22.0205	26.2033	30.5116	50.5311	26.2451	0.93936	0.04592	0.84414	*Gm15510*	7
ENSMUSG00000051246	140.599	62.1498	82.7348	45.0895	56.2886	64.6128	95.1612	55.3303	0.77959	0.04598	0.84454	*Msantd1*	5
ENSMUSG00000030521	954.647	597.015	993.936	534.783	531.831	722.407	848.533	596.34	0.50794	0.04605	0.84462	*Mphosph10*	7
ENSMUSG00000020419	995.401	339.941	1304.75	195.038	295.03	548.311	880.031	346.127	1.34577	0.04606	0.84462	*Hormad2*	11
ENSMUSG00000074280	1.01883	7.53331	45.8396	6.29156	2.91148	2.6922	18.1306	3.96508	2.19497	0.04611	0.84472	*Gm6166*	9
ENSMUSG00000025747	588.886	662.931	592.56	1028.67	846.271	647.025	614.792	840.655	-0.45067	0.04616	0.84493	*Tyms*	5
ENSMUSG00000052539	1345.88	682.706	1264.5	612.378	710.401	909.963	1097.7	744.248	0.55994	0.04621	0.84493	*Magi3*	3
ENSMUSG00000081670	89.6574	30.1332	68.2003	14.6803	24.2623	49.357	62.6636	29.4332	1.0818	0.04624	0.84493	*Gm15697*	6
ENSMUSG00000106678	38.7157	46.1415	44.7215	18.8747	13.5869	33.2038	43.1929	21.8885	0.97489	0.04628	0.84493	*Gm43457*	5
ENSMUSG00000081188	0	0	0	7.34015	1.94099	1.7948	0	3.69198	-4.28727	0.04632	1	*Gm14621*	X
ENSMUSG00000105517	4.07534	1.88333	4.47215	0	0	0	3.47694	0	4.28055	0.04633	1	*Gm42436*	3
ENSMUSG00000070002	1067.74	922.83	1036.42	787.493	709.431	841.761	1009	779.562	0.37188	0.04643	0.8453	*Ell*	8
ENSMUSG00000026110	184.409	108.291	146.463	162.532	246.505	254.861	146.388	221.3	-0.59829	0.04648	0.8453	*Mgat4a*	1
ENSMUSG00000090071	1.01883	3.76665	2.23608	3.14578	12.6164	16.1532	2.34052	10.6385	-2.17999	0.0465	0.8453	*Cdk5r2*	1
ENSMUSG00000021929	2202.72	1478.41	2471.98	1486.91	1521.73	1502.25	2051.04	1503.63	0.44763	0.04653	0.8453	*Kpna3*	14
ENSMUSG00000059336	184.409	113	206.837	790.639	596.854	107.688	168.082	498.394	-1.56821	0.04654	0.8453	*Slc14a1*	18

ENSMUSG00000085282	19.3578	27.3082	30.187	44.0409	42.7017	52.9466	25.6177	46.5631	-0.86477	0.04657	0.8453	*Gm15663*	10
ENSMUSG00000043719	690.769	755.214	601.505	918.568	962.73	804.967	682.496	895.422	-0.39106	0.04659	0.8453	*Col6a6*	9
ENSMUSG00000109105	15.2825	4.70832	0	18.8747	252.328	5.3844	6.66361	92.1958	-3.78827	0.04669	0.84639	*Gm44873*	7
ENSMUSG00000078752	3.0565	6.59165	1.11804	0	0	0	3.58873	0	4.33667	0.0467	1	*Scgb1b30*	7
ENSMUSG00000104965	50.9417	39.5499	45.8396	24.1176	28.1443	23.3324	45.4437	25.1981	0.85013	0.04678	0.84692	*Gm43437*	3
ENSMUSG00000086464	22.4143	4.70832	15.6525	1.04859	3.88198	6.2818	14.2584	3.73745	1.91039	0.0468	0.84692	*Gm15835*	16
ENSMUSG00000026650	0	0	0	1.04859	0.97049	9.87139	0	3.96349	-4.40327	0.04682	1	*Meig1*	2
ENSMUSG00000087601	0	0	0	1.04859	0.97049	9.87139	0	3.96349	-4.40327	0.04682	1	*Uchl1os*	5
ENSMUSG00000052270	5.09417	0.94166	3.35411	26.2148	11.6459	2.6922	3.12998	13.5177	-2.11402	0.04699	0.84876	*Fpr2*	17
ENSMUSG00000022797	6505.25	4002.07	8406.53	16640.1	9579.74	5095.43	6304.62	10438.4	-0.72743	0.047	0.84876	*Tfrc*	16
ENSMUSG00000103445	30.565	32.9582	23.4788	48.2353	60.1706	45.7674	29.0007	51.3911	-0.82148	0.04705	0.84876	*Gm36948*	9
ENSMUSG00000084998	46.8664	43.3165	40.2494	25.1662	29.1148	15.2558	43.4774	23.1789	0.91213	0.04709	0.84876	*Gm16279*	17
ENSMUSG00000092274	369.837	413.39	297.398	574.629	724.959	327.551	360.208	542.38	-0.5894	0.04714	0.84876	*Neat1*	19
ENSMUSG00000107412	10.1883	4.70832	7.82627	0	1.94099	1.7948	7.57431	1.24526	2.57566	0.04721	0.84876	*Gm20383*	6
ENSMUSG00000021470	2445.2	1126.23	2175.7	1114.65	1192.74	1568.65	1915.71	1292.02	0.5679	0.04722	0.84876	*Ercc6l2*	13
ENSMUSG00000029372	38.7157	2.82499	13.4165	4.19437	1.94099	8.07659	18.319	4.73732	1.94143	0.0473	0.84876	*Ppbp*	5
ENSMUSG00000107849	5.09417	7.53331	14.5345	40.8951	39.7902	3.5896	9.05399	28.0917	-1.63701	0.04733	0.84876	*Gm43904*	6
ENSMUSG00000041607	246.558	202.458	237.024	395.32	380.434	224.35	228.68	333.368	-0.54322	0.04733	0.84876	*Mbp*	18

ENSMUSG00000085818	5.09417	0.94166	0	6.29156	15.5279	8.97399	2.01194	10.2645	-2.34182	0.04735	0.84876	*Gm13267*	2
ENSMUSG00000020267	2435.01	2306.13	2432.85	1838.18	1825.5	2035.3	2391.33	1899.66	0.33193	0.04752	0.84937	*Hint1*	11
ENSMUSG00000113020	2.03767	0	11.1804	0	0	0	4.40602	0	4.61746	0.04753	1	*Gm48063*	12
ENSMUSG00000039632	26.4897	17.8916	16.7706	22.0205	52.4067	50.2544	20.384	41.5605	-1.03021	0.04754	0.84937	*Ccdc151*	9
ENSMUSG00000059412	17.3202	52.7332	27.951	38.7979	107.725	54.7414	32.6681	67.088	-1.03446	0.04756	0.84937	*Fxyd2*	9
ENSMUSG00000061755	2848.66	1582.94	2752.61	1641.05	1647.9	1842.36	2394.74	1710.44	0.48525	0.04764	0.84937	*Bod1l*	5
ENSMUSG00000024663	700.958	1045.25	988.346	2228.26	1265.52	797.788	911.517	1430.52	-0.64985	0.04766	0.84937	*Rab3il1*	19
ENSMUSG00000038506	814.048	631.856	837.411	556.803	540.565	622.795	761.105	573.388	0.40787	0.04766	0.84937	*Dcun1d2*	8
ENSMUSG00000095040	8.15067	3.76665	6.70823	2.09719	0	0	6.20852	0.69906	3.2137	0.04766	1	*1700001J03 Rik*	5
ENSMUSG00000032254	2219.02	1237.35	2383.66	1623.22	1117.04	1289.56	1946.67	1343.27	0.53515	0.04768	0.84937	*Kif23*	9
ENSMUSG00000030922	306.669	125.241	232.552	73.4015	107.725	199.223	221.487	126.783	0.80178	0.04779	0.84937	*Lyrm1*	7
ENSMUSG00000093682	9.16951	10.3583	10.0623	17.8261	21.3509	29.6142	9.86338	22.9304	-1.22011	0.04782	0.84937	*Gm20681*	8
ENSMUSG00000097392	853.783	644.098	1151.58	560.997	604.618	713.433	883.153	626.349	0.49477	0.04783	0.84937	*D930016D0 6Rik*	5
ENSMUSG00000104093	15.2825	4.70832	6.70823	3.14578	0	1.7948	8.89969	1.64686	2.44689	0.04784	0.84937	*A330015K0 6Rik*	3
ENSMUSG00000030759	2769.19	2033.05	3194.23	2082.51	2009.89	1876.46	2665.49	1989.62	0.42176	0.04794	0.85007	*Far1*	7
ENSMUSG00000037685	1043.29	1206.27	1196.3	2327.88	1761.45	992.524	1148.62	1693.95	-0.5602	0.04803	0.85007	*Atp8a1*	5
ENSMUSG00000041827	45.8475	34.8416	26.8329	14.6803	9.70494	26.0246	35.8407	16.8033	1.08815	0.04805	0.85007	*Oasl1*	5
ENSMUSG00000093688	22.4143	6.59165	10.0623	2.09719	1.94099	6.2818	13.0228	3.43999	1.90309	0.04809	0.85007	*Gm8488*	5

ENSMUSG00000041920	141.618	106.408	137.519	315.627	257.181	90.6373	128.515	221.148	-0.78224	0.04811	0.85007	*Slc16a6*	11
ENSMUSG00000031465	129.392	86.6331	55.9019	115.345	162.072	162.429	90.6423	146.616	-0.69272	0.04817	0.85007	*Angpt2*	8
ENSMUSG00000068205	485.984	308.866	410.32	168.824	301.824	330.243	401.723	266.963	0.5879	0.04824	0.85007	*Macrod2*	2
ENSMUSG00000073139	1100.34	925.655	1211.95	761.279	834.625	879.451	1079.32	825.118	0.38684	0.04825	0.85007	*Tmem185a*	X
ENSMUSG00000061601	317.876	154.433	272.801	100.665	131.987	222.555	248.37	151.736	0.70806	0.04825	0.85007	*Pclo*	5
ENSMUSG00000086206	23.4332	2.82499	12.2984	2.09719	2.91148	4.487	12.8522	3.16522	2.0083	0.04849	0.85309	*Gm13783*	2
ENSMUSG00000113637	24.452	18.8333	2.23608	7.34015	2.91148	0.8974	15.1738	3.71634	2.05403	0.04859	0.85309	*Gm7049*	13
ENSMUSG00000097467	82.5255	102.641	84.9709	42.9923	47.5542	75.3816	90.0459	55.3094	0.70008	0.04866	0.85309	*Gm26737*	9
ENSMUSG00000022111	676.506	595.131	838.529	400.563	455.162	644.333	703.389	500.019	0.49108	0.04868	0.85309	*Uchl3*	14
ENSMUSG00000020264	5.09417	2.82499	5.59019	11.5345	10.6754	19.7428	4.50312	13.9842	-1.64814	0.0487	0.85309	*Slc36a2*	11
ENSMUSG00000028961	2714.17	2459.63	2477.57	1881.18	2106.94	2079.27	2550.46	2022.46	0.33456	0.04871	0.85309	*Pgd*	4
ENSMUSG00000099759	117.166	123.358	124.102	111.151	52.4067	64.6128	121.542	76.0568	0.67964	0.04876	0.85309	*1700030C10Rik*	12
ENSMUSG00000026878	4402.38	3684.73	5533.17	3677.42	3232.71	3384.99	4540.09	3431.71	0.4037	0.04882	0.85309	*Rab14*	2
ENSMUSG00000026502	1228.71	969.914	1473.57	935.345	999.609	771.764	1224.07	902.239	0.4399	0.04883	0.85309	*Desi2*	1
ENSMUSG00000065401	6.113	7.53331	13.4165	77.5959	21.3509	0.8974	9.02092	33.2814	-1.88388	0.04886	0.85309	*Mir144*	11
ENSMUSG00000089827	18.339	6.59165	15.6525	19.9233	22.3214	53.844	13.5277	32.0295	-1.25422	0.04887	0.85309	*1700023H06Rik*	13
ENSMUSG00000068686	12.226	32.9582	19.0066	70.2557	48.5247	21.5376	21.397	46.7727	-1.11972	0.04892	0.85317	*Cd59b*	2
ENSMUSG00000048170	1887.9	1525.5	2213.72	1468.03	1283.96	1498.66	1875.7	1416.88	0.40442	0.04904	0.85345	*Mcmbp*	7

ENSMUSG00000085827	28.5274	7.53331	14.5345	3.14578	7.76395	5.3844	16.8651	5.43138	1.62768	0.04905	0.85345	*Gm11290*	13
ENSMUSG00000022489	293.424	139.366	195.657	121.637	113.548	166.916	209.482	134.034	0.64248	0.04906	0.85345	*Pde1b*	15
ENSMUSG00000042345	40.7534	42.3749	33.5411	83.8875	95.1084	34.9986	38.8898	71.3315	-0.87088	0.04931	0.85711	*Ubash3a*	17
ENSMUSG00000059654	4.07534	0.94166	5.59019	0	0	0	3.53573	0	4.30231	0.04936	1	*Reg1*	6
ENSMUSG00000106913	3.0565	0.94166	6.70823	0	0	0	3.5688	0	4.31429	0.04941	1	*9430007M09Rik*	5
ENSMUSG00000030172	1086.08	952.022	1090.09	737.161	885.09	801.378	1042.73	807.876	0.36781	0.04941	0.85821	*Erc1*	6
ENSMUSG00000050240	353.535	312.632	420.382	175.115	279.502	298.834	362.183	251.15	0.52611	0.04953	0.85947	*Hic2*	16
ENSMUSG00000026475	137.543	152.55	148.699	105.908	94.1379	106.791	146.264	102.279	0.51613	0.04965	0.86093	*Rgs16*	1
ENSMUSG00000022867	835.444	775.931	1170.59	2269.16	1259.7	822.018	927.32	1450.29	-0.64514	0.04974	0.8618	*Usp25*	16
ENSMUSG00000017716	1107.47	919.064	1406.49	2134.94	1519.79	1219.57	1144.34	1624.76	-0.50578	0.04979	0.8619	*Birc5*	11
ENSMUSG00000113495	51.9605	51.7915	45.8396	33.555	20.3804	30.5116	49.8639	28.149	0.82685	0.04983	0.8619	*Gm19792*	13
ENSMUSG00000091478	338.253	351.241	383.487	340.793	612.382	573.438	357.66	508.871	-0.50963	0.04998	0.86381	*Gm10039*	11
ENSMUSG00000062410	1.01883	0	1.11804	5.24297	6.79346	5.3844	0.71229	5.80694	-3.06663	0.04999	1	*Hsd3b3*	3
