# Supplementary material for: SRSF1 regulates primordial follicle formation and number determination during meiotic prophase I
Source: BMC Biol. 2023 Mar 8;21:49. doi: 10.1186/s12915-023-01549-7 (PMC9993595; doi:10.1186/s12915-023-01549-7)
Supplement: Supplementary file 3 — Additional file 3: Table 2. AS events were analysed in cKO and Ctrl ovaries. [file 12915_2023_1549_MOESM3_ESM.pdf]

Additional file 3: Table 2. Alternative splicing events were analyzed in cKO and Ctrl ovaries.

GeneID	geneSymbol	chr	type	IncFormLen	SkipFormLen	PValue	FDR	IncLevel1	IncLevel2	IncLevelDifference
ENSMUSG00000049580	*Tsku*	7	SE	289	149	0.00012249	0.026162	1.0,0.607,1.0	0.673,0.0,0.34	0.531
ENSMUSG00000048647	*Exd1*	2	SE	2.43E+02	149	0.00000962	0.003352	0.0,1.0,0.0	1.0,0.88,1.0	-0.627
ENSMUSG00000029470	*P2rx4*	5	SE	230	149	0.00020746	0.03837	0.841,0.802,0.83	1.0,1.0,0.913	-0.147
ENSMUSG00000022685	*Parn*	16	SE	215	149	0.00029179	0.048332	0.8,0.812,0.914	1.0,1.0,0.945	-0.14
ENSMUSG00000036151	*Tm6sf2*	8	SE	253	149	0.000033	0.009081	0.469,0.884,0.485	1.0,1.0,1.0	-0.387
ENSMUSG00000073968	*Trim68*	7	SE	172	149	0.0000317	0.008818	1.0,0.95,1.0	0.257,0.65,0.786	0.419
ENSMUSG00000041536	*Serpina3a*	12	SE	8.09E+02	149	0.0000136	0.004193	0.968,0.145,1.0	0.123,0.075,0.0	0.638
ENSMUSG00000037605	*Adgrl3*	5	SE	353	149	0.00010204	0.023832	1.0,1.0,1.0	1.0,0.709,0.601	0.23
ENSMUSG00000063889	*Crem*	18	SE	338	149	0.0000218	0.006215	0.665,0.428,0.829	0.841,1.0,1.0	-0.306
ENSMUSG00000018347	*Zkscan6*	11	SE	249	149	0.000042	0.011153	0.0,1.0,0.0	1.0,1.0,1.0	-0.667
ENSMUSG00000022305	*Lrp12*	15	SE	206	149	0.00000993	0.003382	0.925,1.0,1.0	0.52,0.897,0.542	0.322
ENSMUSG00000035126	*Wdr78*	4	SE	3.01E+02	149	0.00000364	0.001575	0.856,1.0,1.0	0.11,0.476,0.731	0.513
ENSMUSG00000025812	*Pard3*	8	SE	194	149	0.00015574	0.030824	0.339,0.277,0.0	0.434,0.434,1.0	-0.417
ENSMUSG00000029128	*Rab28*	5	SE	244	149	4.53E-08	7.05E-05	0.244,0.152,0.258	0.042,0.028,0.091	0.164
ENSMUSG00000011831	*Evi5*	5	SE	296	149	0.0000212	0.006123	0.735,0.935,0.491	0.934,1.0,1.0	-0.258
ENSMUSG00000057766	*Ankrd29*	18	SE	248	149	4.71E-09	8.46E-06	0.231,0.546,0.419	0.9,1.0,1.0	-0.568
ENSMUSG00000034035	*Ccdc17*	4	SE	2.60E+02	149	0.00021239	0.038452	0.696,1.0,1.0	0.247,0.741,0.632	0.359
ENSMUSG00000046311	*Zfp62*	11	SE	278	149	0.00020471	0.03837	0.255,0.172,0.251	0.085,0.051,0.065	0.159

ENSMUSG00000040003	*Magi2*	5	SE	191	149	8.26E-07	0.000553	0.438,0.163,0.0	0.701,1.0,1.0	-0.7
ENSMUSG00000034354	*Mtmr3*	11	SE	260	149	4.13E-10	9.64E-07	0.4,0.31,0.305	0.632,0.601,0.762	-0.327
ENSMUSG00000090841	*Myl6*	10	SE	256	149	0.00025274	0.043725	0.259,0.162,0.0	0.671,1.0,0.466	-0.572
ENSMUSG00000028256	*Odf2l*	3	SE	3.08E+02	149	0.00016681	0.032739	0.064,0.2,0.163	0.053,0.008,0.02	0.115
ENSMUSG00000071562	*Stfa1*	16	SE	344	149	6.14E-07	0.000463	1.0,1.0,1.0	0.657,0.818,0.722	0.268
ENSMUSG00000024381	*Bin1*	18	SE	278	149	0.000029	0.00815	0.258,0.258,0.211	0.624,0.428,0.527	-0.284
ENSMUSG00000024381	*Bin1*	18	SE	278	149	0.0000381	0.010215	0.088,0.118,0.203	0.315,0.331,0.325	-0.187
ENSMUSG00000035439	*Haus8*	8	SE	215	149	1.06E-07	0.000142	0.47,0.72,0.687	0.964,0.883,0.91	-0.293
ENSMUSG00000029775	*Klhdc10*	6	SE	2.36E+02	149	0.00000642	0.002458	0.219,0.075,0.117	1.0,0.267,0.919	-0.592
ENSMUSG00000028759	*Hp1bp3*	4	SE	347	149	5.12E-09	8.55E-06	0.859,0.995,0.969	0.771,0.741,0.732	0.193
ENSMUSG00000026991	*Pkp4*	2	SE	278	149	0.00000234	0.001164	0.292,0.373,0.356	0.566,0.665,0.767	-0.326
ENSMUSG00000000673	*Haao*	17	SE	295	149	0.00000486	0.001993	0.288,0.562,0.144	0.716,1.0,1.0	-0.574
ENSMUSG00000035325	*Sec31a*	5	SE	188	149	0.0000832	0.020649	0.959,0.978,1.0	0.877,0.798,0.786	0.159
ENSMUSG00000055436	*Srsf11*	3	SE	2.58E+02	149	1.55E-07	0.00019	1.0,1.0,0.852	0.719,0.366,0.188	0.526
ENSMUSG00000039662	*Icmt*	4	SE	258	149	0.00014066	0.028566	0.064,0.0,0.016	0.208,0.161,0.224	-0.171
ENSMUSG00000025237	*Parp6*	9	SE	219	149	0.000084	0.020649	0.026,0.0,0.02	0.226,0.19,0.099	-0.156
ENSMUSG00000030451	*Herc2*	7	SE	257	149	0.00007	0.017961	0.722,0.68,0.67	1.0,0.813,0.923	-0.221
ENSMUSG00000029456	*Acad10*	5	SE	298	149	4.51E-07	0.000352	0.933,1.0,1.0	0.778,0.625,0.533	0.332
ENSMUSG00000032178	*Ilf3*	9	SE	1.88E+02	149	9.72E-10	2.06E-06	1.0,1.0,1.0	0.671,0.704,0.74	0.295
ENSMUSG00000061306	*Slc38a10*	11	SE	267	149	6.34E-08	9.25E-05	0.751,0.817,0.668	0.99,0.98,0.887	-0.207

ENSMUSG00000066842	*Hmcn1*	1	SE	500	149	0.0000879	0.021373	0.145,0.266,0.1	0.296,0.361,0.346	-0.164
ENSMUSG00000028328	*Tmod1*	4	SE	306	149	0.00012174	0.026162	1.0,1.0,0.898	0.892,0.705,0.678	0.208
ENSMUSG00000023044	*Csad*	15	SE	239	149	0.0000791	0.020072	1.0,1.0,1.0	1.0,0.757,0.63	0.204
ENSMUSG00000023044	*Csad*	15	SE	2.01E+02	149	0.00014332	0.028855	1.0,1.0,1.0	1.0,0.617,0.58	0.268
ENSMUSG00000070371	*Prss36*	7	SE	291	149	0.00000418	0.001776	0.719,0.869,0.738	0.339,0.369,0.146	0.491
ENSMUSG00000064061	*Dzip3*	16	SE	767	149	0.000012	0.003853	0.383,0.504,0.457	0.604,0.718,0.757	-0.245
ENSMUSG00000048731	*Ggnbp1*	17	SE	239	149	0.00010583	0.024232	1.0,0.93,1.0	0.681,0.799,0.862	0.196
ENSMUSG00000062115	*Rai1*	11	SE	279	149	0.0000135	0.004193	1.0,1.0,0.762	0.428,0.722,0.673	0.313
ENSMUSG00000021000	*Mia2*	12	SE	1.76E+02	149	0.00000336	0.001508	0.025,0.0,0.0	0.127,0.229,0.188	-0.173
ENSMUSG00000004267	*Eno2*	6	SE	283	149	0.00010396	0.024039	0.441,0.612,0.26	1.0,1.0,0.798	-0.495
ENSMUSG00000052407	*Ccdc171*	4	SE	288	149	0.00010937	0.024799	1.0,1.0,1.0	0.608,0.564,0.627	0.4
ENSMUSG00000043987	*Cep164*	9	SE	296	149	0.00000671	0.002501	0.834,1.0,1.0	0.573,0.701,0.724	0.279
ENSMUSG00000042772	*Smg7*	1	SE	299	149	0.00021718	0.038872	0.088,0.065,0.163	0.185,0.327,0.444	-0.213
ENSMUSG00000028649	*Macf1*	4	SE	2.51E+02	149	7.05E-07	0.000499	0.275,0.085,0.283	0.05,0.017,0.049	0.176
ENSMUSG00000036860	*Mrpl55*	11	SE	230	149	0.00015238	0.030417	0.769,0.826,0.764	0.942,1.0,1.0	-0.194
ENSMUSG00000025728	*Pigq*	17	SE	261	149	7.02E-07	0.000499	0.718,0.851,0.605	0.975,0.946,0.984	-0.244
ENSMUSG00000025728	*Pigq*	17	SE	361	149	0.00000139	0.000758	0.715,0.83,0.575	0.967,0.933,0.981	-0.254
ENSMUSG00000025728	*Pigq*	17	SE	303	149	3.33E-07	0.000332	0.349,0.39,0.135	0.879,0.653,0.887	-0.515
ENSMUSG00000025728	*Pigq*	17	SE	7.07E+02	149	0.00000117	0.000665	0.741,0.689,0.654	0.945,0.883,0.974	-0.239
ENSMUSG00000032182	*Yipf2*	9	SE	210	149	0.00000089	0.000577	0.771,0.73,0.707	0.902,1.0,1.0	-0.231

ENSMUSG00000031392	*Irak1*	X	SE	266	149	4.04E-10	9.64E-07	1.0,1.0,1.0	0.528,0.528,0.296	0.549
ENSMUSG00000027263	*Tubgcp4*	2	SE	274	149	0.00011796	0.026162	0.768,0.605,0.826	1.0,1.0,0.839	-0.213
ENSMUSG00000036196	*Slc26a8*	17	SE	345	149	0.00021804	0.038872	1.0,1.0,1.0	1.0,0.665,0.647	0.229
ENSMUSG00000005225	*Plekha8*	6	SE	3.05E+02	149	1.81E-07	0.000211	0.781,0.796,0.729	1.0,1.0,1.0	-0.231
ENSMUSG00000019841	*Rev3l*	10	SE	276	149	0.0000369	0.010012	0.33,0.21,0.365	0.018,0.143,0.197	0.182
ENSMUSG00000020513	*Tubd1*	11	SE	242	149	0.00025077	0.043725	0.025,0.0,0.055	0.381,0.159,0.133	-0.198
ENSMUSG00000056458	*Mok*	12	SE	323	149	0.00000182	0.000946	0.958,0.806,1.0	0.495,0.217,0.609	0.481
ENSMUSG00000006673	*Qrich1*	9	SE	209	149	0.0001981	0.037688	1.0,1.0,0.829	0.72,0.588,0.695	0.275
ENSMUSG00000021832	*Psmc6*	14	SE	3.05E+02	149	6.66E-16	7.78E-12	0.328,0.0,0.163	1.0,1.0,1.0	-0.836
ENSMUSG00000039361	*Picalm*	7	SE	299	149	0.0000922	0.021965	0.164,0.253,0.313	0.542,0.419,0.384	-0.205
ENSMUSG00000027680	*Fxr1*	3	SE	230	149	0.0000921	0.021965	0.179,0.116,0.165	0.011,0.035,0.081	0.111
ENSMUSG00000003435	*Supt5*	7	SE	161	149	0.00000115	0.000665	0.0,0.0,0.108	0.467,0.195,0.229	-0.261
ENSMUSG00000053390	*Zfp952*	17	SE	259	149	0.00020865	0.03837	0.418,0.379,0.535	0.076,0.161,0.079	0.339
ENSMUSG00000006527	*Sfmbt1*	14	SE	2.41E+02	149	0.000015	0.004493	0.883,0.812,1.0	0.464,0.284,0.684	0.421
ENSMUSG00000027605	*Acss2*	2	SE	188	149	4.53E-07	0.000352	0.32,0.186,0.268	0.105,0.097,0.029	0.181
ENSMUSG00000097080	*1700086O06Rik*	18	SE	328	149	1.99E-14	1.32E-10	0.45,0.132,0.312	1.0,1.0,1.0	-0.702
ENSMUSG00000097080	*1700086O06Rik*	18	SE	264	149	0	0	0.0,0.22,0.0	1.0,1.0,1.0	-0.927
ENSMUSG00000028689	*Ccdc163*	4	SE	183	149	0.0000064	0.002458	1.0,1.0,1.0	0.808,0.765,0.62	0.269
ENSMUSG00000051285	*Pcmtd1*	1	SE	3.41E+02	149	0.0000117	0.003781	0.29,0.274,0.106	0.052,0.0,0.051	0.189
ENSMUSG00000022791	*Tnk2*	16	SE	245	149	0.00000104	0.000622	1.0,1.0,0.966	0.924,0.856,0.76	0.142

ENSMUSG00000031948	*Kars*	8	SE	207	149	0.00025101	0.043725	0.0,0.057,0.0	0.216,0.141,0.11	-0.137
ENSMUSG00000033486	*Catsper2*	2	SE	249	149	0.00013721	0.028111	1.0,0.807,0.952	0.705,0.677,0.732	0.215
ENSMUSG00000074247	*Dda1*	8	SE	201	149	0.00012434	0.026162	0.969,0.966,1.0	0.862,0.828,0.869	0.125
ENSMUSG00000023286	*Ube2j2*	4	SE	3.12E+02	149	0.00000813	0.00292	0.284,0.362,0.398	1.0,0.642,0.607	-0.402
ENSMUSG00000016200	*Syt14*	1	SE	1541	149	8.29E-07	0.000553	0.583,0.441,0.768	0.088,0.244,0.132	0.443
ENSMUSG00000010592	*Dazl*	17	SE	200	149	2.26E-14	1.32E-10	0.784,0.845,0.814	0.965,0.974,0.973	-0.156
ENSMUSG00000085601	*Gm4969*	7	SE	256	149	0.0000108	0.003543	0.957,0.954,0.936	0.862,0.73,0.835	0.14
ENSMUSG00000109032	*Gm7972*	9	SE	325	149	0.00011942	0.026162	0.88,0.93,0.818	1.0,0.981,1.0	-0.118
ENSMUSG00000109032	*Gm7972*	9	SE	2.34E+02	149	0.00000355	0.001563	0.641,0.858,0.609	1.0,0.905,1.0	-0.266
ENSMUSG00000109032	*Gm7972*	9	SE	234	149	0.00000036	0.000336	0.734,0.723,0.736	0.448,0.427,0.411	0.302
ENSMUSG00000033287	*Kctd17*	15	SE	237	149	0.00000011	0.000142	0.112,0.218,0.284	0.0,0.0,0.0	0.205
ENSMUSG00000017718	*Afmid*	11	SE	240	149	0.00012266	0.026162	1.0,1.0,1.0	0.713,0.7,0.0	0.529
ENSMUSG00000020461	*Clhc1*	11	SE	256	149	9.72E-07	0.000598	0.461,1.0,0.951	0.0,0.0,0.368	0.681
ENSMUSG00000040097	*Flywch1*	17	SE	2.47E+02	149	0.00019849	0.037688	0.424,0.63,1.0	0.194,0.105,0.245	0.503
ENSMUSG00000027198	*Ext2*	2	SE	221	149	0.00011354	0.025498	0.447,0.574,0.752	1.0,1.0,0.752	-0.326
ENSMUSG00000064326	*Siva1*	12	SE	344	149	0.00000999	0.003382	0.829,0.868,0.791	0.925,0.938,0.957	-0.111
ENSMUSG00000000915	*Hip1r*	5	SE	248	149	0.00017206	0.033486	0.445,0.678,0.546	1.0,0.546,1.0	-0.292
ENSMUSG00000074219	*Gm10644*	8	SE	257	149	0.0000561	0.014546	0.777,1.0,1.0	0.367,0.465,0.367	0.526
ENSMUSG00000023927	*Satb1*	17	SE	2.45E+02	149	0.00000515	0.002037	0.0,0.0,0.08	1.0,1.0,0.255	-0.725
ENSMUSG00000031808	*Slc27a1*	8	SE	240	149	0.00000335	0.001508	0.0,0.0,0.16	0.498,0.461,0.262	-0.354

ENSMUSG00000037818	*Abhd18*	3	SE	201	149	0.0000133	0.004193	0.503,0.076,0.4	0.0,0.0,0.096	0.294
ENSMUSG00000022789	*Dnm1l*	16	SE	227	149	4.42E-07	0.000352	0.198,0.332,0.247	0.644,0.524,0.557	-0.316
ENSMUSG00000014592	*Camta1*	4	SE	170	149	0.0000148	0.004493	0.063,0.0,0.0	0.763,0.539,0.127	-0.455
ENSMUSG00000027806	*Tsc22d2*	3	SE	3.08E+02	149	0.00026261	0.044769	0.0,1.0,0.0	0.53,1.0,1.0	-0.51
ENSMUSG00000031004	*Mki67*	7	SE	275	149	9.1E-11	3.04E-07	0.921,1.0,1.0	0.659,0.52,0.575	0.389
ENSMUSG00000031004	*Mki67*	7	SE	1109	149	3.52E-09	6.85E-06	0.962,1.0,1.0	0.72,0.749,0.747	0.249
ENSMUSG00000039531	*Zup1*	10	SE	284	149	4.03E-07	0.000349	0.636,0.63,0.638	0.197,0.344,0.112	0.417
ENSMUSG00000051592	*Ccnb3*	X	SE	305	149	0.00000291	0.001361	1.0,1.0,0.898	0.746,0.612,0.71	0.277
ENSMUSG00000051592	*Ccnb3*	X	SE	1.90E+02	149	0.0002069	0.03837	0.758,1.0,1.0	1.0,0.381,0.641	0.245
ENSMUSG00000030041	*M1ap*	6	SE	290	149	0.00000209	0.001063	0.731,0.73,0.795	0.85,0.862,0.968	-0.141
ENSMUSG00000025372	*Baiap2*	11	SE	197	149	0.00000122	0.000678	0.327,0.204,0.385	0.0,0.076,0.064	0.259
ENSMUSG00000055447	*Cd47*	16	SE	212	149	0.00000504	0.002028	0.115,0.142,0.123	0.274,0.35,0.5	-0.248
ENSMUSG00000007411	*Mark3*	12	SE	194	149	0.0002235	0.039545	0.86,0.75,0.876	0.469,0.647,0.628	0.247
ENSMUSG00000032582	*Rbm6*	9	SE	2.56E+02	149	0.00000675	0.002501	0.44,0.196,0.636	0.0,0.0,0.115	0.386
ENSMUSG00000059939	*9430015G10Rik*	4	SE	245	149	0.00000168	0.000891	0.633,0.796,0.753	1.0,1.0,0.935	-0.251
ENSMUSG00000094152	*Slc6a16*	7	SE	391	149	2.74E-07	0.000291	0.807,1.0,1.0	0.0,0.276,0.433	0.699
ENSMUSG00000030323	*Ift122*	6	SE	326	149	0.00000242	0.001178	0.014,0.037,0.0	0.291,0.109,0.171	-0.173
ENSMUSG00000038145	*Snrk*	9	SE	199	149	0.0002774	0.046609	0.428,0.2,0.13	1.0,0.428,1.0	-0.557
ENSMUSG00000026987	*Baz2b*	2	SE	3.38E+02	149	0.0000104	0.003471	0.907,1.0,1.0	0.869,0.569,0.332	0.379
ENSMUSG00000009628	*Tex15*	8	SE	254	149	9.7E-12	3.78E-08	0.0,0.0,0.0	1.0,1.0,0.524	-0.841

ENSMUSG00000072964	*Bhlhb9*	X	SE	211	149	0.0000045	0.001877	1.0,1.0,0.809	0.0,0.261,0.653	0.632
ENSMUSG00000027722	*Spata5*	3	SE	186	149	6.62E-12	3.09E-08	0.24,0.348,0.385	0.14,0.079,0.091	0.221
ENSMUSG00000046774	*8030474K03Rik*	X	SE	204	149	0.0000443	0.011623	0.982,1.0,0.967	0.836,0.745,0.827	0.18
ENSMUSG00000023088	*Abcc1*	16	SE	3.42E+02	149	0.000016	0.004724	0.232,0.195,0.127	0.545,0.813,0.623	-0.476
ENSMUSG00000042515	*Mum1l1*	X	SE	217	149	0.0002104	0.03839	1.0,0.878,1.0	0.507,0.706,0.768	0.299
ENSMUSG00000027523	*Gnas*	2	SE	244	149	0.00012817	0.026654	0.0,1.0,1.0	0.0,1.0,0.0	0.333
ENSMUSG00000032621	*Srek1*	13	SE	273	149	0.00027468	0.046487	0.645,0.401,0.621	0.136,0.274,0.329	0.309
ENSMUSG00000034940	*Synrg*	11	SE	452	149	0.00012415	0.026162	1.0,1.0,1.0	0.676,0.761,0.737	0.275
ENSMUSG00000021276	*Cinp*	12	SE	2.99E+02	149	0.00012896	0.026654	0.0,0.199,1.0	1.0,0.499,1.0	-0.433
ENSMUSG00000014444	*Piezo1*	8	SE	211	149	0.0000204	0.005955	0.927,0.886,0.85	0.493,0.55,0.526	0.365
ENSMUSG00000020439	*Smtn*	11	SE	200	149	0.0000816	0.020483	0.0,0.0,0.271	0.246,0.211,0.19	-0.125
ENSMUSG00000039585	*Myo9a*	9	SE	206	149	9.63E-07	0.000598	0.375,0.367,0.116	0.566,1.0,0.943	-0.55
ENSMUSG00000023882	*Zfp54*	17	SE	189	149	1.62E-10	4.74E-07	0.0,0.0,0.0	1.0,0.825,1.0	-0.942
ENSMUSG00000019791	*Hint3*	10	SE	2.44E+02	149	0.00028716	0.047905	0.147,0.083,0.219	0.0,0.013,0.03	0.135
ENSMUSG00000026074	*Map4k4*	1	SE	311	149	0.00000706	0.002577	0.166,0.125,0.156	0.313,0.494,0.428	-0.263
ENSMUSG00000053333	*Dis3l2*	1	SE	227	149	0.00000903	0.003195	0.522,0.451,1.0	0.0,0.0,0.0	0.658
ENSMUSG00000036333	*Kidins220*	12	SE	263	149	0.00018414	0.035542	0.831,0.633,0.737	0.503,0.489,0.492	0.239
ENSMUSG00000028073	*Pear1*	3	SE	283	149	0.00025681	0.044101	1.0,1.0,1.0	0.648,0.612,0.612	0.376
ENSMUSG00000048440	*Cyp4f16*	17	SE	2.94E+02	149	3.41E-07	0.000332	0.797,0.695,0.736	1.0,1.0,1.0	-0.257
ENSMUSG00000048440	*Cyp4f16*	17	SE	203	149	3.88E-07	0.000348	0.72,0.68,0.786	1.0,1.0,1.0	-0.271

ENSMUSG00000042787	*Exog*	9	SE	263	149	2.57E-07	0.000286	0.288,0.274,0.298	0.739,1.0,0.819	-0.566
ENSMUSG00000029635	*Cdk8*	5	SE	278	149	0.0000934	0.022036	0.882,0.855,0.771	0.673,0.514,0.619	0.234
ENSMUSG00000009995	*Taz*	X	SE	240	149	0.00000261	0.001243	0.027,0.0,0.055	0.156,0.145,0.284	-0.168
ENSMUSG00000085396	*Firre*	X	A3SS	208	149	3.07E-12	5.53E-09	0.968,1.0,1.0	0.448,0.662,0.518	0.447
ENSMUSG00000019178	*Styxl1*	5	A3SS	152	149	1.81E-06	0.001305	0.898,1.0,1.0	0.797,0.729,0.512	0.287
ENSMUSG00000026090	*2010300C02Rik*	1	A3SS	152	149	2.20E-05	0.008812	0.0,0.0,0.329	0.164,1.0,1.0	-0.612
ENSMUSG00000039637	*Coro7*	16	A3SS	171	149	6.39E-07	0.000577	0.0,0.635,0.0	1.0,0.685,1.0	-0.683
ENSMUSG00000030774	*Pak1*	7	A3SS	152	149	0.00011969	0.04322	0.495,1.0,1.0	0.376,0.404,0.949	0.255
ENSMUSG00000010453	*Kansl3*	1	A3SS	161	149	0	0	1.0,1.0,1.0	0.0,0.236,0.316	0.816
ENSMUSG00000020131	*Pcsk4*	10	A3SS	179	149	1.56E-05	0.00705	0.555,0.543,0.806	1.0,1.0,0.934	-0.343
ENSMUSG00000084128	*Esrp2*	8	A3SS	152	149	3.97E-08	4.78E-05	1.0,1.0,1.0	0.246,0.574,0.0	0.727
ENSMUSG00000112023	*Lilr4b*	10	A3SS	646	149	3.49E-06	0.001799	0.187,0.0,0.316	0.698,1.0,1.0	-0.732
ENSMUSG00000110393	*Gm36445*	13	A3SS	348	149	2.41E-06	0.001453	0.0,0.0,0.125	1.0,0.485,0.692	-0.684
ENSMUSG00000041459	*Tardbp*	4	A5SS	158	149	6.46E-05	1.64E-02	0.663,0.705,0.361	0.9,0.845,1.0	-0.339
ENSMUSG00000023393	*Slc17a9*	2	A5SS	219	149	1.00E-08	1.45E-05	1.0,0.803,1.0	0.576,0.0,0.0	0.742
ENSMUSG00000026107	*Nabp1*	1	A5SS	728	149	1.98E-04	0.040884	0.954,0.799,0.938	0.808,0.545,0.633	0.235
ENSMUSG00000026107	*Nabp1*	1	A5SS	625	149	7.13E-05	0.016375	0.966,0.849,0.952	0.84,0.578,0.682	0.222
ENSMUSG00000038412	*Higd1a*	9	A5SS	161	149	1.9692E-05	0.007822	0.066,0.481,0.799	1.0,1.0,1.0	-0.551
ENSMUSG00000007035	*Msh5*	17	A5SS	184	149	2.2718E-05	0.007822	0.703,0.589,0.741	0.939,0.89,0.932	-0.243
ENSMUSG00000074247	*Dda1*	8	A5SS	278	149	1.01E-05	0.0052	0.941,0.944,1.0	0.802,0.748,0.801	0.178

ENSMUSG00000085024	*C230035I16Rik*	13	A5SS	242	149	3.26E-07	2.24E-04	0.804,0.939,0.812	0.396,0.235,0.335	0.53
ENSMUSG00000024293	*Esco1*	18	A5SS	2079	149	3.93E-05	0.011603	0.722,0.875,0.89	0.93,1.0,1.0	-0.148
ENSMUSG00000015957	*Wnt11*	7	A5SS	313	149	1.40E-08	1.45E-05	1.0,1.0,1.0	0.749,0.67,0.826	0.252
ENSMUSG00000040434	*Large2*	2	MEX	254	262	5.28E-08	8.41E-05	0.0,0.0,0.079	0.549,0.256,0.307	-0.344
ENSMUSG00000038371	*Sbf2*	7	MEX	238	287	5.94E-05	0.01253	0.317,0.598,0.633	0.153,0.097,0.156	0.381
ENSMUSG00000038766	*Gabpb2*	3	MEX	204	246	0.00017734	0.027428	0.099,0.376,0.0	0.668,0.437,0.311	-0.314
ENSMUSG00000024381	*Bin1*	18	MEX	278	221	5.65E-05	0.01253	0.09,0.143,0.169	0.43,0.266,0.34	-0.211
ENSMUSG00000001419	*Mef2d*	3	MEX	287	284	0.00011477	0.019019	0.915,0.733,0.84	0.965,1.0,0.967	-0.148
ENSMUSG00000025968	*Ndufs1*	1	MEX	257	226	5.04E-05	0.01253	0.582,0.549,0.687	0.417,0.418,0.371	0.204
ENSMUSG00000021098	*4930447C04Rik*	12	MEX	288	207	0.00011392	0.019019	0.399,0.439,0.369	0.556,0.53,0.532	-0.137
ENSMUSG00000024726	*Carnmt1*	19	MEX	328	263	6.86E-05	0.013256	0.394,0.902,0.8	0.15,0.271,0.231	0.481
ENSMUSG00000020672	*Sntg2*	12	MEX	277	279	1.43E-05	0.004405	0.0,0.168,0.0	0.668,0.751,0.335	-0.529
ENSMUSG00000025964	*Adam23*	1	MEX	240	240	1.52E-05	0.004405	0.2,0.0,0.0	0.714,0.375,0.5	-0.463
ENSMUSG00000022789	*Dnm1l*	16	MEX	227	182	1.01E-05	0.003894	0.327,0.431,0.354	0.742,0.696,0.545	-0.29
ENSMUSG00000094724	*Rnaset2b*	17	MEX	202	207	9.07E-07	0.000526	0.924,0.612,1.0	0.328,0.307,0.543	0.453
ENSMUSG00000030495	*Slc7a10*	7	MEX	246	299	7.25E-08	8.41E-05	0.0,0.342,0.288	0.829,1.0,1.0	-0.733
ENSMUSG00000030759	*Far1*	7	MEX	321	321	1.39E-06	0.000644	0.621,0.82,0.614	0.935,0.931,0.884	-0.232
ENSMUSG00000009995	*Taz*	X	MEX	202	240	5.06E-07	0.000392	0.978,1.0,0.955	0.834,0.873,0.717	0.17
ENSMUSG00000023393	*Slc17a9*	2	RI	574	149	0.00011125	0.033899	1.0,0.645,1.0	0.493,0.206,0.661	0.428
ENSMUSG00000026107	*Nabp1*	1	RI	8.33E+02	149	0.00024484	0.049735	0.953,0.79,0.94	0.811,0.566,0.628	0.226

ENSMUSG00000035875	*AI182371*	2	RI	416	149	0.00011039	0.033899	0.038,0.0,0.021	0.741,0.589,0.152	-0.474
ENSMUSG00000025237	*Parp6*	9	RI	793	149	3.3862E-14	1.03E-10	1.0,1.0,1.0	0.33,0.375,0.554	0.58
ENSMUSG00000056962	*Jmjd6*	11	RI	670	149	0.0001075	0.033899	0.97,1.0,0.912	0.788,0.552,0.88	0.221
ENSMUSG00000000915	*Hip1r*	5	RI	580	149	1.399E-05	0.007104	0.435,0.673,0.339	1.0,0.673,1.0	-0.409
ENSMUSG00000004266	*Ptpn6*	6	RI	3.20E+02	149	0.0002319	0.049735	0.344,0.449,0.651	0.055,0.162,0.032	0.398
ENSMUSG00000020692	*Nle1*	11	RI	1105	149	2.5509E-05	0.011104	0.702,0.859,0.69	0.288,0.327,0.283	0.451
ENSMUSG00000024403	*Atp6v1g2*	17	RI	556	149	1.1675E-05	0.007104	1.0,0.931,1.0	0.287,0.493,0.898	0.418
ENSMUSG00000011832	*Evi5l*	8	RI	229	149	0.00022927	0.049735	0.066,0.075,0.061	0.256,0.188,0.249	-0.164
ENSMUSG00000015880	*Ncapg*	5	RI	1479	149	3.7788E-06	0.002879	0.194,0.084,0.172	0.015,0.049,0.032	0.118
ENSMUSG00000038781	*Stap2*	17	RI	4.35E+02	149	0.00022295	0.049735	0.124,0.0,0.0	0.181,0.327,0.32	-0.235
ENSMUSG00000026170	*Cyp27a1*	1	RI	261	149	0.00020156	0.049735	0.533,0.65,0.727	1.0,1.0,1.0	-0.363
ENSMUSG00000062647	*Rpl7a*	2	RI	335	149	2.4985E-06	0.002538	1.0,1.0,0.18	0.185,0.098,0.092	0.602
ENSMUSG00000047534	*Mis18bp1*	12	RI	1124	149	4.5186E-12	6.88E-09	0.603,0.642,0.581	0.141,0.223,0.264	0.399
