# Supplementary material for: SRSF1 regulates primordial follicle formation and number determination during meiotic prophase I
Source: BMC Biol. 2023 Mar 8;21:49. doi: 10.1186/s12915-023-01549-7 (PMC9993595; doi:10.1186/s12915-023-01549-7)
Supplement: Supplementary file 6 — Additional file 6: Table 3. Primer sequences were used in this study. [file 12915_2023_1549_MOESM6_ESM.pdf]

Additional file 6: Table 3. Primer sequences are used in this study.

Target	Sequence(5' to 3')	Application
Srsf1 -GT-F	ACTAATGTGGGAAGAATGGC	Genotyping
Srsf1 -GT-FKO	TTCGCCTTCGTTGAGTTC	
Srsf1 -GT-R	AAACTATTGCTCCCATCTGC	
Stra8-GFPcre- F	ACTCCAAGCACTGGGCAGAA	
Stra8-GFPcre -R1	GCCACCATAGCAGCATCAAA	
Stra8-GFPcre -R2	CGTTTACGTCGCCGTCCAG	
Srsf1 -F	AGGAGGATTGAGGAGGATCAG	RT-qPCR
Srsf1 -R	CGCTCCATGAATCCTGGTAA	
Gapdh -F	GGGTCCCAGCTTAGGTTCAT	
Gapdh -R	CCCAATACGGCCAAATCCGT	
Vasa -F	GCCGTGGAGGATTTGGTCTA	
Vasa -R	AAGTGTCACCATTGCCTGAAT	
Jagged1- F	CCTGCGAGCCAAGGTGTG	
Jagged1 -R	CTCCACCACAACAGTTCCCA	
Kit -F	GGAGTGTAAGGCCTCCAACG	
Kit -R	GGCCTGGATTTGCTCTTTGTT	
Rac1 -F	CAAAGACAAGCCGATTGCCG	
Rac1 -R	TCGCACTTCAGGATACCACTTT	
Figla- F	AGCGAAGGTCTCAGAGAAACAA	
Figla -R	AGCTGGTAGGTTGGGTAGCA	
Lhx8- F	GACAAATACCTCCTCAAGGTGAA	
Lhx8 -R	CGAGTGGATGTGCCTGCC	
Nobox -F	TGGGGCACTAGTATCGCCT	
Nobox -R	GGAGAGCTGGAATGAACCCA	
Sohlh1 -F	ATTACGGGATGCAGCAAGAC	
Sohlh1 -R	GCTGACCACGTTTCTCCGAA	
Rec8 -F	CAGCGGAGAGCTTCACTACC	
Rec8 -R	TTGTATCCCTTCCACCTGCG	
Rad21l -F	ATATTTCGGAGCCCCTTGCTC	
Rad21l -R	TCAGGTTCATCCCCAAAGCTC	
Hormad1- F	GGAGCTTCTGAAATCAAAGAACCAA	
Hormad1 -R	TTTTTCCGCTTCTTGTTTTGCT	
Hormad2 -F	GTGGGTTCTGGGGTCTTCAA	
Hormad2 -R	TTTCAACAATTCCAACCTGAAGC	
Syce1 -F	CAGAAACGGCAGAGGCTGAA	
Syce1 -R	TTAGGTCCTGCTTGATGGGC	
Syce2- F	GCGCCTCCCGTGGAGTTA	
Syce2 -R	CTGGCAACTGGCACTCGG	
Sycp1- F	TGCCAAAAGCATTTTACCTACAACA	
Sycp1 -R	TTCCTCTGAAACCAGGCTCAAG	
Sycp3 -F	GCGTTCAGCCAATCAGCAG	
Sycp3 -R	TCTTTAGATGTTTGCTCAGCGG	
Psmc3ip- F	TTTATTTTGCCGATCAGAACCAGT	
Psmc3ip -R	TTTCTGCATCTCAGGAGTGGTC	
Tdrd9 -F	TGTGTGATGGGCCAAATGGA	
Tdrd9 -R	TCAACCGACAGAACAAGCCTA	
Plk1 -F	CCCACCATTCACGAGTTGCT	
Plk1 -R	GGGGGTTCTCCACACCTTTATT	
Atm- F	GTGTGATTTTTCAGGGGATTTGG	
Atm -R	TTGTCTCAGCTGCTTGTTCAC	
Dmc1- F	GGCTGAACGCCAGCAAAAAT	
Dmc1 -R	GGGATCAGCCTGAAAGGTCA	
Rad51 -F	GACTTGCTGATGAGTTTGGTGT	
Rad51 R	CCCCTCTTCCTTTTCTCAGGT	
Msh5 -RT-F	ATGGCTTTCAGAGCGACCCC	
Msh5 -RT-R	TATCCCGAGCTCCACAGCA	
Six6os1 -RT-F	AGCGCTGAAGACACCTGAAT	
Six6os1 -RT-R	GTGAAGGCATCTTCTCCTTCTGA	
Six6os1 -F	AGCAAATGTTTCTAAGCTACGTGAA	RT-qPCR & RIP-qPCR
Six6os1 -R	TCACATGTTGGCTTCCAGTTAC	
Msh5 -F	CACCCCTCGGTGTACAAAGT	
Msh5 -R	GTGTAAACCACAGCCTGAGC	
