# Supplementary material for: SRSF1 regulates primordial follicle formation and number determination during meiotic prophase I
Source: BMC Biol. 2023 Mar 8;21:49. doi: 10.1186/s12915-023-01549-7 (PMC9993595; doi:10.1186/s12915-023-01549-7)
Supplement: Supplementary file 7 — Additional file 7: Table 4. Antibodies were used in this study. [file 12915_2023_1549_MOESM7_ESM.pdf]

Additional file 7: Table 4. Antibodies were used in this study.

Name	Cat.NO.	Source	Company	Dilution
VASA	ab13840	Rabbit polyclonal	abcam	IF, 1: 800
VASA	ab27591	Mouse monoclonal	abcam	IF, 1: 800
GAPDH	60004-1-Ig	Mouse monoclonal	Proteintech	WB, 1: 10000
ACTB	81115-1-RR	Rabbit monoclonal	Proteintech	WB, 1: 20000
SYCP1	ab15090	Rabbit polyclonal	abcam	IF, 1: 500
SYCP3 (AlexaFluor® 488)	ab205846	Mouse monoclonal	abcam	IF, 1: 500
SYCP3	sc-74569	Mouse monoclonal	Santa Cruz	IF, 1: 500
RPA1		Rabbit polyclonal	Luo LAB	IF, 1: 500
SRSF1	12929-2-AP	Rabbit polyclonal	Proteintech	WB, 1:1000 IF, 1:200
SRSF1	sc-33652	Mouse monoclonal	Santa Cruz	IF, 1:500
γH2AX (AlexaFluor® 555)	05-636-AF555	Mouse monoclonal	Millipore	IF, 1:800
DMC1	13714-1-AP	Rabbit polyclonal	Proteintech	IF, 1:500
MLH1	550838	Mouse monoclonal	BD Pharmingen™	IF, 1: 50
CREST	HCT-0100	Human serum	ImmunoVision	IF, 1: 100
HORMAD1	13917-1-AP	Rabbit polyclonal	Proteintech	IF, 1: 200
GM130	610822	Mouse monoclonal	BD Pharmingen™	IF, 1: 50
KIT	ab256345	Rabbit monoclonal	abcam	IF, 1: 2000
JAGGED1	sc-390177	Mouse monoclonal	Santa Cruz	IF, 1: 400
MSY2	sc-393840	Mouse monoclonal	Santa Cruz	IF, 1: 400
MSH5	bs-17852R	Rabbit polyclonal	Bioss	WB, 1: 1000
SIX6OS1	TD12863S	Rabbit polyclonal	Abmart	WB, 1:1000 IF, 1:200
GAR-647	bs-0295G-AF647	Goat polyclonal	Bioss	IF, 1: 500
GAH-647	bs-0297G-AF647	Goat polyclonal	Bioss	IF, 1: 500
GAR-488	A11034	Goat polyclonal	Life Technologies	IF, 1: 500
GAM-594	A32742	Goat polyclonal	Life Technologies	IF, 1: 500
DAR-594	A-21207	Donkey polyclonal	Life Technologies	IF, 1: 500
DAM-488	A-21202	Donkey polyclonal	Life Technologies	IF, 1: 500
GAR-HRP	7074P2	Goat polyclonal	CST	WB, 1: 2000
GAM-HRP	7076P2	Goat polyclonal	CST	WB, 1: 2000
